# Crystal structures of (1,4,7,10-tetra­aza­cyclo­dodecane-κ^4^ *N*)bis­(tri­cyano­methanido-κ*N*)nickel and (1,4,7,10-tetra­aza­cyclo­dodecane-κ^4^ *N*)(tri­cyano­methanido-κ*N*)copper tri­cyano­methanide

**DOI:** 10.1107/S2056989015009524

**Published:** 2015-05-23

**Authors:** Jun Luo, Xin-Rong Zhang, Li-Juan Qiu, Feng Yang, Bao-Shu Liu

**Affiliations:** a School of Pharmacy, Second Military Medical University, Shanghai 200433, People’s Republic of China

**Keywords:** crystal structures, tri­cyano­methanide, 1,4,7,10-tetra­aza­cyclo­dodeca­ne, nickel complex, copper complex

## Abstract

The structures of two mononuclear transition-metal complexes with tri­cyano­methanide (tcm^−^) and 1,4,7,10-tetra­aza­cyclo­dodecane (cyclen) ligands, [Ni(C_4_N_3_)_2_(C_8_H_20_N_4_)], (I), and [Cu(C_4_N_3_)(C_8_H_20_N_4_)](C_4_N_3_), (II), are reported. In the neutral complex (I), the nickel cation is coordinated by one cyclen ligand and two monodentate N-bound tcm^−^ anions in a distorted octa­hedral geometry. The tcm^−^ ligands are mutually *cis*. The Cu^II^ atom in (II) displays a distorted tetra­gonal–pyramidal geometry, with the four N-donor atoms of the cyclen ligand in the equatorial plane, and one tcm^−^ anion bound through a single N atom in an axial site, forming a monocation. The second tcm^−^ molecule acts as a counter-ion not directly coordinating to the copper cation. In both (I) and (II), extensive series of N—H⋯N and C—H⋯N hydrogen bonds generate three-dimensional network structures.

## Abstract 2

Two new nickel and copper tri­cyano­methanide (tcm^−^) complexes with 1,4,7,10-tetra­aza­cyclo­dodecane (cyclen) as a co-ligand have been synthesized and structurally characterized.

## Chemical context   

Coordination polymers constructed by the tri­cyano­methanide anion (tcm^−^) have attracted considerable inter­est due to their fascinating structural characteristics (Hunt *et al.*, 2015 ▸; Hodgson *et al.*, 2014 ▸; Chainok *et al.*, 2012 ▸; Vreshch *et al.*, 2013 ▸) and inter­esting magnetic properties (Luo *et al.*, 2014 ▸; Herchel *et al.*, 2014 ▸; Váhovská *et al.*, 2014 ▸). To date, with the exception of a doubly inter­penetrated (6,3) sheet, observed in Ag(tcm)_2_^−^ (Abrahams *et al.*, 2003 ▸), most binary tcm^−^ complexes display a rutile-like structure (Manson *et al.*, 2000 ▸, 1998 ▸; Hoshino *et al.*, 1999 ▸; Feyerherm *et al.*, 2004 ▸). To gain an insight into the influence of co-ligands on the structural and magnetic properties of tcm^−^ complexes, various co-ligands, such as hexa­methyl­ene­tetra­mine, 4,4-bipyridyl and 1,2-di(pyridin-4-yl)ethane have been introduced to the binary tcm complexes. Among the Cu^I^ or Cd^II^ tcm^−^ complexes with such co-ligands, numerous structural types ranging from doubly inter­penetrated (4,4) sheets to three-dimensional rutile networks have been observed (Batten *et al.*, 2000 ▸, 1998 ▸). By contrast, modification of the Mn^II^–tcm binary system with 4,4-bipyridyl as a co-ligand leads to the formation of a one-dimensional chain-like structure (Manson *et al.*, 2004 ▸). In addition, the Julve group (Yuste *et al.*, 2007 ▸, 2008 ▸) recently reported the polymeric structures of copper tcm^−^ complexes with co-ligands such as bis­(2-pyrid­yl)pyrazine, 2,2′-bi­pyrazine and 2,3,5,6-tetra­kis­(2-pyrid­yl)pyrazine and found them to have inter­esting magnetic properties. 1,4,7,10-Tetra­aza­cyclo­dodecane (cyclen) is a novel co-ligand with four potential nitro­gen donor atoms. However, no tcm^−^ complexes incorporating cyclen as a co-ligand have been reported previously. As part of our systematic investigation of the effect of cyclen as a co-ligand on the structures and properties of tcm^−^ complexes, we have prepared two new tcm^−^ complexes and we report herein the syntheses and crystal structures of Ni(cyclen)(C_4_N_3_)_2_ (I) and [Cu(cyclen)(C_4_N_3_)]^+^(C_4_N_3_)^−^ (II).
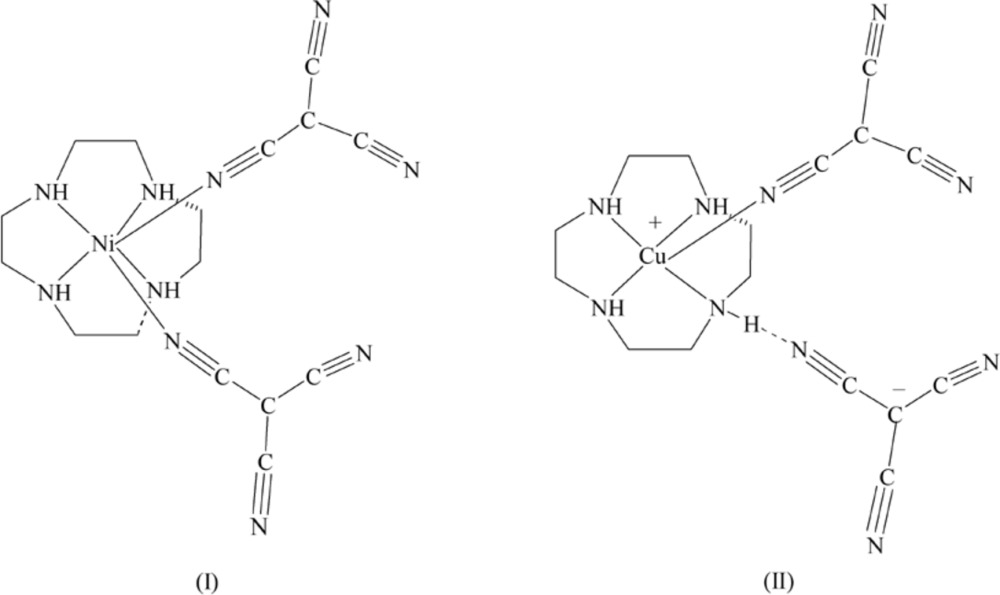


## Structural commentary   

**Figure 1:**
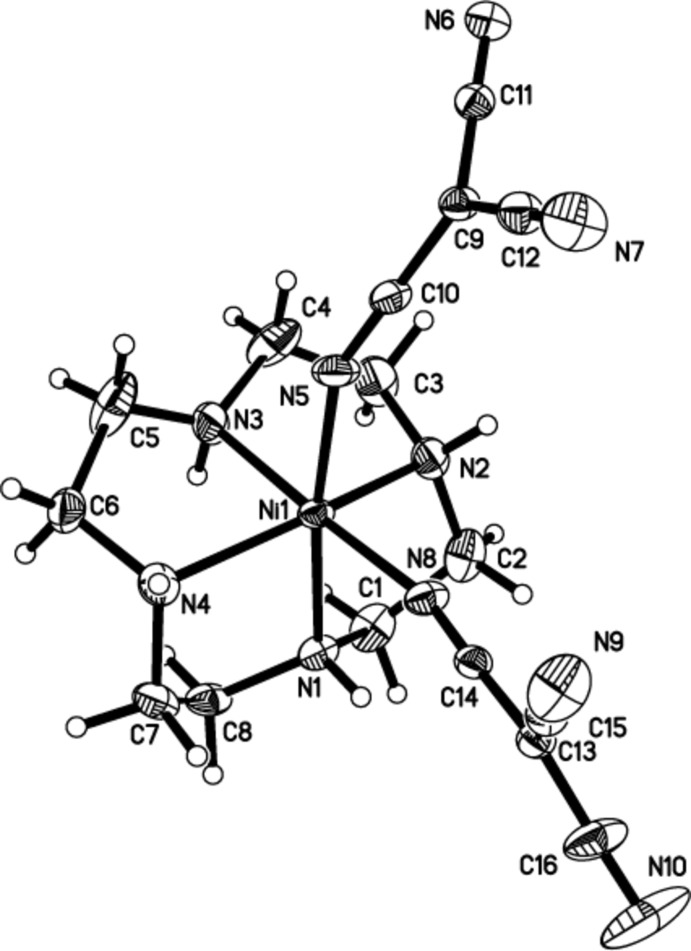
View of the mol­ecule of (I), showing the atom-labelling scheme. Displacement ellipsoids are drawn at the 50% probability level.

In (I), the nickel cation binds to the four N atoms of the cyclen and two N atoms of two tcm^−^ anions, forming a distorted octa­hedral geometry with the tcm^−^ ligands mutually *cis*. The equatorial plane is therefore formed by two N atoms (N1, N3) of the cyclen unit and the N5 and N8 atoms of the coordinating tcm^−^ anions. The apical sites are occupied by N2 and N4 from the cyclen ligand, Fig. 1 ▸.

**Figure 2:**
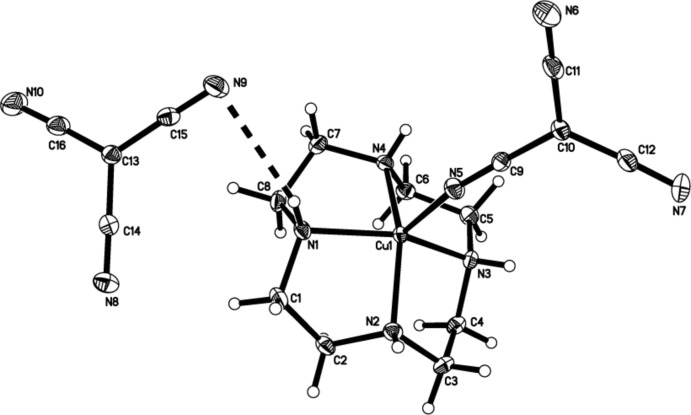
A view of the mol­ecule of (II), showing the atom-labelling scheme. Displacement ellipsoids are drawn at the 50% probability level. The hydrogen bond between the cation and anion is shown as a dashed line.

In (II), the copper cation is also bound to the four N atoms (N1, N2, N3, N4) of a cyclen ligand but in the basal plane with the N5 atom of the tcm^−^ ligand in an apical site, forming a five-coordinate cation with a distorted square-pyramidal coordin­ation geometry. The second tcm^−^ anion does not enter the inner coordination sphere of the metal (Fig. 2 ▸), but acts as a counter-anion that is linked to the cation in the asymmetric unit through an N1—H1⋯N9 hydrogen bond (Fig. 2 ▸).

The Ni—N(cyclen) distances in (I) [2.051 (3)–2.134 (3) Å] show some variation, but these values are similar to the corresponding distances in other polyamine-containing nickel complexes (Shirase *et al.*, 2009 ▸; Patel *et al.*, 2008 ▸). The Ni—N(tcm) distances, 2.062 (3) and 2.101 (3) Å, Table 1 ▸, of (I) are not unusual, and these data are comparable to the corres­ponding distances in other closely related nickel complexes with tcm^−^ ligands (Luo *et al.*, 2014 ▸, 2006 ▸).

In (II), the Cu—N(cyclen) distances range from 2.014 (2) to 2.034 (2) Å, and are similar to distances found in other reported copper complexes with polyamine co-ligands (Qi *et al.*, 2014 ▸; Belda *et al.*, 2013 ▸). In (II), the Cu—N(tcm) distance [2.097 (2) Å, Table 2 ▸) is also similar to the distances found in previously reported copper tcm^−^ complexes (Yuste *et al.*, 2007 ▸, 2008 ▸).

In (I), the N—Ni—N angles, involving two *cis-*related basal N atoms and the N(apical)—Ni—N(basal) angle range from 84.9 (1) to 97.3 (1)° and 81.6 (1) to 101.2 (1)°, respectively. The corresponding values for (II) are 85.57 (9) to 86.11 (9)° and 101.87 (9) to 109.54 (9)°, respectively, again indicating that the distortion from the octa­hedral and square-pyramidal geom­etries in (I) and (II) is not particularly severe.

Each tcm^−^ ligand is almost planar, with the mean deviations from the planes through all atoms of the coordinating tcm^−^ anions being 0.0128 and 0.0322 Å, respectively in (I). For (II), the corresponding deviations from the planes of the coordin­ating tcm^−^ anion and the tcm^−^ counter-anion are 0.0211 and 0.0074 Å respectively. Bond lengths and angles within the anions are also in good agreement with those found in other tcm^−^ complexes (Batten *et al.*, 1999 ▸; Yuste *et al.*, 2008 ▸).

## Supra­molecular features   

**Figure 3:**
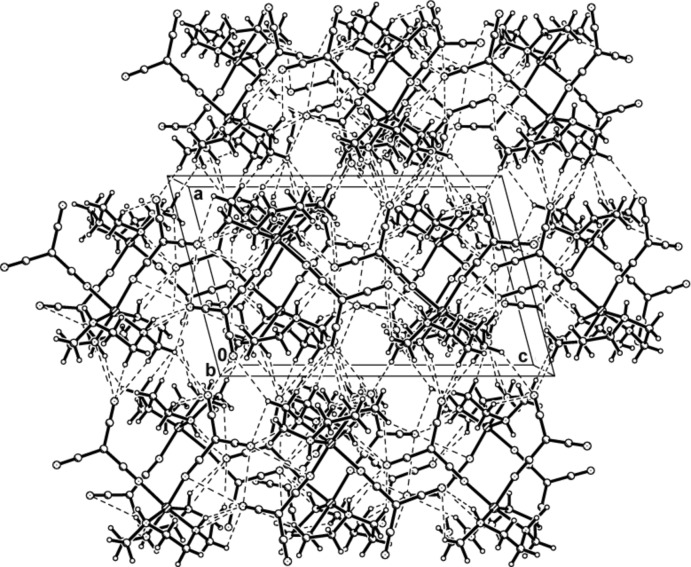
The three-dimensional network of (I), formed by hydrogen-bonding inter­actions, viewed along the *b* axis. Hydrogen bonds are drawn as dashed lines.

In the crystal structure of (I), each complex mol­ecule is linked to five others by a series of N—H⋯N and C—H⋯N hydrogen bonds. N1—H1⋯N10 and N2—H2⋯N6 hydrogen bonds each form inversion dimers, joining the complex mol­ecule to two neighbouring mol­ecules and generating 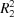
(16) ring motifs (Bernstein *et al.*, 1995 ▸). N3—H3⋯N7 and N4—H4⋯N6 hydrogen bonds link two additional complex mol­ecules. A C4—H4*B*⋯N9 contact involves the fifth complex. This array of contacts combines to generate an extensive three-dimensional network (Fig. 3 ▸, Table 3 ▸).

**Figure 4:**
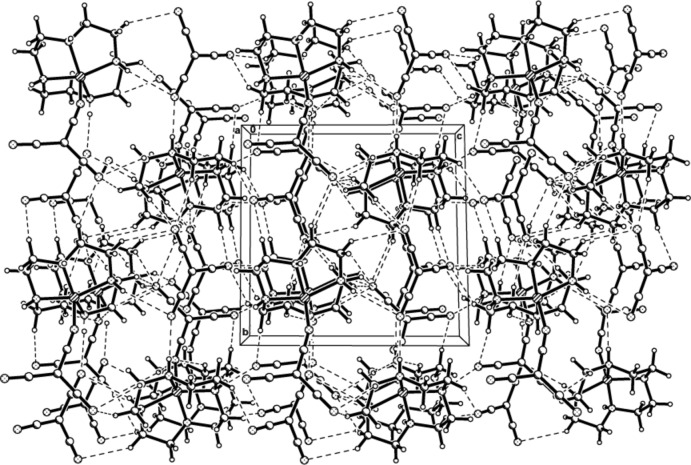
The three-dimensional network of (II), formed by hydrogen-bonding inter­actions, viewed along the *a* axis. Hydrogen bonds are drawn as dashed lines.

In the crystal structure of (II), N1—H1⋯N6 and N3—H3⋯N7 hydrogen bonds each form inversion dimers, also linking the complex cation to two neighbouring cations and generating 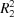
(16) ring motifs. Each complex mol­ecule is also linked *via* N—H⋯N and C—H⋯N hydrogen bonds to two adjacent complex cations and three tcm^−^ anions, forming another extensive three-dimensional network (Fig. 4 ▸, Table 4 ▸).

## Database survey   

Structures of transition-metal complexes with two or more tcm^−^ ligands are quite common with 47 unique compounds recorded in the Cambridge Crystallographic Database (Version 5.36, November 2014 with two updates; Groom & Allen, 2014 ▸). Of these the majority, 35, are polymeric or oligomeric systems. Five of these are Ni^II^ complexes but only two of them [tris­(2-amino­eth­yl)amine]­bis­(tri­cyano­meth­an­ide)nickel(II) (Luo *et al.*, 2014 ▸) and *cis*-bis­(tri­cyano­methanide-κ*N*)[tris­(2-amino­eth­yl)amine-κ^4^ *N*]nickel(II) (Potočňák *et al.*, 2007 ▸) are mononuclear, each with a distorted octa­hedral coordination environment and with the tcm^−^ ligands mutually *cis*.

The number of transition-metal complexes with the cyclen ligand is huge, with 116 unique hits in the current Database. Among these, there are twenty Ni^II^ complexes and nine Cu^II^ complexes. Representative Ni complexes include [Ni(cyclen)]_2_[Pt(CN)_4_]_2_·6H_2_O and [Ni(cyclen)]_2_[(Ni(CN)_4_)]_2_·6H_2_O (Yeung *et al.*, 2006 ▸), while examples of Cu complexes are [Cu(cyclen)(Au(CN)_2_)]^+^·[Au(CN)_2_]^−^ (Yeung *et al.*, 2000 ▸) and [Cu(cyclen)(NO_3_)]^+^·NO_3_^−^ (Clay *et al.*, 1979 ▸). However, no complexes containing a transition metal coordinated by both cyclen and tcm^−^ ligands were found.

## Synthesis and crystallization   

A 5 ml ethanol solution of 1,4,7,10-tetra­aza­cyclo­dodecane (0.10 mmol, 17.23 mg) and 2 ml of a green aqueous solution of nickel(II) nitrate (0.10 mmol, 29.08 mg) were mixed and stirred for 5 min; the resulting solution was purple. A 3 ml ethanol–water solution (EtOH:H_2_O = 2:1, *v*:*v*) of potassium tri­cyano­methanide (0.20 mmol, 25.83 mg) was then added. After stirring for another 5 min, the purple solution was filtered and the filtrate slowly evaporated in air. After two weeks, purple block-like crystals of (I) were isolated in 31% yield. Analysis calculated for C_16_H_20_N_10_Ni: C 46.75%, H 4.90%, N 34.07%. Found C 46.91%, H 5.03%, N 34.26%. Using copper(II) nitrate instead of nickel(II) nitrate, blue block-like crystals of (II) were prepared in a similar manner in 25% yield. Analysis calculated for C_16_H_20_N_10_Cu: C 46.20%, H 4.85%, N 33.67%. Found C 46.42%, H 5.01%, N 33.85%.

## Refinement   

Crystal data, data collection and structure refinement details are summarized in Table 5 ▸. In (I), the H1, H2, H3 and H4 atoms bound to the amine N atoms were found in a difference Fourier map and refined freely with isotropic displacement parameters. The N—H distances ranged from 0.90 (2) to 0.95 (2) Å. H atoms bound to carbon were constrained to an ideal geometry with C—H distances of 0.99 Å, and with *U*_iso_ = 1.2*U*_eq_(C) for CH_2_. In (II), the amine H1, H2, H3 and H4 atoms and the H atoms linked to carbon were refined similarly. The N—H distances were in the range 0.84 (3) to 0.94 (2) Å.

## Supplementary Material

Crystal structure: contains datablock(s) global, I, II. DOI: 10.1107/S2056989015009524/sj5461sup1.cif

Structure factors: contains datablock(s) I. DOI: 10.1107/S2056989015009524/sj5461Isup2.hkl

Structure factors: contains datablock(s) II. DOI: 10.1107/S2056989015009524/sj5461IIsup3.hkl

Supporting information file. DOI: 10.1107/S2056989015009524/sj5461sup4.pdf

Supporting information file. DOI: 10.1107/S2056989015009524/sj5461sup5.pdf

Supporting information file. DOI: 10.1107/S2056989015009524/sj5461sup6.pdf

CCDC references: 1401692, 1401691

Additional supporting information:  crystallographic information; 3D view; checkCIF report

## Figures and Tables

**Table 1:** Selected geometric parameters (Å, °) for (I)

Ni1—N1	2.051 (3)	Ni1—N5	2.101 (3)
Ni1—N8	2.062 (3)	Ni1—N2	2.125 (3)
Ni1—N3	2.080 (3)	Ni1—N4	2.134 (3)
			
N1—Ni1—N8	87.4 (1)	N3—Ni1—N2	81.6 (1)
N1—Ni1—N3	97.3 (1)	N5—Ni1—N2	95.4 (1)
N8—Ni1—N3	175.3 (1)	N1—Ni1—N4	82.8 (1)
N1—Ni1—N5	171.8 (1)	N8—Ni1—N4	98.1 (1)
N8—Ni1—N5	84.9 (1)	N3—Ni1—N4	82.0 (1)
N3—Ni1—N5	90.4 (1)	N5—Ni1—N4	101.2 (1)
N1—Ni1—N2	83.0 (1)	N2—Ni1—N4	156.7 (1)
N8—Ni1—N2	99.7 (1)		

**Table 2:** Selected geometric parameters (Å, °) for (II)

Cu1—N2	2.014 (2)	Cu1—N1	2.034 (2)
Cu1—N3	2.022 (2)	Cu1—N5	2.097 (2)
Cu1—N4	2.029 (2)		
			
N2—Cu1—N3	85.61 (9)	N4—Cu1—N1	85.79 (9)
N2—Cu1—N4	148.42 (9)	N2—Cu1—N5	107.9 (1)
N3—Cu1—N4	85.57 (9)	N3—Cu1—N5	101.87 (9)
N2—Cu1—N1	86.11 (9)	N4—Cu1—N5	103.57 (9)
N3—Cu1—N1	148.55 (9)	N1—Cu1—N5	109.54 (9)

**Table 3:** Hydrogen-bond geometry (Å, °) for (I)

*D*—H⋯*A*	*D*—H	H⋯*A*	*D*⋯*A*	*D*—H⋯*A*
C8—H8*B*⋯N7^i^	0.99	2.74	3.665 (5)	156
N3—H3⋯N7^i^	0.95 (2)	2.45 (3)	3.330 (5)	154 (4)
N1—H1⋯N10^ii^	0.90 (2)	2.11 (3)	2.907 (5)	148 (4)
N2—H2⋯N6^iii^	0.91 (2)	2.22 (3)	3.064 (4)	155 (4)
C4—H4*B*⋯N9^iv^	0.99	2.57	3.467 (5)	151
N4—H4⋯N6^v^	0.90 (2)	2.70 (4)	3.372 (4)	133 (4)
C7—H7*B*⋯N6^v^	0.99	2.70	3.397 (5)	128

Symmetry codes: (i) 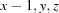; (ii) 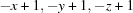; (iii) 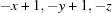; (iv) 
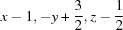
; (v) 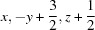.

**Table 4:** Hydrogen-bond geometry (Å, °) for (II)

*D*—H⋯*A*	*D*—H	H⋯*A*	*D*⋯*A*	*D*—H⋯*A*
N1—H1⋯N9	0.87 (2)	2.79 (3)	3.525 (4)	143 (3)
C1—H1*B*⋯N6^i^	0.99	2.54	3.273 (4)	131
N1—H1⋯N6^i^	0.87 (2)	2.60 (3)	3.206 (4)	127 (3)
N4—H4⋯N9^ii^	0.84 (3)	2.24 (4)	3.067 (3)	168 (3)
N3—H3⋯N7^iii^	0.92 (2)	2.05 (2)	2.928 (3)	159 (4)
N2—H2⋯N8^iv^	0.94 (2)	2.19 (2)	3.003 (3)	144 (3)
C3—H3*B*⋯N10^v^	0.99	2.64	3.531 (4)	150
C8—H8*B*⋯N8^vi^	0.99	2.56	3.538 (4)	171
C3—H3*A*⋯N10^vi^	0.99	2.64	3.460 (4)	140

Symmetry codes: (i) 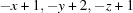; (ii) 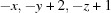; (iii) 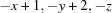; (iv) 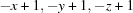; (v) 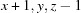; (vi) 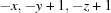.

**Table 5:** Experimental details

	(I)	(II)
Crystal data		
Chemical formula	[Ni(C_4_N_3_)_2_(C_8_H_20_N_4_)]	[Cu(C_4_N_3_)(C_8_H_20_N_4_)](C_4_N_3_)
*M*_r_	411.13	415.96
Crystal system, space group	Monoclinic, *P*2_1_/*c*	Triclinic, *P* 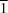
Temperature (K)	173	173
*a*, *b*, *c* (Å)	10.6300 (12), 11.0150 (12), 17.1771 (18)	7.4074 (15), 11.552 (2), 11.625 (2)
α, β, γ (°)	90, 104.828 (2), 90	89.187 (3), 88.236 (3), 78.579 (3)
*V* (Å^3^)	1944.3 (4)	974.6 (3)
*Z*	4	2
Radiation type	Mo *K*α	Mo *K*α
μ (mm^−1^)	1.02	1.14
Crystal size (mm)	0.06 × 0.05 × 0.04	0.10 × 0.07 × 0.06

Data collection		
Diffractometer	Bruker APEXII CCD	Bruker APEXII CCD
Absorption correction	Multi-scan (*SADABS*; Sheldrick, 1996 ▸)	Multi-scan (*SADABS*; Sheldrick, 1996 ▸)
*T*_min_, *T*_max_	0.670, 0.746	0.680, 0.746
No. of measured, independent and observed [*I* > 2σ(*I*)] reflections	12264, 4611, 3460	6936, 4267, 3639
*R*_int_	0.032	0.021
(sin θ/λ)_max_ (Å^−1^)	0.659	0.644

Refinement		
*R*[*F* ^2^ > 2σ(*F* ^2^)], *wR*(*F* ^2^), *S*	0.053, 0.136, 1.05	0.033, 0.110, 1.13
No. of reflections	4611	4267
No. of parameters	259	260
No. of restraints	12	3
H-atom treatment	H atoms treated by a mixture of independent and constrained refinement	H atoms treated by a mixture of independent and constrained refinement
Δρ_max_, Δρ_min_ (e Å^−3^)	1.05, −0.67	0.43, −0.30

Computer programs: *APEX2* and *SAINT* (Bruker, 2010 ▸), *SHELXS97* and *SHELXTL* (Sheldrick, 2008 ▸) and *SHELXL2013* (Sheldrick, 2015 ▸).

## supplementary crystallographic information

### (I) (1,4,7,10-Tetraazacyclododecane-κ4N)bis(tricyanomethanido-κN)nickel Crystal data

**Table:**

[Ni(C_4_N_3_)_2_(C_8_H_20_N_4_)]	*F*(000) = 856
*M**_r_* = 411.13	*D*_x_ = 1.405 Mg m^−^^3^
Monoclinic, *P*2_1_/*c*	Mo *K*α radiation, λ = 0.71073 Å
*a* = 10.6300 (12) Å	Cell parameters from 3344 reflections
*b* = 11.0150 (12) Å	θ = 2.5–27.8°
*c* = 17.1771 (18) Å	µ = 1.02 mm^−^^1^
β = 104.828 (2)°	*T* = 173 K
*V* = 1944.3 (4) Å^3^	Block, purple
*Z* = 4	0.06 × 0.05 × 0.04 mm

### (I) (1,4,7,10-Tetraazacyclododecane-κ4N)bis(tricyanomethanido-κN)nickel Data collection

**Table:**

Bruker APEXII CCD diffractometer	3460 reflections with *I* > 2σ(*I*)
φ and ω scans	*R*_int_ = 0.032
Absorption correction: multi-scan (*SADABS*; Sheldrick, 1996)	θ_max_ = 27.9°, θ_min_ = 2.0°
*T*_min_ = 0.670, *T*_max_ = 0.746	*h* = −10→13
12264 measured reflections	*k* = −14→14
4611 independent reflections	*l* = −20→22

### (I) (1,4,7,10-Tetraazacyclododecane-κ4N)bis(tricyanomethanido-κN)nickel Refinement

**Table:**

Refinement on *F*^2^	12 restraints
Least-squares matrix: full	Hydrogen site location: mixed
*R*[*F*^2^ > 2σ(*F*^2^)] = 0.053	H atoms treated by a mixture of independent and constrained refinement
*wR*(*F*^2^) = 0.136	*w* = 1/[σ^2^(*F*_o_^2^) + (0.0532*P*)^2^ + 3.2428*P*] where *P* = (*F*_o_^2^ + 2*F*_c_^2^)/3
*S* = 1.05	(Δ/σ)_max_ = 0.004
4611 reflections	Δρ_max_ = 1.05 e Å^−^^3^
259 parameters	Δρ_min_ = −0.67 e Å^−^^3^

### (I) (1,4,7,10-Tetraazacyclododecane-κ4N)bis(tricyanomethanido-κN)nickel Special details

**Table:**

Geometry. All e.s.d.'s (except the e.s.d. in the dihedral angle between two l.s. planes) are estimated using the full covariance matrix. The cell e.s.d.'s are taken into account individually in the estimation of e.s.d.'s in distances, angles and torsion angles; correlations between e.s.d.'s in cell parameters are only used when they are defined by crystal symmetry. An approximate (isotropic) treatment of cell e.s.d.'s is used for estimating e.s.d.'s involving l.s. planes.

### (I) (1,4,7,10-Tetraazacyclododecane-κ4N)bis(tricyanomethanido-κN)nickel Fractional atomic coordinates and isotropic or equivalent isotropic displacement parameters (Å^2^)

**Table:**

	*x*	*y*	*z*	*U*_iso_*/*U*_eq_	
Ni1	0.31796 (4)	0.67335 (4)	0.21935 (2)	0.02888 (13)	
N1	0.2130 (3)	0.6100 (3)	0.29576 (17)	0.0345 (6)	
H1	0.279 (3)	0.589 (4)	0.337 (2)	0.064 (14)*	
N2	0.2664 (3)	0.5030 (3)	0.16203 (18)	0.0443 (7)	
H2	0.339 (3)	0.472 (4)	0.151 (2)	0.055 (13)*	
N3	0.1611 (4)	0.7305 (3)	0.12737 (19)	0.0530 (9)	
H3	0.091 (4)	0.711 (3)	0.150 (2)	0.079*	
N4	0.2863 (3)	0.8444 (3)	0.26943 (19)	0.0394 (7)	
H4	0.365 (3)	0.881 (4)	0.284 (3)	0.073 (15)*	
C1	0.1384 (4)	0.5016 (4)	0.2615 (3)	0.0506 (10)	
H1A	0.0566	0.5259	0.2224	0.061*	
H1B	0.1159	0.4536	0.3048	0.061*	
C2	0.2200 (4)	0.4269 (4)	0.2202 (3)	0.0553 (11)	
H2A	0.2955	0.3931	0.2607	0.066*	
H2B	0.1680	0.3582	0.1916	0.066*	
C3	0.1642 (5)	0.5211 (5)	0.0857 (3)	0.0642 (13)	
H3A	0.1869	0.4734	0.0424	0.077*	
H3B	0.0802	0.4901	0.0925	0.077*	
C4	0.1492 (5)	0.6485 (4)	0.0617 (3)	0.0611 (13)	
H4A	0.2159	0.6691	0.0328	0.073*	
H4B	0.0628	0.6597	0.0237	0.073*	
C5	0.1683 (5)	0.8539 (4)	0.1225 (3)	0.0748 (17)	
H5A	0.0853	0.8850	0.0880	0.090*	
H5B	0.2384	0.8753	0.0965	0.090*	
C6	0.1952 (4)	0.9164 (4)	0.2047 (3)	0.0549 (11)	
H6A	0.2333	0.9975	0.2009	0.066*	
H6B	0.1119	0.9281	0.2195	0.066*	
C7	0.2311 (4)	0.8170 (4)	0.3387 (2)	0.0448 (9)	
H7A	0.1834	0.8888	0.3508	0.054*	
H7B	0.3027	0.7990	0.3869	0.054*	
C8	0.1402 (3)	0.7099 (4)	0.3202 (2)	0.0419 (9)	
H8A	0.1108	0.6868	0.3684	0.050*	
H8B	0.0628	0.7303	0.2762	0.050*	
N5	0.4473 (3)	0.7223 (3)	0.15041 (17)	0.0456 (8)	
N6	0.5442 (3)	0.6048 (3)	−0.07283 (18)	0.0410 (7)	
N7	0.8599 (3)	0.6841 (4)	0.1448 (2)	0.0676 (11)	
C9	0.6177 (3)	0.6745 (3)	0.07428 (19)	0.0313 (6)	
C10	0.5245 (3)	0.7011 (3)	0.11644 (19)	0.0341 (7)	
C11	0.5768 (3)	0.6366 (3)	−0.0066 (2)	0.0322 (7)	
C12	0.7506 (3)	0.6795 (4)	0.1130 (2)	0.0400 (8)	
N8	0.4834 (3)	0.6233 (3)	0.30541 (16)	0.0351 (6)	
N9	0.8889 (4)	0.7038 (4)	0.4340 (2)	0.0632 (11)	
N10	0.6565 (5)	0.4491 (7)	0.5383 (3)	0.121 (3)	
C13	0.6733 (3)	0.5974 (3)	0.42904 (19)	0.0324 (7)	
C14	0.5682 (3)	0.6129 (3)	0.36206 (18)	0.0300 (7)	
C15	0.7916 (4)	0.6567 (3)	0.4323 (2)	0.0387 (8)	
C16	0.6636 (4)	0.5171 (5)	0.4895 (2)	0.0639 (14)	

### (I) (1,4,7,10-Tetraazacyclododecane-κ4N)bis(tricyanomethanido-κN)nickel Atomic displacement parameters (Å^2^)

**Table:**

	*U*^11^	*U*^22^	*U*^33^	*U*^12^	*U*^13^	*U*^23^
Ni1	0.0232 (2)	0.0426 (2)	0.02079 (19)	0.00588 (18)	0.00552 (14)	0.00459 (17)
N1	0.0218 (13)	0.0488 (17)	0.0330 (15)	−0.0004 (12)	0.0069 (11)	0.0090 (13)
N2	0.0386 (17)	0.0516 (19)	0.0366 (16)	0.0118 (15)	−0.0017 (13)	−0.0061 (14)
N3	0.054 (2)	0.062 (2)	0.0366 (17)	0.0311 (18)	0.0001 (15)	0.0008 (15)
N4	0.0309 (15)	0.0434 (17)	0.0468 (17)	0.0001 (13)	0.0152 (13)	0.0001 (14)
C1	0.0335 (18)	0.053 (2)	0.063 (2)	−0.0105 (18)	0.0080 (17)	0.011 (2)
C2	0.044 (2)	0.045 (2)	0.068 (3)	−0.0018 (19)	−0.001 (2)	0.002 (2)
C3	0.057 (3)	0.075 (3)	0.046 (2)	0.022 (2)	−0.013 (2)	−0.016 (2)
C4	0.062 (3)	0.064 (3)	0.040 (2)	−0.017 (2)	−0.0205 (19)	0.0129 (19)
C5	0.076 (3)	0.044 (2)	0.078 (3)	−0.003 (2)	−0.028 (3)	0.016 (2)
C6	0.062 (3)	0.049 (2)	0.065 (3)	0.022 (2)	0.036 (2)	0.020 (2)
C7	0.0416 (19)	0.061 (2)	0.0325 (18)	0.0042 (19)	0.0109 (15)	−0.0095 (17)
C8	0.0279 (16)	0.070 (3)	0.0313 (17)	0.0059 (17)	0.0147 (14)	0.0073 (17)
N5	0.0432 (17)	0.069 (2)	0.0291 (15)	0.0105 (16)	0.0171 (13)	0.0107 (14)
N6	0.0416 (17)	0.0470 (18)	0.0360 (16)	−0.0025 (14)	0.0126 (13)	−0.0032 (13)
N7	0.0364 (18)	0.105 (3)	0.055 (2)	0.000 (2)	0.0007 (16)	−0.005 (2)
C9	0.0294 (15)	0.0376 (17)	0.0285 (15)	−0.0012 (14)	0.0103 (12)	0.0040 (14)
C10	0.0337 (17)	0.0421 (19)	0.0264 (15)	0.0010 (14)	0.0075 (13)	0.0079 (13)
C11	0.0275 (16)	0.0355 (17)	0.0356 (18)	−0.0026 (13)	0.0119 (13)	0.0037 (14)
C12	0.0337 (18)	0.051 (2)	0.0357 (18)	0.0001 (17)	0.0089 (14)	−0.0001 (16)
N8	0.0265 (14)	0.0517 (17)	0.0266 (13)	−0.0004 (13)	0.0061 (11)	0.0061 (12)
N9	0.044 (2)	0.064 (2)	0.069 (3)	−0.0160 (18)	−0.0102 (18)	0.0013 (19)
N10	0.062 (3)	0.217 (7)	0.075 (3)	−0.003 (4)	0.002 (2)	0.095 (4)
C13	0.0279 (16)	0.0429 (18)	0.0251 (15)	0.0065 (14)	0.0043 (12)	−0.0006 (13)
C14	0.0276 (15)	0.0385 (17)	0.0253 (15)	0.0039 (13)	0.0093 (12)	0.0003 (13)
C15	0.0366 (19)	0.0402 (19)	0.0315 (17)	0.0023 (16)	−0.0056 (14)	−0.0071 (14)
C16	0.0330 (19)	0.120 (4)	0.035 (2)	0.007 (2)	0.0033 (16)	0.028 (2)

### (I) (1,4,7,10-Tetraazacyclododecane-κ4N)bis(tricyanomethanido-κN)nickel Geometric parameters (Å, º)

**Table:**

Ni1—N1	2.051 (3)	C3—H3B	0.9900
Ni1—N8	2.062 (3)	C4—H4A	0.9900
Ni1—N3	2.080 (3)	C4—H4B	0.9900
Ni1—N5	2.101 (3)	C5—C6	1.529 (7)
Ni1—N2	2.125 (3)	C5—H5A	0.9900
Ni1—N4	2.134 (3)	C5—H5B	0.9900
N1—C8	1.467 (5)	C6—H6A	0.9900
N1—C1	1.470 (5)	C6—H6B	0.9900
N1—H1	0.90 (2)	C7—C8	1.506 (6)
N2—C2	1.482 (6)	C7—H7A	0.9900
N2—C3	1.486 (5)	C7—H7B	0.9900
N2—H2	0.91 (2)	C8—H8A	0.9900
N3—C5	1.366 (6)	C8—H8B	0.9900
N3—C4	1.425 (6)	N5—C10	1.146 (4)
N3—H3	0.95 (2)	N6—C11	1.155 (4)
N4—C7	1.487 (5)	N7—C12	1.152 (5)
N4—C6	1.501 (5)	C9—C12	1.399 (5)
N4—H4	0.90 (2)	C9—C10	1.400 (4)
C1—C2	1.499 (6)	C9—C11	1.409 (5)
C1—H1A	0.9900	N8—C14	1.151 (4)
C1—H1B	0.9900	N9—C15	1.150 (5)
C2—H2A	0.9900	N10—C16	1.140 (6)
C2—H2B	0.9900	C13—C16	1.388 (5)
C3—C4	1.460 (6)	C13—C14	1.394 (4)
C3—H3A	0.9900	C13—C15	1.406 (5)
			
N1—Ni1—N8	87.4 (1)	C4—C3—N2	112.3 (4)
N1—Ni1—N3	97.3 (1)	C4—C3—H3A	109.1
N8—Ni1—N3	175.3 (1)	N2—C3—H3A	109.1
N1—Ni1—N5	171.8 (1)	C4—C3—H3B	109.1
N8—Ni1—N5	84.9 (1)	N2—C3—H3B	109.1
N3—Ni1—N5	90.4 (1)	H3A—C3—H3B	107.9
N1—Ni1—N2	83.0 (1)	N3—C4—C3	113.9 (4)
N8—Ni1—N2	99.7 (1)	N3—C4—H4A	108.8
N3—Ni1—N2	81.6 (1)	C3—C4—H4A	108.8
N5—Ni1—N2	95.4 (1)	N3—C4—H4B	108.8
N1—Ni1—N4	82.8 (1)	C3—C4—H4B	108.8
N8—Ni1—N4	98.1 (1)	H4A—C4—H4B	107.7
N3—Ni1—N4	82.0 (1)	N3—C5—C6	113.1 (4)
N5—Ni1—N4	101.2 (1)	N3—C5—H5A	109.0
N2—Ni1—N4	156.7 (1)	C6—C5—H5A	109.0
C8—N1—C1	117.0 (3)	N3—C5—H5B	109.0
C8—N1—Ni1	109.9 (2)	C6—C5—H5B	109.0
C1—N1—Ni1	110.3 (2)	H5A—C5—H5B	107.8
C8—N1—H1	109 (3)	N4—C6—C5	112.3 (3)
C1—N1—H1	110 (3)	N4—C6—H6A	109.1
Ni1—N1—H1	99 (3)	C5—C6—H6A	109.1
C2—N2—C3	112.1 (4)	N4—C6—H6B	109.1
C2—N2—Ni1	106.1 (2)	C5—C6—H6B	109.1
C3—N2—Ni1	109.5 (3)	H6A—C6—H6B	107.9
C2—N2—H2	112 (3)	N4—C7—C8	110.7 (3)
C3—N2—H2	110 (3)	N4—C7—H7A	109.5
Ni1—N2—H2	107 (3)	C8—C7—H7A	109.5
C5—N3—C4	125.3 (4)	N4—C7—H7B	109.5
C5—N3—Ni1	107.5 (3)	C8—C7—H7B	109.5
C4—N3—Ni1	107.5 (3)	H7A—C7—H7B	108.1
C5—N3—H3	108 (2)	N1—C8—C7	106.9 (3)
C4—N3—H3	105 (2)	N1—C8—H8A	110.3
Ni1—N3—H3	101 (3)	C7—C8—H8A	110.3
C7—N4—C6	112.9 (3)	N1—C8—H8B	110.3
C7—N4—Ni1	106.2 (2)	C7—C8—H8B	110.3
C6—N4—Ni1	107.8 (2)	H8A—C8—H8B	108.6
C7—N4—H4	113 (3)	C10—N5—Ni1	153.1 (3)
C6—N4—H4	111 (3)	C12—C9—C10	120.5 (3)
Ni1—N4—H4	106 (3)	C12—C9—C11	120.0 (3)
N1—C1—C2	108.5 (3)	C10—C9—C11	119.4 (3)
N1—C1—H1A	110.0	N5—C10—C9	179.3 (4)
C2—C1—H1A	110.0	N6—C11—C9	179.3 (4)
N1—C1—H1B	110.0	N7—C12—C9	179.7 (5)
C2—C1—H1B	110.0	C14—N8—Ni1	166.7 (3)
H1A—C1—H1B	108.4	C16—C13—C14	119.9 (3)
N2—C2—C1	109.9 (3)	C16—C13—C15	120.2 (3)
N2—C2—H2A	109.7	C14—C13—C15	119.7 (3)
C1—C2—H2A	109.7	N8—C14—C13	177.8 (4)
N2—C2—H2B	109.7	N9—C15—C13	178.7 (4)
C1—C2—H2B	109.7	N10—C16—C13	178.5 (7)
H2A—C2—H2B	108.2		
			
C8—N1—C1—C2	164.6 (3)	C4—N3—C5—C6	−173.8 (4)
Ni1—N1—C1—C2	38.0 (4)	Ni1—N3—C5—C6	−46.3 (5)
C3—N2—C2—C1	−79.0 (4)	C7—N4—C6—C5	−124.4 (4)
Ni1—N2—C2—C1	40.5 (4)	Ni1—N4—C6—C5	−7.4 (4)
N1—C1—C2—N2	−53.1 (4)	N3—C5—C6—N4	36.6 (6)
C2—N2—C3—C4	130.8 (4)	C6—N4—C7—C8	80.8 (4)
Ni1—N2—C3—C4	13.3 (5)	Ni1—N4—C7—C8	−37.2 (3)
C5—N3—C4—C3	170.8 (5)	C1—N1—C8—C7	−170.0 (3)
Ni1—N3—C4—C3	43.3 (5)	Ni1—N1—C8—C7	−43.2 (3)
N2—C3—C4—N3	−38.4 (6)	N4—C7—C8—N1	54.2 (4)

### (I) (1,4,7,10-Tetraazacyclododecane-κ4N)bis(tricyanomethanido-κN)nickel Hydrogen-bond geometry (Å, º)

**Table:**

*D*—H···*A*	*D*—H	H···*A*	*D*···*A*	*D*—H···*A*
C8—H8*B*···N7^i^	0.99	2.74	3.665 (5)	156
N3—H3···N7^i^	0.95 (2)	2.45 (3)	3.330 (5)	154 (4)
N1—H1···N10^ii^	0.90 (2)	2.11 (3)	2.907 (5)	148 (4)
N2—H2···N6^iii^	0.91 (2)	2.22 (3)	3.064 (4)	155 (4)
C4—H4*B*···N9^iv^	0.99	2.57	3.467 (5)	151
N4—H4···N6^v^	0.90 (2)	2.70 (4)	3.372 (4)	133 (4)
C7—H7*B*···N6^v^	0.99	2.70	3.397 (5)	128

Symmetry codes: (i) *x*−1, *y*, *z*; (ii) −*x*+1, −*y*+1, −*z*+1; (iii) −*x*+1, −*y*+1, −*z*; (iv) *x*−1, −*y*+3/2, *z*−1/2; (v) *x*, −*y*+3/2, *z*+1/2.

### (II) (1,4,7,10-Tetraazacyclododecane-κ4N)(tricyanomethanido-κN)copper tricyanomethanide Crystal data

**Table:**

[Cu(C_4_N_3_)(C_8_H_20_N_4_)](C_4_N_3_)	*Z* = 2
*M**_r_* = 415.96	*F*(000) = 430
Triclinic, *P*1	*D*_x_ = 1.417 Mg m^−^^3^
*a* = 7.4074 (15) Å	Mo *K*α radiation, λ = 0.71073 Å
*b* = 11.552 (2) Å	Cell parameters from 3012 reflections
*c* = 11.625 (2) Å	θ = 2.5–27.2°
α = 89.187 (3)°	µ = 1.14 mm^−^^1^
β = 88.236 (3)°	*T* = 173 K
γ = 78.579 (3)°	Block, blue
*V* = 974.6 (3) Å^3^	0.10 × 0.07 × 0.06 mm

### (II) (1,4,7,10-Tetraazacyclododecane-κ4N)(tricyanomethanido-κN)copper tricyanomethanide Data collection

**Table:**

Bruker APEXII CCD diffractometer	3639 reflections with *I* > 2σ(*I*)
φ and ω scans	*R*_int_ = 0.021
Absorption correction: multi-scan (*SADABS*; Sheldrick, 1996)	θ_max_ = 27.2°, θ_min_ = 1.8°
*T*_min_ = 0.680, *T*_max_ = 0.746	*h* = −9→9
6936 measured reflections	*k* = −14→12
4267 independent reflections	*l* = −14→14

### (II) (1,4,7,10-Tetraazacyclododecane-κ4N)(tricyanomethanido-κN)copper tricyanomethanide Refinement

**Table:**

Refinement on *F*^2^	3 restraints
Least-squares matrix: full	Hydrogen site location: mixed
*R*[*F*^2^ > 2σ(*F*^2^)] = 0.033	H atoms treated by a mixture of independent and constrained refinement
*wR*(*F*^2^) = 0.110	*w* = 1/[σ^2^(*F*_o_^2^) + (0.0554*P*)^2^ + 0.4093*P*] where *P* = (*F*_o_^2^ + 2*F*_c_^2^)/3
*S* = 1.13	(Δ/σ)_max_ = 0.001
4267 reflections	Δρ_max_ = 0.43 e Å^−^^3^
260 parameters	Δρ_min_ = −0.30 e Å^−^^3^

### (II) (1,4,7,10-Tetraazacyclododecane-κ4N)(tricyanomethanido-κN)copper tricyanomethanide Special details

**Table:**

Geometry. All e.s.d.'s (except the e.s.d. in the dihedral angle between two l.s. planes) are estimated using the full covariance matrix. The cell e.s.d.'s are taken into account individually in the estimation of e.s.d.'s in distances, angles and torsion angles; correlations between e.s.d.'s in cell parameters are only used when they are defined by crystal symmetry. An approximate (isotropic) treatment of cell e.s.d.'s is used for estimating e.s.d.'s involving l.s. planes.

### (II) (1,4,7,10-Tetraazacyclododecane-κ4N)(tricyanomethanido-κN)copper tricyanomethanide Fractional atomic coordinates and isotropic or equivalent isotropic displacement parameters (Å^2^)

**Table:**

	*x*	*y*	*z*	*U*_iso_*/*U*_eq_	
Cu1	0.19693 (4)	0.78312 (3)	0.28777 (2)	0.01966 (11)	
N1	0.1556 (3)	0.70853 (19)	0.44341 (18)	0.0247 (5)	
N2	0.3779 (3)	0.63230 (19)	0.25312 (19)	0.0255 (5)	
H2	0.499 (3)	0.629 (3)	0.275 (3)	0.030 (8)*	
N3	0.1539 (3)	0.7915 (2)	0.11666 (19)	0.0262 (5)	
H3	0.244 (5)	0.823 (4)	0.078 (3)	0.069 (13)*	
N4	−0.0693 (3)	0.8686 (2)	0.30501 (19)	0.0235 (5)	
H4	−0.080 (5)	0.943 (3)	0.305 (3)	0.032 (9)*	
C1	0.2693 (4)	0.5872 (2)	0.4435 (2)	0.0309 (6)	
H1A	0.2057	0.5339	0.4899	0.037*	
H1B	0.3893	0.5877	0.4784	0.037*	
C2	0.2999 (4)	0.5431 (2)	0.3211 (2)	0.0284 (6)	
H2A	0.3864	0.4659	0.3191	0.034*	
H2B	0.1818	0.5331	0.2888	0.034*	
C3	0.3874 (4)	0.6128 (3)	0.1270 (2)	0.0320 (6)	
H3A	0.4172	0.5271	0.1109	0.038*	
H3B	0.4854	0.6495	0.0911	0.038*	
C4	0.2027 (4)	0.6673 (3)	0.0775 (2)	0.0309 (6)	
H4A	0.2107	0.6645	−0.0076	0.037*	
H4B	0.1076	0.6232	0.1047	0.037*	
C5	−0.0403 (4)	0.8503 (3)	0.0989 (2)	0.0314 (6)	
H5A	−0.0867	0.8182	0.0296	0.038*	
H5B	−0.0483	0.9362	0.0871	0.038*	
C6	−0.1553 (4)	0.8288 (2)	0.2029 (2)	0.0278 (6)	
H6A	−0.2830	0.8737	0.1960	0.033*	
H6B	−0.1588	0.7438	0.2102	0.033*	
C7	−0.1439 (4)	0.8372 (3)	0.4181 (2)	0.0309 (6)	
H7A	−0.2777	0.8392	0.4133	0.037*	
H7B	−0.1257	0.8950	0.4766	0.037*	
C8	−0.0455 (4)	0.7147 (3)	0.4526 (2)	0.0297 (6)	
H8A	−0.0816	0.6971	0.5326	0.036*	
H8B	−0.0804	0.6553	0.4015	0.036*	
N5	0.3467 (3)	0.9172 (2)	0.3085 (2)	0.0302 (5)	
N6	0.4994 (4)	1.2516 (2)	0.4130 (3)	0.0458 (7)	
N7	0.6191 (5)	1.1120 (3)	0.0561 (3)	0.0557 (9)	
C9	0.4066 (3)	1.0003 (2)	0.2868 (2)	0.0234 (5)	
C10	0.4822 (4)	1.0987 (2)	0.2587 (2)	0.0284 (6)	
C11	0.4908 (4)	1.1832 (2)	0.3437 (3)	0.0317 (6)	
C12	0.5559 (4)	1.1083 (3)	0.1476 (3)	0.0370 (7)	
N8	0.2233 (3)	0.4808 (2)	0.7239 (2)	0.0346 (6)	
N9	0.0455 (4)	0.8693 (2)	0.7001 (2)	0.0401 (6)	
N10	−0.2516 (5)	0.6493 (3)	0.9329 (3)	0.0533 (8)	
C13	0.0029 (4)	0.6708 (2)	0.7860 (2)	0.0261 (5)	
C14	0.1277 (4)	0.5668 (2)	0.7519 (2)	0.0250 (5)	
C15	0.0259 (4)	0.7809 (2)	0.7396 (2)	0.0283 (6)	
C16	−0.1390 (4)	0.6607 (3)	0.8655 (3)	0.0341 (6)	
H1	0.182 (5)	0.754 (3)	0.497 (3)	0.050 (11)*	

### (II) (1,4,7,10-Tetraazacyclododecane-κ4N)(tricyanomethanido-κN)copper tricyanomethanide Atomic displacement parameters (Å^2^)

**Table:**

	*U*^11^	*U*^22^	*U*^33^	*U*^12^	*U*^13^	*U*^23^
Cu1	0.01993 (17)	0.01743 (17)	0.02207 (17)	−0.00544 (11)	0.00296 (11)	0.00163 (11)
N1	0.0346 (13)	0.0202 (11)	0.0210 (10)	−0.0094 (9)	−0.0035 (9)	0.0010 (8)
N2	0.0224 (11)	0.0236 (11)	0.0300 (12)	−0.0038 (9)	0.0000 (9)	−0.0002 (9)
N3	0.0267 (12)	0.0276 (12)	0.0233 (11)	−0.0044 (9)	0.0063 (9)	0.0034 (9)
N4	0.0241 (11)	0.0175 (11)	0.0287 (11)	−0.0041 (8)	0.0034 (9)	−0.0007 (9)
C1	0.0408 (17)	0.0241 (13)	0.0281 (14)	−0.0071 (12)	−0.0062 (12)	0.0073 (11)
C2	0.0348 (15)	0.0184 (12)	0.0327 (14)	−0.0066 (11)	−0.0027 (11)	0.0021 (10)
C3	0.0290 (15)	0.0337 (15)	0.0299 (14)	0.0005 (11)	0.0095 (11)	−0.0043 (11)
C4	0.0348 (16)	0.0322 (15)	0.0239 (13)	−0.0030 (12)	0.0045 (11)	−0.0043 (11)
C5	0.0316 (15)	0.0304 (15)	0.0292 (14)	0.0006 (11)	−0.0039 (11)	0.0057 (11)
C6	0.0213 (13)	0.0256 (13)	0.0354 (15)	−0.0022 (10)	−0.0009 (11)	0.0000 (11)
C7	0.0306 (15)	0.0320 (15)	0.0296 (14)	−0.0062 (11)	0.0095 (11)	−0.0032 (11)
C8	0.0376 (16)	0.0313 (15)	0.0235 (13)	−0.0160 (12)	0.0068 (11)	0.0013 (11)
N5	0.0313 (13)	0.0243 (12)	0.0369 (13)	−0.0107 (10)	0.0020 (10)	0.0006 (10)
N6	0.0563 (19)	0.0349 (15)	0.0511 (17)	−0.0208 (13)	0.0016 (14)	−0.0070 (13)
N7	0.066 (2)	0.0471 (18)	0.0529 (18)	−0.0133 (15)	0.0308 (16)	0.0102 (14)
C9	0.0169 (12)	0.0253 (13)	0.0276 (13)	−0.0041 (10)	0.0028 (10)	−0.0001 (10)
C10	0.0309 (15)	0.0214 (13)	0.0344 (14)	−0.0099 (11)	0.0051 (11)	0.0008 (11)
C11	0.0303 (15)	0.0237 (14)	0.0432 (16)	−0.0110 (11)	0.0021 (12)	0.0051 (12)
C12	0.0390 (17)	0.0228 (14)	0.0495 (18)	−0.0085 (12)	0.0086 (14)	0.0048 (12)
N8	0.0275 (13)	0.0274 (13)	0.0476 (15)	−0.0024 (10)	−0.0018 (11)	−0.0020 (11)
N9	0.0374 (15)	0.0264 (13)	0.0558 (17)	−0.0061 (11)	0.0052 (12)	0.0008 (11)
N10	0.0554 (19)	0.0436 (17)	0.0609 (19)	−0.0139 (14)	0.0266 (16)	−0.0067 (14)
C13	0.0276 (14)	0.0237 (13)	0.0275 (13)	−0.0063 (10)	0.0004 (11)	−0.0015 (10)
C14	0.0237 (13)	0.0263 (14)	0.0265 (13)	−0.0079 (11)	−0.0039 (10)	0.0013 (10)
C15	0.0233 (13)	0.0270 (14)	0.0345 (14)	−0.0044 (11)	0.0022 (11)	−0.0066 (11)
C16	0.0372 (17)	0.0253 (14)	0.0406 (16)	−0.0084 (12)	0.0040 (13)	−0.0056 (12)

### (II) (1,4,7,10-Tetraazacyclododecane-κ4N)(tricyanomethanido-κN)copper tricyanomethanide Geometric parameters (Å, º)

**Table:**

Cu1—N2	2.014 (2)	C4—H4A	0.9900
Cu1—N3	2.022 (2)	C4—H4B	0.9900
Cu1—N4	2.029 (2)	C5—C6	1.504 (4)
Cu1—N1	2.034 (2)	C5—H5A	0.9900
Cu1—N5	2.097 (2)	C5—H5B	0.9900
N1—C8	1.477 (4)	C6—H6A	0.9900
N1—C1	1.486 (4)	C6—H6B	0.9900
N1—H1	0.87 (2)	C7—C8	1.513 (4)
N2—C3	1.484 (3)	C7—H7A	0.9900
N2—C2	1.485 (3)	C7—H7B	0.9900
N2—H2	0.94 (2)	C8—H8A	0.9900
N3—C4	1.483 (4)	C8—H8B	0.9900
N3—C5	1.483 (4)	N5—C9	1.157 (3)
N3—H3	0.92 (2)	N6—C11	1.148 (4)
N4—C7	1.478 (3)	N7—C12	1.152 (4)
N4—C6	1.484 (4)	C9—C10	1.393 (4)
N4—H4	0.84 (3)	C10—C12	1.399 (4)
C1—C2	1.514 (4)	C10—C11	1.411 (4)
C1—H1A	0.9900	N8—C14	1.145 (4)
C1—H1B	0.9900	N9—C15	1.147 (4)
C2—H2A	0.9900	N10—C16	1.152 (4)
C2—H2B	0.9900	C13—C16	1.399 (4)
C3—C4	1.515 (4)	C13—C15	1.413 (4)
C3—H3A	0.9900	C13—C14	1.416 (4)
C3—H3B	0.9900		
			
N2—Cu1—N3	85.61 (9)	C4—C3—H3A	109.9
N2—Cu1—N4	148.42 (9)	N2—C3—H3B	109.9
N3—Cu1—N4	85.57 (9)	C4—C3—H3B	109.9
N2—Cu1—N1	86.11 (9)	H3A—C3—H3B	108.3
N3—Cu1—N1	148.55 (9)	N3—C4—C3	107.7 (2)
N4—Cu1—N1	85.79 (9)	N3—C4—H4A	110.2
N2—Cu1—N5	107.9 (1)	C3—C4—H4A	110.2
N3—Cu1—N5	101.87 (9)	N3—C4—H4B	110.2
N4—Cu1—N5	103.57 (9)	C3—C4—H4B	110.2
N1—Cu1—N5	109.54 (9)	H4A—C4—H4B	108.5
C8—N1—C1	115.0 (2)	N3—C5—C6	109.0 (2)
C8—N1—Cu1	104.5 (2)	N3—C5—H5A	109.9
C1—N1—Cu1	107.4 (2)	C6—C5—H5A	109.9
C8—N1—H1	106 (3)	N3—C5—H5B	109.9
C1—N1—H1	114 (3)	C6—C5—H5B	109.9
Cu1—N1—H1	109 (3)	H5A—C5—H5B	108.3
C3—N2—C2	114.3 (2)	N4—C6—C5	107.4 (2)
C3—N2—Cu1	109.0 (2)	N4—C6—H6A	110.2
C2—N2—Cu1	102.7 (2)	C5—C6—H6A	110.2
C3—N2—H2	106 (2)	N4—C6—H6B	110.2
C2—N2—H2	109 (2)	C5—C6—H6B	110.2
Cu1—N2—H2	116 (2)	H6A—C6—H6B	108.5
C4—N3—C5	114.9 (2)	N4—C7—C8	109.2 (2)
C4—N3—Cu1	104.9 (2)	N4—C7—H7A	109.8
C5—N3—Cu1	108.2 (2)	C8—C7—H7A	109.8
C4—N3—H3	100 (3)	N4—C7—H7B	109.8
C5—N3—H3	117 (3)	C8—C7—H7B	109.8
Cu1—N3—H3	111 (3)	H7A—C7—H7B	108.3
C7—N4—C6	115.8 (2)	N1—C8—C7	109.2 (2)
C7—N4—Cu1	108.7 (2)	N1—C8—H8A	109.8
C6—N4—Cu1	102.8 (2)	C7—C8—H8A	109.8
C7—N4—H4	106 (2)	N1—C8—H8B	109.8
C6—N4—H4	111 (2)	C7—C8—H8B	109.8
Cu1—N4—H4	112 (2)	H8A—C8—H8B	108.3
N1—C1—C2	109.4 (2)	C9—N5—Cu1	158.7 (2)
N1—C1—H1A	109.8	N5—C9—C10	178.4 (3)
C2—C1—H1A	109.8	C9—C10—C12	118.8 (3)
N1—C1—H1B	109.8	C9—C10—C11	119.6 (2)
C2—C1—H1B	109.8	C12—C10—C11	121.4 (2)
H1A—C1—H1B	108.2	N6—C11—C10	179.4 (4)
N2—C2—C1	107.4 (2)	N7—C12—C10	177.6 (3)
N2—C2—H2A	110.2	C16—C13—C15	122.2 (3)
C1—C2—H2A	110.2	C16—C13—C14	118.5 (2)
N2—C2—H2B	110.2	C15—C13—C14	119.3 (2)
C1—C2—H2B	110.2	N8—C14—C13	177.6 (3)
H2A—C2—H2B	108.5	N9—C15—C13	178.8 (3)
N2—C3—C4	108.8 (2)	N10—C16—C13	177.6 (4)
N2—C3—H3A	109.9		
			
C8—N1—C1—C2	89.8 (3)	C4—N3—C5—C6	89.1 (3)
Cu1—N1—C1—C2	−26.1 (3)	Cu1—N3—C5—C6	−27.7 (3)
C3—N2—C2—C1	−170.3 (2)	C7—N4—C6—C5	−170.4 (2)
Cu1—N2—C2—C1	−52.5 (2)	Cu1—N4—C6—C5	−52.0 (2)
N1—C1—C2—N2	53.8 (3)	N3—C5—C6—N4	54.4 (3)
C2—N2—C3—C4	84.5 (3)	C6—N4—C7—C8	86.1 (3)
Cu1—N2—C3—C4	−29.6 (3)	Cu1—N4—C7—C8	−29.0 (3)
C5—N3—C4—C3	−166.7 (2)	C1—N1—C8—C7	−164.2 (2)
Cu1—N3—C4—C3	−48.1 (2)	Cu1—N1—C8—C7	−46.6 (2)
N2—C3—C4—N3	52.5 (3)	N4—C7—C8—N1	51.6 (3)

### (II) (1,4,7,10-Tetraazacyclododecane-κ4N)(tricyanomethanido-κN)copper tricyanomethanide Hydrogen-bond geometry (Å, º)

**Table:**

*D*—H···*A*	*D*—H	H···*A*	*D*···*A*	*D*—H···*A*
N1—H1···N9	0.87 (2)	2.79 (3)	3.525 (4)	143 (3)
C1—H1*B*···N6^i^	0.99	2.54	3.273 (4)	131
N1—H1···N6^i^	0.87 (2)	2.60 (3)	3.206 (4)	127 (3)
N4—H4···N9^ii^	0.84 (3)	2.24 (4)	3.067 (3)	168 (3)
N3—H3···N7^iii^	0.92 (2)	2.05 (2)	2.928 (3)	159 (4)
N2—H2···N8^iv^	0.94 (2)	2.19 (2)	3.003 (3)	144 (3)
C3—H3*B*···N10^v^	0.99	2.64	3.531 (4)	150
C8—H8*B*···N8^vi^	0.99	2.56	3.538 (4)	171
C3—H3*A*···N10^vi^	0.99	2.64	3.460 (4)	140

Symmetry codes: (i) −*x*+1, −*y*+2, −*z*+1; (ii) −*x*, −*y*+2, −*z*+1; (iii) −*x*+1, −*y*+2, −*z*; (iv) −*x*+1, −*y*+1, −*z*+1; (v) *x*+1, *y*, *z*−1; (vi) −*x*, −*y*+1, −*z*+1.
