# Supplementary material for: Crystal structures of (1,4,7,10-tetra­aza­cyclo­dodecane-κ4 N)bis­(tri­cyano­methanido-κN)nickel and (1,4,7,10-tetra­aza­cyclo­dodecane-κ4 N)(tri­cyano­methanido-κN)copper tri­cyano­methanide
Source: Acta Crystallogr E Crystallogr Commun. 2015 May 23;71(Pt 6):693–7. doi: 10.1107/S2056989015009524 (PMC4459349; doi:10.1107/S2056989015009524)

Search Overview

Search:	search1
Date/Time done:	Mon May 04 10:12:16 2015
Database(s):	CSD version 5.29 (November 2007)<br>CSD version 5.35 updates (Feb 2014)<br>CSD version 5.34 updates (Nov 2012)<br>CSD version 5.34 updates (Feb 2013)<br>CSD version 5.32 updates (Feb 2011)<br>CSD version 5.31 updates (Nov 2009)<br>CSD version 5.31 updates (Feb 2010)<br>CSD version 5.31 updates (May 2010)<br>CSD version 5.31 updates (Aug 2010)<br>CSD version 5.30 updates (Nov 2008)<br>CSD version 5.30 updates (Feb 2009)<br>CSD version 5.30 updates (May 2009)<br>CSD version 5.30 updates (Sep 2009)<br>CSD version 5.29 updates (Jan 2008)<br>CSD version 5.29 updates (Aug 2008)<br>CSD version 5.33 updates (Feb 2012)<br>CSD version 5.33 updates (May 2012)<br>CSD version 5.33 updates (Aug 2012)
Restriction Info:	No refcode restrictions applied
Filters:	None
Percentage Completed:	100%
Number of Hits:	121

Single query used. Search found structures that:

match
Query 1

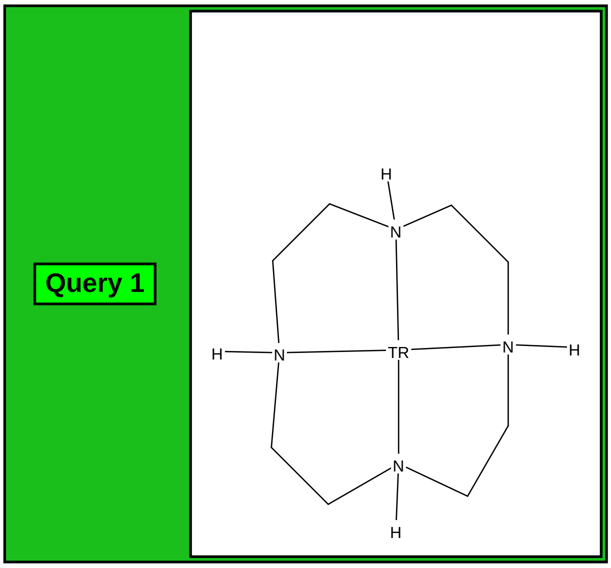

Search search1 (Mon May 04 10:12:16 2015): Hits 1-4

AZCDCO

Reference: Y.Iitaka, M.Shina, E.Kimura (1974) *Inorg.Chem.,* **13,** 2886

Formula: $C_8\ H_{20}\ Co_1\ N_6\ O_4\ ^{1+},Cl_1\ ^{1-},H_2\ O_1$

Compound Name: Dinitro-(1,4,7,10-tetra-azacyclododecane)-cobalt(iii) chloride monohydrate

Space Group:	P21	***Cell:***	***a*** 7.627(5)	***b*** 13.003(10)	***c*** 7.616(5)		
Space Group No.:	4	***(Å,°)***	α 90.00	β 102.75(10)	γ 90.00		
R-Factor (%):	2.6	***Temperature(K):***	295	***Density(g/cm³):***	1.698		

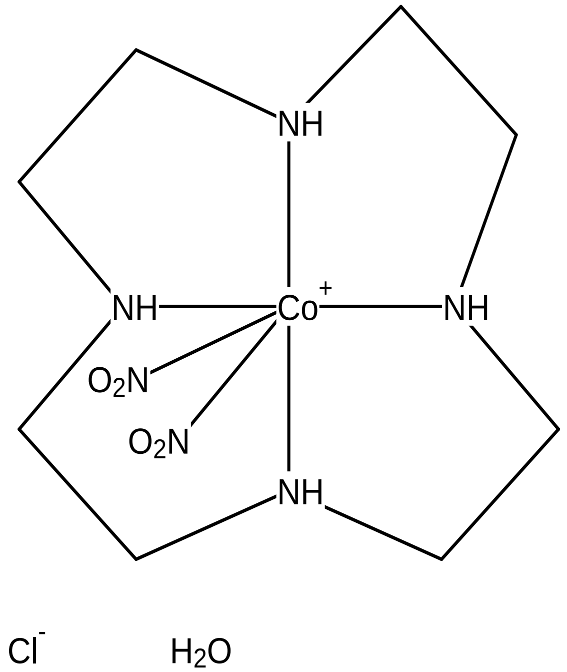

BAXHOX

Reference: T.Sakurai, K.Kobayashi, A.Hasegawa, S.Tsuboyama, K.Tsuboyama (1982) *Acta Crystallogr.,Sect.B,* **38,** 107

Formula: $C_{16}\ H_{36}\ Cl_1\ Cu_1\ N_4\ ^{1+},Cl_1\ ^{1-},2.6(H_2\ O_1)$

Compound Name: Chloro-(2,5,8,11-tetraethyl-1,4,7,10-tetra-azacyclododecane)-copper(ii) chloride hydrate

Space Group:	P41212	***Cell:***	***a*** 15.564(2)	***b*** 15.564(2)	***c*** 9.843(1)		
Space Group No.:	92	***(Å,°)***	α 90.00	β 90.00	γ 90.00		
R-Factor (%):	5.5	***Temperature(K):***	295	***Density(g/cm³):***	1.298		

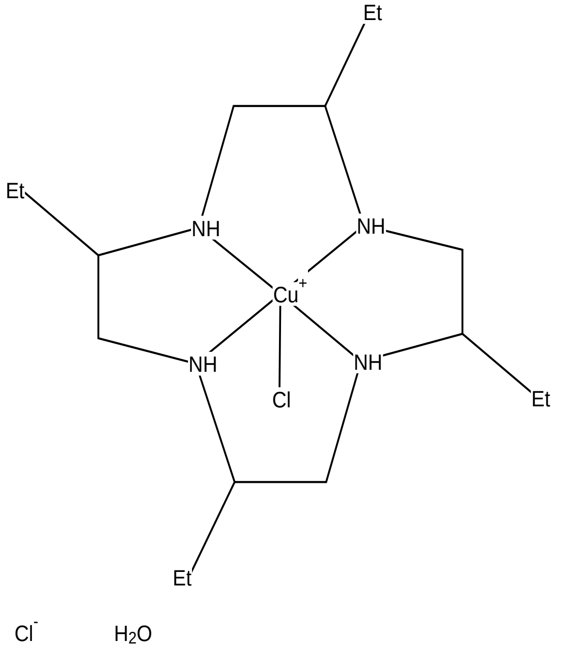

BREACO

Reference: T.Sakurai, S.Tsuboyama, K.Tsuboyama (1980) *Acta Crystallogr.,Sect.B,* **36,** 1797

Formula: $C_{16}\ H_{38}\ Br_1\ Co_1\ N_4\ O_1\ ^{2+},2(Br_1\ ^{1-})$

Compound Name: cis-(S,S,S,R)-Bromo-aqua-((2R,5R,8R,11R)-2,5,8,11-tetraethyl-1,4,7,10-tetra-azacyclododecane)-cobalt(iii) dibromide

Space Group:	P21	***Cell:***	***a*** 12.089(10)	***b*** 12.384(14)	***c*** 7.698(5)		
Space Group No.:	4	***(Å,°)***	α 90.00	β 95.59(7)	γ 90.00		
R-Factor (%):	4.2	***Temperature(K):***	295	***Density(g/cm³):***	1.741		

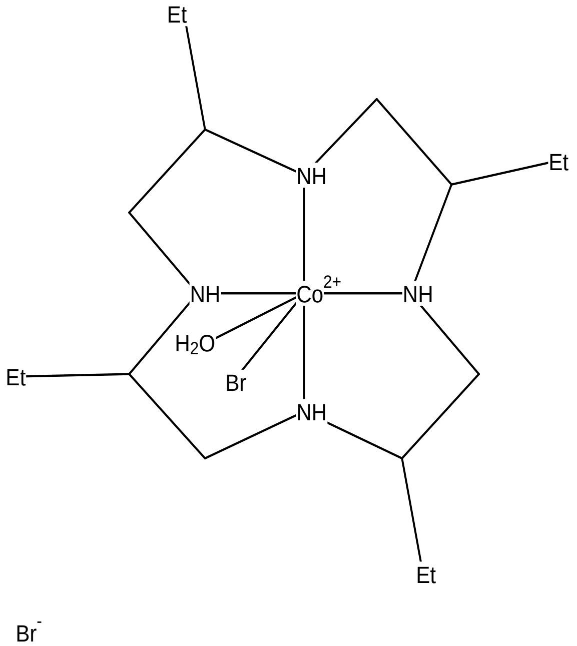

CALVER

Reference: V.C.Lau, L.A.Berben, J.R.Long (2002) *J.Am.Chem.Soc.,* **124,** 9042

Formula: $C_{48}\ H_{96}\ N_{24}\ Ru_4\ ^{9+},9(Cl_1\ ^{1-}),24(H_2\ O_1)$

Compound Name: tetrakis((μ_2-Pyrazine)-(1,4,7,10-tetraazacyclododecane)-ruthenium(ii)) nonachloride hydrate

Space Group:	Fddd	***Cell:***	***a*** 8.660(6)	***b*** 38.787(26)	***c*** 56.280(37)		
Space Group No.:	70	***(Å,°)***	α 90.00	β 90.00	γ 90.00		
R-Factor (%):	7.94	***Temperature(K):***	129	***Density(g/cm³):***	1.522		

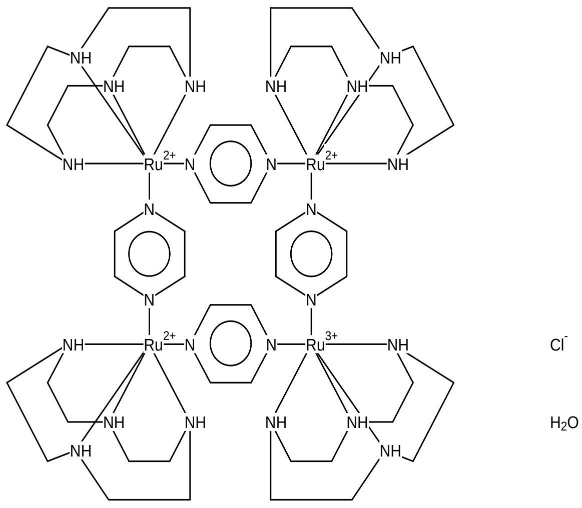

Search search1 (Mon May 04 10:12:16 2015): Hits 5-8

CATCEF

Reference: N.Matsumoto, A.Hirano, T.Hara, A.Ohyoshi (1983) *J.Chem.Soc.,Dalton Trans.*,2405

Formula: $C_{13}\,H_{27}\,Co_1\,N_4\,O_2\,^{2+}$,2($Cl_1\,O_4\,^{1-}$),$H_2\,O_1$

Compound Name: (Pentane-2,4-dionato)-(1,4,7,10-tetra-azacyclododecane)-cobalt(iii) diperchlorate monohydrate

Space Group:	Pna21	***Cell:***	***a***	18.974(6)	***b***	13.403(4)	***c***	8.928(2)
Space Group No.:	33	***(Å,°)***	α	90.00	β	90.00	γ	90.00

R-Factor (%): 3.99 ***Temperature(K):*** 295 ***Density(g/cm³):*** 1.601

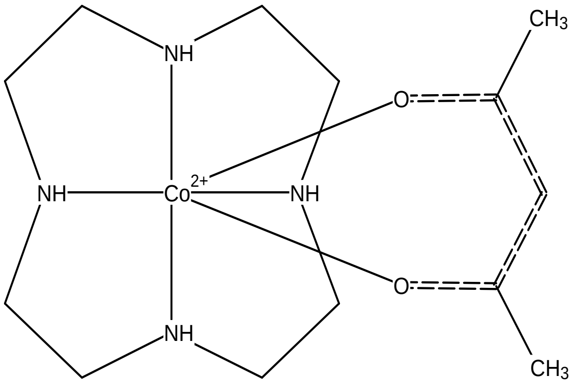

ClO_4^- H_2O

CATCIJ

Reference: N.Matsumoto, A.Hirano, T.Hara, A.Ohyoshi (1983) *J.Chem.Soc.,Dalton Trans.*,2405

Formula: $C_{13}\,H_{26}\,Br_1\,Co_1\,N_4\,O_2\,^{2+}$,2($Cl_1\,O_4\,^{1-}$),0.5($H_2\,O_1$)

Compound Name: (3-Bromopentane-2,4-dionato)-(1,4,7,10-tetra-azacyclododecane)-cobalt(iii) diperchlorate hemihydrate

Space Group:	Pbca	***Cell:***	***a***	15.138(4)	***b***	21.360(5)	***c***	14.066(7)
Space Group No.:	61	***(Å,°)***	α	90.00	β	90.00	γ	90.00

R-Factor (%): 5.5 ***Temperature(K):*** 295 ***Density(g/cm³):*** 1.802

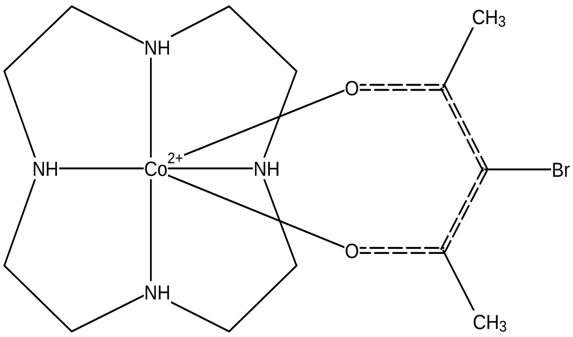

ClO_4^- H_2O

CEHBUN

Reference: Wai-Fun Yeung, Hoi-Ki Kwong, Tai-Chu Lau, Song Gao, Lap Szeto, Wing-Tak Wong (2006) *Polyhedron,***25,**1256

Formula: $C_{24}\,H_{40}\,N_{16}\,Ni_2\,Pt_2$,6($H_2\,O_1$)

Compound Name: tetrakis(μ_2-Cyano-C,N)-tetracyano-bis(1,4,7,10-tetra-azacyclododecane-N,N',N'',N''')-di-nickel(ii)-di-platinum hexahydrate

Space Group:	P21/c	***Cell:***	***a***	8.680(3)	***b***	14.218(3)	***c***	15.758(4)
Space Group No.:	14	***(Å,°)***	α	90.00	β	93.69(3)	γ	90.00

R-Factor (%): 2.35 ***Temperature(K):*** 301 ***Density(g/cm³):*** 1.999

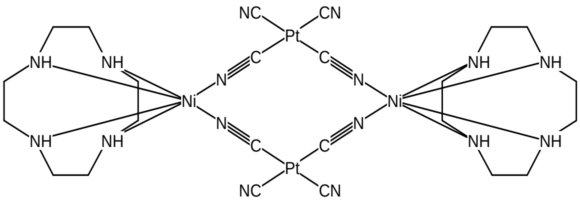

H_2O

CEHCAU

Reference: Wai-Fun Yeung, Hoi-Ki Kwong, Tai-Chu Lau, Song Gao, Lap Szeto, Wing-Tak Wong (2006) *Polyhedron,***25,**1256

Formula: $C_{24}\,H_{40}\,N_{16}\,Ni_4$,6($H_2\,O_1$)

Compound Name: tetrakis(μ_2-Cyano-C,N)-tetracyano-bis(1,4,7,10-tetra-azacyclododecane-N,N',N'',N''')-tetra-nickel(ii) hexahydrate

Space Group:	P21/c	***Cell:***	***a***	8.643(2)	***b***	14.218(4)	***c***	15.542(5)
Space Group No.:	14	***(Å,°)***	α	90.00	β	93.96(1)	γ	90.00

R-Factor (%): 2.62 ***Temperature(K):*** 298 ***Density(g/cm³):*** 1.561

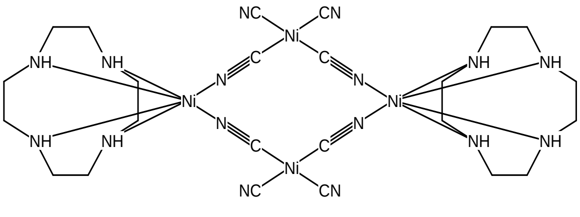

H_2O

CEHCEY

Reference: Wai-Fun Yeung, Hoi-Ki Kwong, Tai-Chu Lau, Song Gao, Lap Szeto, Wing-Tak Wong (2006) *Polyhedron,* **25,**1256

Formula: $C_{24}\ H_{40}\ Mn_2\ N_{16}\ Ni_2,6(H_2\ O_1)$

Compound Name: tetrakis(μ$_2$-Cyano-C,N)-tetracyano-bis(1,4,7,10-tetra-azacyclododecane-N,N',N'',N''')-di-manganese-di-nickel(ii) hexahydrate

Space Group:	P21/c	***Cell:***	***a*** 8.632(1)	***b*** 14.177(1)	***c*** 15.571(1)		
Space Group No.:	14	***(Å,°)***	α 90.00	β 93.75(1)	γ 90.00		
R-Factor (%):	4.7	***Temperature(K):***	298	***Density(g/cm³):***	1.551		

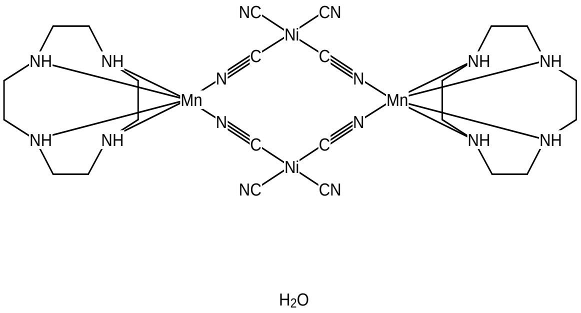

CELLUA

Reference: T.Yamaguchi, F.Yamazaki, T.Ito (1999) *J.Am.Chem.Soc.,* **121,**7405

Formula: $C_{30}\ H_{36}\ Cd_1\ N_6\ Pt_1\ ^{2+},2(Cl_1\ O_4\ ^{1-}),C_3\ H_6\ O_1$

Compound Name: (1,4,7,10-Tetra-azacyclododecane)-bis(2-(2-pyridyl)phenyl)-cadmium(ii)-platinum(ii) diperchlorate acetone solvate

Synonym: (Cyclen)-bis(2-(2-pyridyl)phenyl)-cadmium(ii)-platinum(ii) diperchlorate acetone solvate

Space Group:	P21/n	***Cell:***	***a*** 13.352(1)	***b*** 14.035(1)	***c*** 20.269(2)		
Space Group No.:	14	***(Å,°)***	α 90.00	β 106.60(0)	γ 90.00		
R-Factor (%):	5.06	***Temperature(K):***	213	***Density(g/cm³):***	1.907		

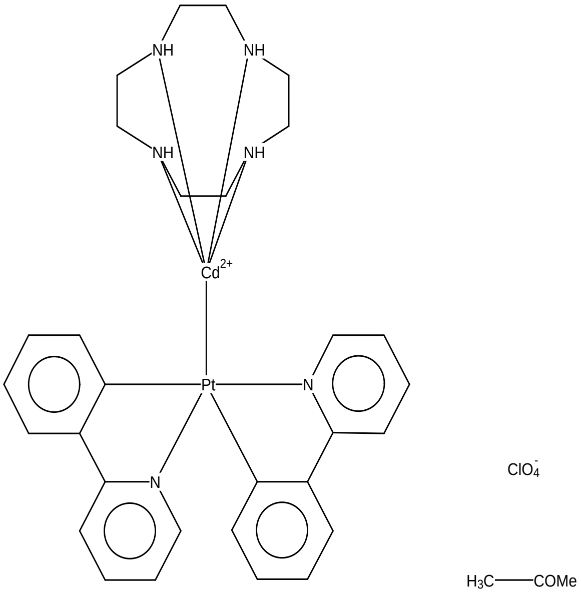

CELMAH

Reference: T.Yamaguchi, F.Yamazaki, T.Ito (1999) *J.Am.Chem.Soc.,* **121,**7405

Formula: $C_{20}\ H_{34}\ Cd_1\ N_6\ Pt_1\ ^{2+},2(Cl_1\ O_4\ ^{1-}),C_3\ H_6\ O_1$

Compound Name: (1,4,7,10-Tetra-azacyclododecane)-(2,2'-bipyridyl)-dimethyl-cadmium(ii)-platinum(ii) diperchlorate acetone solvate

Space Group:	C2	***Cell:***	***a*** 20.966(2)	***b*** 11.314(1)	***c*** 16.582(2)		
Space Group No.:	5	***(Å,°)***	α 90.00	β 120.94(0)	γ 90.00		
R-Factor (%):	3.73	***Temperature(K):***	295	***Density(g/cm³):***	1.817		

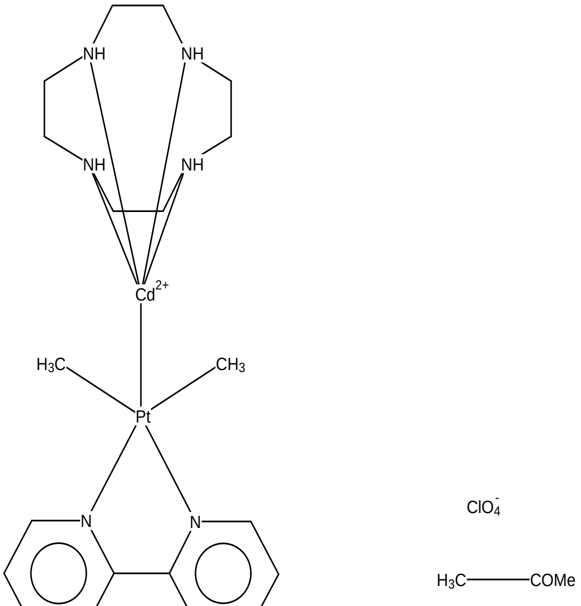

DOHXUT

Reference: M.Kojima, K.Nakabayashi, S.Ohba, S.Okumoto, Y.Saito, J.Fujita (1986) *Bull.Chem.Soc.Jpn.,* **59,**277

Formula: $C_{11}\ H_{28}\ Co_1\ N_5\ S_1\ ^{2+},Cl_4\ Zn_1\ ^{2-},H_2\ O_1$

Compound Name: ((S)-1-Amino-2-propanethiolato-N,S)-(1,4,7,10-tetra-azacyclododecane-N1,N4,N7,N10)-cobalt(iii) tetrachloro-zinc monohydrate

Space Group:	P21	***Cell:***	***a*** 9.382(2)	***b*** 13.124(3)	***c*** 8.934(2)		
Space Group No.:	4	***(Å,°)***	α 90.00	β 91.08(3)	γ 90.00		
R-Factor (%):	2.8	***Temperature(K):***	295	***Density(g/cm³):***	1.65		

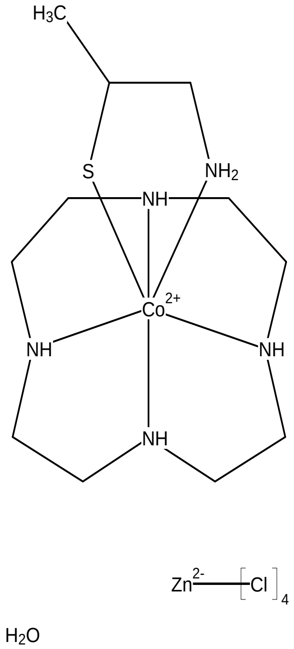

Search search1 (Mon May 04 10:12:16 2015): Hits 13-16

FABHEW

Reference: Tian-Huey Lu, K.Panneerselvam, Li-Hsuan Chen, Yung-Jan Lin, Fen-Ling Liao, Chung-Sun Chung (2001) *Anal.Sci.*,**17**, 571

Formula: $C_{18}\ H_{28}\ N_6\ Ni_1\ ^{2+},2(Cl_1\ O_4\ ^{1-})$

Compound Name: (2,2'-Bipyridyl)-(1,4,7,10-tetra-azacyclododecane)-nickel(ii) diperchlorate

Space Group:	Pbca	***Cell:***	***a*** 13.991(0)	***b*** 14.698(0)	***c*** 24.068(1)
Space Group No.:	61	***(Å,°)***	α 90.00	β 90.00	γ 90.00

R-Factor (%): 5.7 ***Temperature(K):*** 296 ***Density(g/cm³):*** 1.573

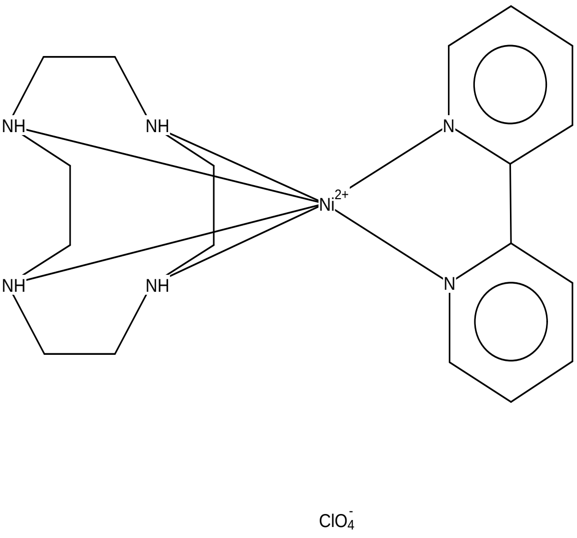

FAQBIJ

Reference: S.L.Heath, R.H.Laye, C.A.Muryn, N.Lima, R.Sessoli, R.Shaw, S.J.Teat, G.A.Timco, R.E.P.Winpenny (2004) *Angew.Chem.,Int.Ed.Engl.*,**43**,6132

Formula: $C_{136}\ H_{258}\ Cr_{12}\ F_{16}\ N_8\ Ni_3\ O_{50},5.5(C_4\ H_8\ O_1),1.5(C_2\ H_3\ N_1)$

Compound Name: hexadecakis(μ_2-Fluoro)-tetracosakis(μ_2-pivalato)-dihydroxy-bis(1,4,7,10-tetraazacyclododecane)-dodeca-chromium(iii)-tri-nickel(ii) tetrahydrofuran acetonitrile solvate

Space Group:	P-1	***Cell:***	***a*** 15.978(3)	***b*** 16.249(2)	***c*** 23.260(4)
Space Group No.:	2	***(Å,°)***	α 79.72(1)	β 72.17(1)	γ 84.78(1)

R-Factor (%): 11.06 ***Temperature(K):*** 100 ***Density(g/cm³):*** 1.283

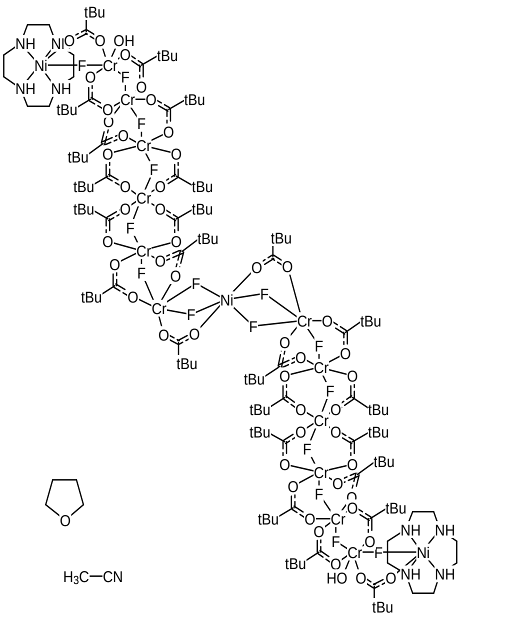

FATZUV

Reference: D.J.Hodgson, E.Pedersen, H.Toftlund, C.Weiss (1986) *Inorg.Chim.Acta*,**120**,177

Formula: $C_{16}\ H_{42}\ Cr_2\ N_8\ O_2\ ^{4+},2(O_6\ S_2\ ^{2-}),4(H_2\ O_1)$

Compound Name: bis(μ_2-Hydroxo)-bis(1,4,7,10-tetra-azacyclododecane-N,N',N'',N''')-di-chromium(iii) bis(dithionate) tetrahydrate

Space Group:	P21/n	***Cell:***	***a*** 8.837(5)	***b*** 14.472(8)	***c*** 13.943(6)
Space Group No.:	14	***(Å,°)***	α 90.00	β 95.83(4)	γ 90.00

R-Factor (%): 5.9 ***Temperature(K):*** 295 ***Density(g/cm³):*** 1.638

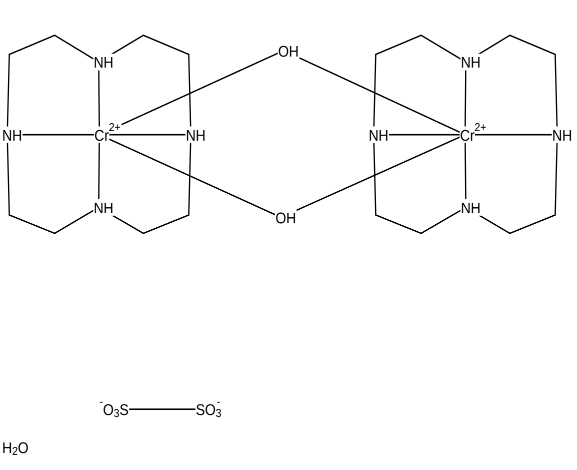

FEVWIM

Reference: S.Tsuboyama, T.Sakurai, K.Tsuboyama (1987) *J.Chem.Soc.,Dalton Trans.*,721

Formula: $C_{19}\ H_{42}\ Co_1\ N_5\ O_2\ ^{2+},Br_1\ ^{1-},Cl_1\ O_4\ ^{1-},H_2\ O_1$

Compound Name: cis-β-SSSR-((R)-Alaninato-N,O)-((2R,5R,8R,11R)-2,5,8,11-tetraethyl-1,4,7,10-tetra-azacyclodecane-N,N',N'',N''')-cobalt(iii) bromide perchlorate monohydrate

Space Group:	P43	***Cell:***	***a*** 14.320(6)	***b*** 14.320(6)	***c*** 13.377(2)
Space Group No.:	78	***(Å,°)***	α 90.00	β 90.00	γ 90.00

R-Factor (%): 4.4 ***Temperature(K):*** 295 ***Density(g/cm³):*** 1.523

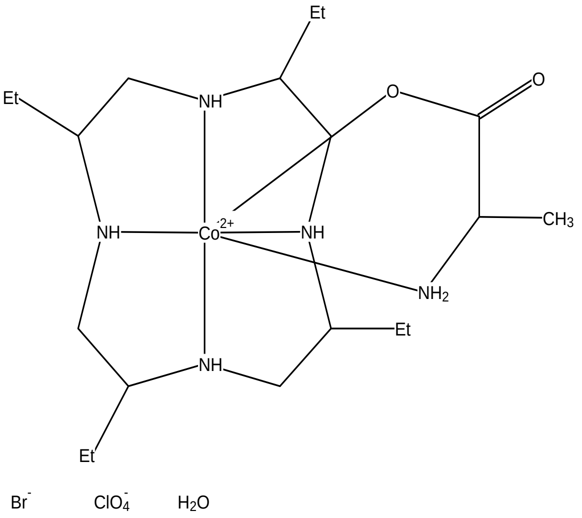

Search search1 (Mon May 04 10:12:16 2015): Hits 17-20

FEVWOS

Reference: S.Tsuboyama, T.Sakurai, K.Tsuboyama (1987) *J.Chem.Soc.,Dalton Trans.*,721

Formula: $C_{19}\,H_{42}\,Co_1\,N_5\,O_2\,^{2+}$,$Br_1\,^{1-}$,$Cl_1\,O_4\,^{1-}$,$H_2\,O_1$

Compound Name: cis-β-SSSR-((S)-Alaninato-N,O)-((2R,5R,8R,11R)-2,5,8,11-tetraethyl-1,4,7,10-tetra-azacyclodecane-N,N',N'',N''')-cobalt(iii) bromide perchlorate monohydrate

Space Group:	P43	***Cell:***	***a*** 14.319(3)	***b*** 14.319(3)	***c*** 13.325(7)
Space Group No.:	78	***(Å,°)***	α 90.00	β 90.00	γ 90.00

R-Factor (%): 4.2 ***Temperature(K):*** 295 ***Density(g/cm³):*** 1.529

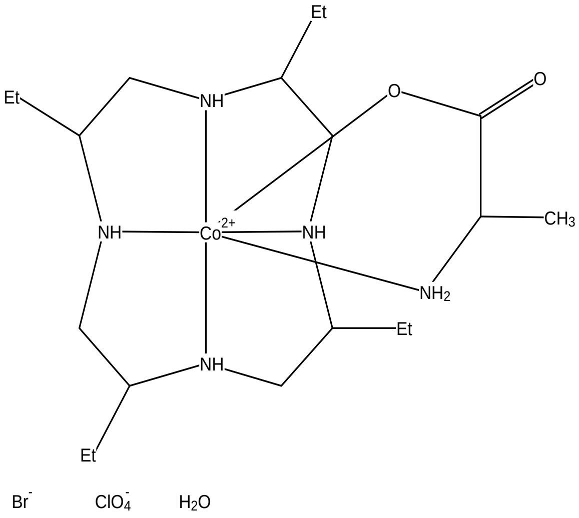

GAGVAM

Reference: In Su Lee, J.R.Long (2004) *Discussion of Faraday Soc.*, 3434

Formula: $C_{10}\,H_{20}\,F_6\,N_4\,O_6\,S_2\,V_1\,^{1+}$,$C_1\,F_3\,O_3\,S_1\,^{1-}$

Compound Name: (1,4,7,10-Tetra-azacyclododecane-N,N',N'',N''')-bis(trifluoromethanesulfonato)-vanadium(iii) trifluoromethanesulfonate

Space Group:	Pna21	***Cell:***	***a*** 8.713(0)	***b*** 22.149(2)	***c*** 12.289(1)
Space Group No.:	33	***(Å,°)***	α 90.00	β 90.00	γ 90.00

R-Factor (%): 4.91 ***Temperature(K):*** 139 ***Density(g/cm³):*** 1.878

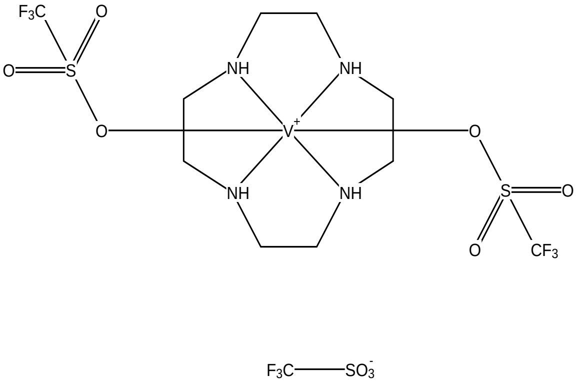

GAGVEQ

Reference: In Su Lee, J.R.Long (2004) *Discussion of Faraday Soc.*, 3434

Formula: $C_{11}\,H_{20}\,N_7\,V_1$

Compound Name: Tricyano-(1,4,7,10-tetra-azacyclododecane-N,N',N'',N''')-vanadium(iii)

Space Group:	Pnma	***Cell:***	***a*** 13.633(1)	***b*** 12.259(0)	***c*** 8.245(0)
Space Group No.:	62	***(Å,°)***	α 90.00	β 90.00	γ 90.00

R-Factor (%): 6.68 ***Temperature(K):*** 147 ***Density(g/cm³):*** 1.452

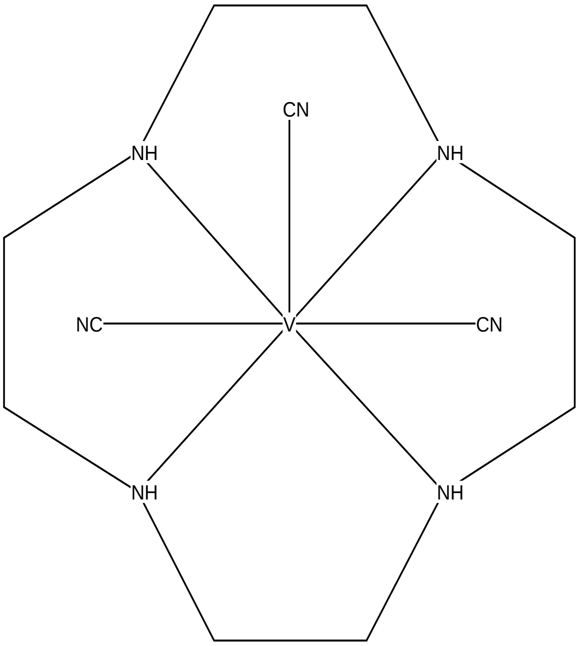

GAGVIU

Reference: In Su Lee, J.R.Long (2004) *Discussion of Faraday Soc.*, 3434

Formula: $C_{38}\,H_{80}\,N_{22}\,V_4\,^{6+}$,6($C_1\,F_3\,O_3\,S_1\,^{1-}$),1.25($C_1\,H_2\,Cl_2$),$C_1\,H_3\,N_1\,O_2$

Compound Name: hexakis(μ2-Cyano-C,N)-tetrakis(1,4,7,10-tetra-azacyclododecane-N,N',N'',N''')-tetra-vanadium(iii) hexakis(trifluoromethanesulfonate) dichloromethane nitromethane solvate

Space Group:	Pnma	***Cell:***	***a*** 30.109(4)	***b*** 22.106(4)	***c*** 13.600(1)
Space Group No.:	62	***(Å,°)***	α 90.00	β 90.00	γ 90.00

R-Factor (%): 11.55 ***Temperature(K):*** 147 ***Density(g/cm³):*** 1.549

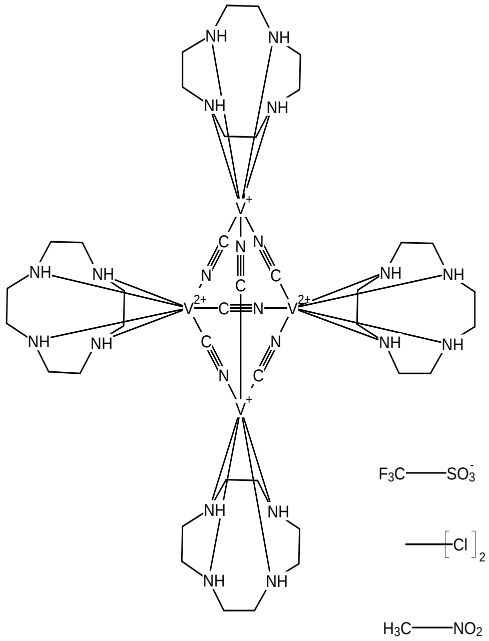

Search search1 (Mon May 04 10:12:16 2015): Hits 21-24

GALYUN

Reference: S.Tsuboyama, T.Takishima, T.Sakurai, K.Tsuboyama (1987) *Nippon Kagaku Kaishi(J.Chem.Soc.Jpn.),*313

Formula: $C_{20} H_{41} Co_1 N_5 O_4 {}^{1+},Cl_1 O_4 {}^{1-},4(H_2 O_1)$

Compound Name: (+)-(α-Amino-α-methylmalonato-N,O)-(2R,5R,8R,11R-2,5,8,11-tetraethyl-1,4,7,10-tetra-azacyclododecane)-cobalt(iii) perchlorate tetrahydrate

Space Group: P21 ***Cell:*** ***a*** 13.985(6) ***b*** 11.768(13) ***c*** 9.626(3)
Space Group No.: 4 ***(Å,°)*** α 90.00 β 108.36(3) γ 90.00

R-Factor (%): 5.5 ***Temperature(K):*** 295 ***Density(g/cm³):*** 1.426

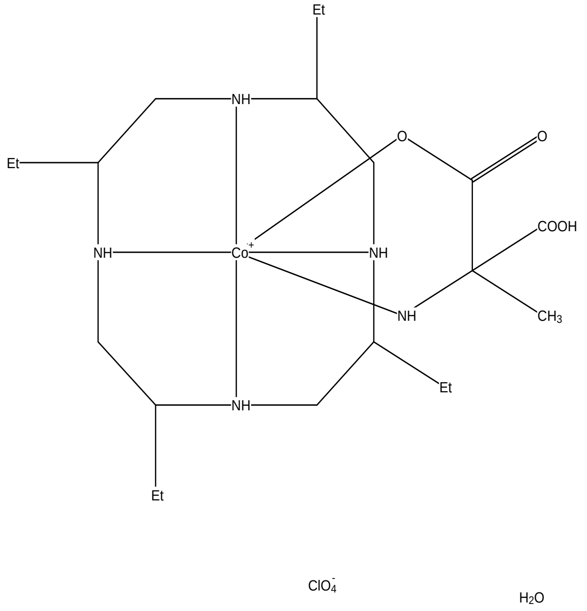

GEVTUW

Reference: S.J.Carrington, D.A.Buckingham, J.Simpson, A.G.Blackman, C.R.Clark (1999) *J.Chem.Soc.,Dalton Trans.,*3809

Formula: $C_8 H_{23} Co_1 N_4 O_4 P_1 {}^{1+},Cl_1 O_4 {}^{1-},3(H_2 O_1)$

Compound Name: cis-anti-Aqua-syn-(phosphito-O)-(1,4,7,10-tetra-azacyclododecane-N,N',N'',N''')-cobalt(iii) perchlorate trihydrate

Space Group: P-1 ***Cell:*** ***a*** 7.715(3) ***b*** 9.047(3) ***c*** 13.478(3)
Space Group No.: 2 ***(Å,°)*** α 78.51(2) β 83.63(2) γ 81.03(3)

R-Factor (%): 3.85 ***Temperature(K):*** 158 ***Density(g/cm³):*** 1.766

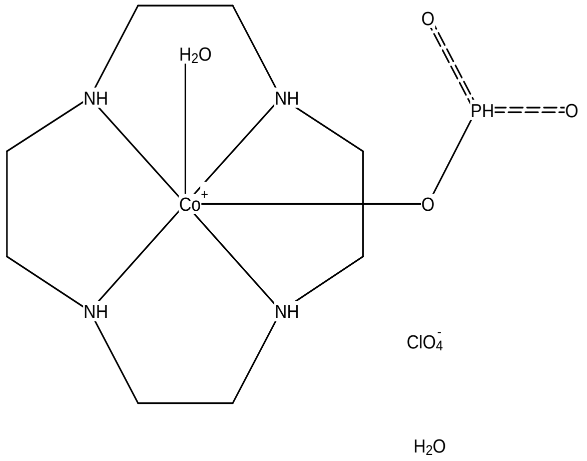

GUVFUY

Reference: Min Su Han, D.H.Kim (2003) *Supramolecular Chemistry,* **15,**59

Formula: $C_{14} H_{23} N_8 O_2 Zn_1 {}^{1+},Cl_1 O_4 {}^{1-}$

Compound Name: (1,4,7,10-Tetraazacyclododecane)-(lumazinato-N)-zinc(ii) perchlorate

Synonym: (Cyclen)-(lumazinato-N)-zinc(ii) perchlorate

Space Group: P-1 ***Cell:*** ***a*** 11.684(0) ***b*** 11.853(0) ***c*** 14.382(0)
Space Group No.: 2 ***(Å,°)*** α 97.25(0) β 92.57(0) γ 94.75(0)

R-Factor (%): 8.69 ***Temperature(K):*** 243 ***Density(g/cm³):*** 1.69

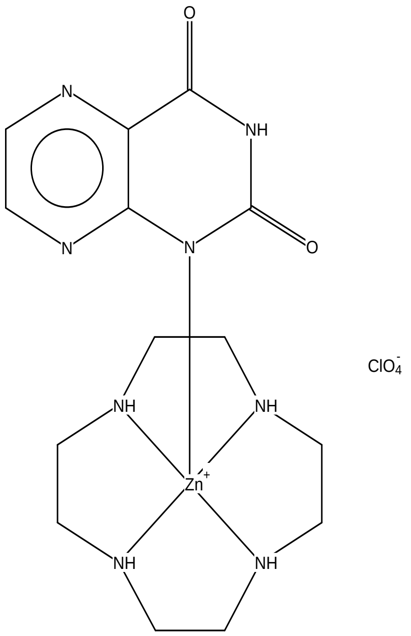

HILTOL

Reference: D.A.Buckingham, C.R.Clark, A.J.Rogers, J.Simpson (1998) *Inorg.Chem.,***37,**3497

Formula: $2(C_8 H_{23} Co_1 N_8 {}^{2+}),3(Cl_1 O_4 {}^{1-}),Cl_1 {}^{1-},1.5(H_2 O_1)$

Compound Name: bis((1,4,7,10-Tetra-azacyclodecane)-syn-ammonio-anti-azido-cobalt(iii)) chloride triperchlorate sesquihydrate

Space Group: P21/n ***Cell:*** ***a*** 9.008(6) ***b*** 28.690(15) ***c*** 14.528(7)
Space Group No.: 14 ***(Å,°)*** α 90.00 β 104.12(7) γ 90.00

R-Factor (%): 6.95 ***Temperature(K):*** 158 ***Density(g/cm³):*** 1.717

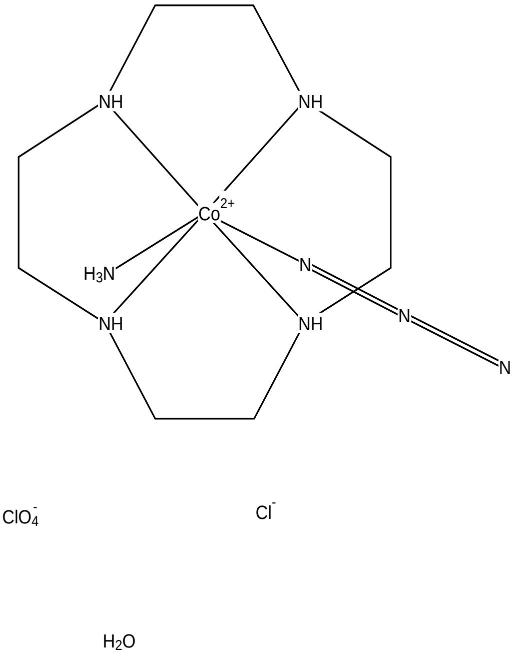

Search search1 (Mon May 04 10:12:16 2015): Hits 25-28

ICUDIU

Reference: J.Notni, H.Gorls, E.Anders (2006) *Eur.J.Inorg.Chem.*, 1444

Formula: $C_{15}\ H_{27}\ N_4\ S_1\ Zn_1\ ^{1+},Cl_1\ O_4\ ^{1-}$

Compound Name: ((+,+,+,+)-1,4,7,10-Tetra-azacyclododecane)-(phenylmethanethiolato)-zinc(ii) perchlorate

Space Group: P212121 ***Cell:*** ***a*** 8.672(0) ***b*** 14.610(0) ***c*** 15.803(0)
Space Group No.: 19 ***(Å,°)*** α 90.00 β 90.00 γ 90.00

R-Factor (%): 4.9 ***Temperature(K):*** 183 ***Density(g/cm³):*** 1.527

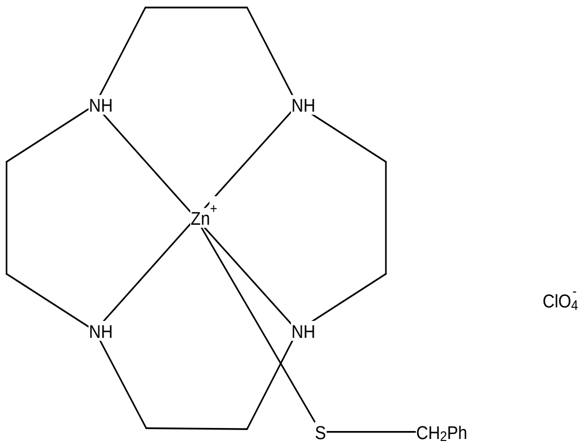

ICUDOA

Reference: J.Notni, H.Gorls, E.Anders (2006) *Eur.J.Inorg.Chem.*, 1444

Formula: $C_{15}\ H_{27}\ N_4\ S_1\ Zn_1\ ^{1+},Cl_1\ O_4\ ^{1-}$

Compound Name: ((+,+,+,+)-1,4,7,10-Tetra-azacyclododecane)-(4-methylbenzenethiolato)-zinc(ii) perchlorate

Space Group: P-1 ***Cell:*** ***a*** 8.152(0) ***b*** 10.811(0) ***c*** 11.195(0)
Space Group No.: 2 ***(Å,°)*** α 93.64(0) β 95.37(0) γ 90.27(0)

R-Factor (%): 4.1 ***Temperature(K):*** 183 ***Density(g/cm³):*** 1.559

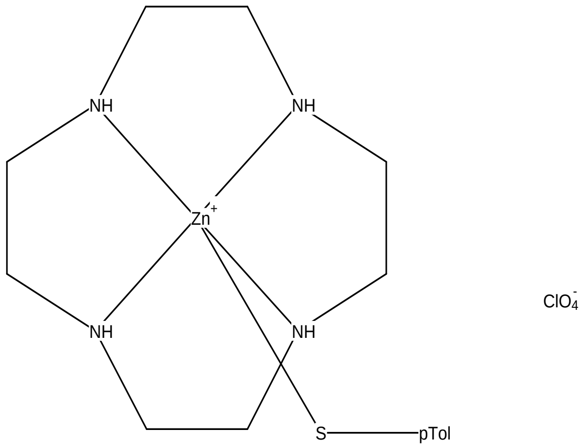

JAYKAV

Reference: S.Tsuboyama, S.Miki, T.Chijimatsu, K.Tsuboyama, T.Sakurai (1989) *J.Chem.Soc.,Dalton Trans.*,2359

Formula: $C_{19}\ H_{42}\ Co_1\ N_5\ O_3\ ^{2+},Br_1\ ^{1-},Cl_1\ O_4\ ^{1-}$

Compound Name: (R)-(Serinato-N,O)-((2R,5R,8R,11R)-2,5,8,11-tetraethyl-1,4,7,10-tetra-azacyclododecane-N,N',N'',N''')-cobalt(iii) bromide perchlorate

Space Group: P43 ***Cell:*** ***a*** 14.283(5) ***b*** 14.283(5) ***c*** 13.378(6)
Space Group No.: 78 ***(Å,°)*** α 90.00 β 90.00 γ 90.00

R-Factor (%): 9.4 ***Temperature(K):*** 295 ***Density(g/cm³):*** 1.526

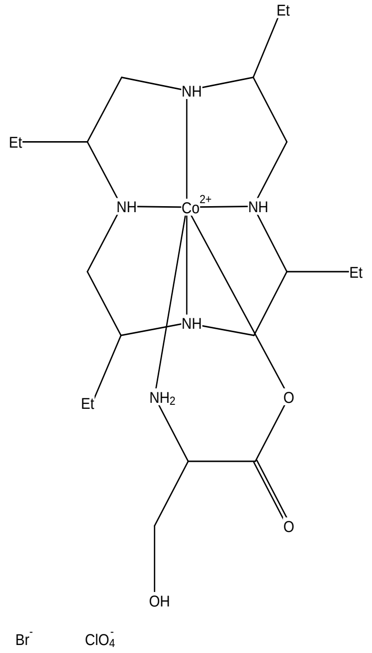

JAYKEZ

Reference: S.Tsuboyama, S.Miki, T.Chijimatsu, K.Tsuboyama, T.Sakurai (1989) *J.Chem.Soc.,Dalton Trans.*,2359

Formula: $C_{19}\ H_{42}\ Co_1\ N_5\ O_3\ ^{2+},Br_1\ ^{1-},Cl_1\ O_4\ ^{1-},H_2\ O_1$

Compound Name: (S)-(Serinato-N,O)-((2R,5R,8R,11R)-2,5,8,11-tetraethyl-1,4,7,10-tetra-azacyclododecane-N,N',N'',N''')-cobalt(iii) bromide perchlorate monohydrate

Space Group: P43 ***Cell:*** ***a*** 14.327(2) ***b*** 14.327(2) ***c*** 13.318(2)
Space Group No.: 78 ***(Å,°)*** α 90.00 β 90.00 γ 90.00

R-Factor (%): 7.3 ***Temperature(K):*** 295 ***Density(g/cm³):*** 1.567

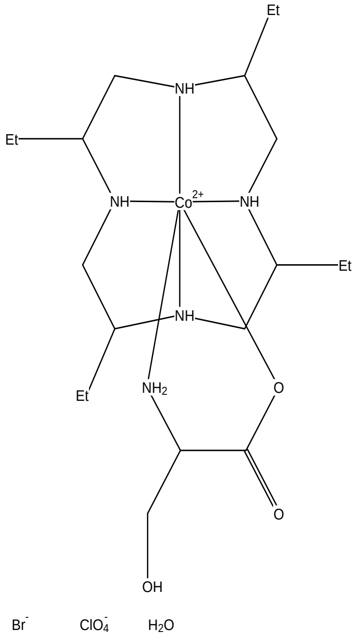

Search search1 (Mon May 04 10:12:16 2015): Hits 29-32

JAYKID

Reference: S.Tsuboyama, S.Miki, T.Chijimatsu, K.Tsuboyama, T.Sakurai (1989) *J.Chem.Soc.,Dalton Trans.*,2359

Formula: $C_{20} H_{44} Co_1 N_5 O_3$ $^{2+}$,Br_1 $^{1-}$,$Cl_1 O_4$ $^{1-}$

Compound Name: (R)-(α-Methylserinato-N,O)-((2R,5R,8R,11R)-2,5,8,11-tetraethyl-1,4,7,10-tetra-azacyclododecane-N,N',N'',N''')-cobalt(iii) bromide perchlorate

Space Group:	P43	***Cell:***	***a*** 14.168(4)	***b*** 14.168(4)	***c*** 13.834(6)
Space Group No.:	78	***(Å,°)***	α 90.00	β 90.00	γ 90.00

R-Factor (%): 6.0 ***Temperature(K):*** 295 ***Density(g/cm³):*** 1.533

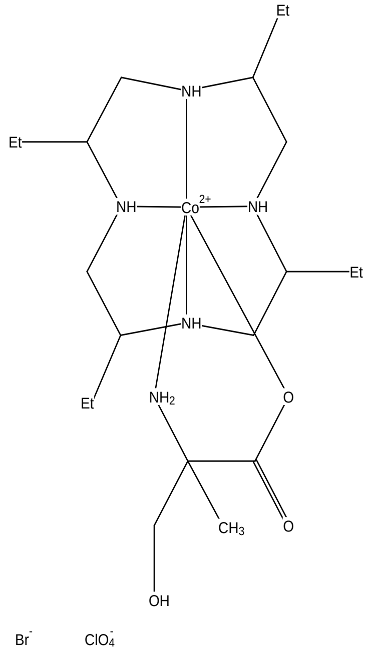

JIKJUI

Reference: B.Scott, K.J.Brewer, L.O.Spreer, C.A.Craig, J.W.Otvos, M.Calvin, S.Taylor (1990) *J.Coord.Chem.*,**21**,307

Formula: $C_8 H_{24} N_4 Ni_1 O_2$ $^{2+}$,2($Cl_1 O_4$ $^{1-}$),$H_2 O_1$

Compound Name: Diaqua-(1,4,7,10-tetra-azacyclodecane)-nickel diperchlorate monohydrate

Space Group:	P212121	***Cell:***	***a*** 11.173(5)	***b*** 11.976(5)	***c*** 13.969(4)
Space Group No.:	19	***(Å,°)***	α 90.00	β 90.00	γ 90.00

R-Factor (%): 5.3 ***Temperature(K):*** 295 ***Density(g/cm³):*** 1.72

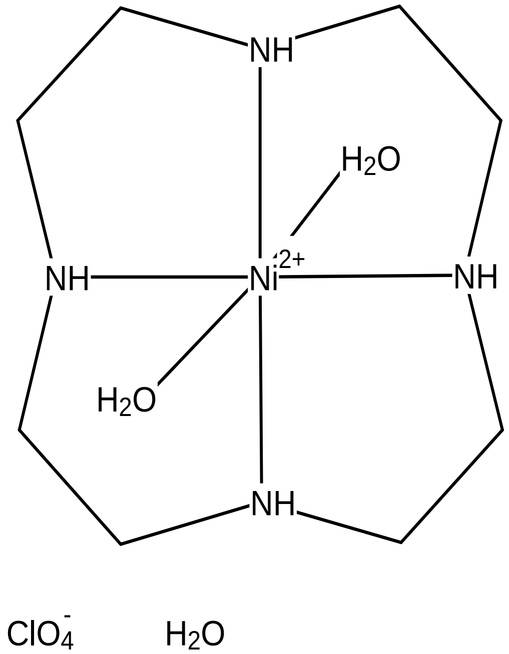

KAMBOP

Reference: S.Tsuboyama, K.Tsuboyama, T.Sakurai (1989) *Acta Crystallogr.,Sect.C(Cr.Str.Comm.),***45,**669

Formula: $C_{20} H_{41} Co_1 N_5 O_4$ $^{1+}$,$Cl_1 O_4$ $^{1-}$,2.5($H_2 O_1$)

Compound Name: cis-(SSSR)-β_1-((R)-2-Amino-2-methylmalonato-N,O)-(2R,5R,8R,11R-2,5,8,11-tetraethyl-1,4,7,10-tetra-azacyclododecane-N,N',N'',N''')-cobalt(iii) perchlorate hydrate

Space Group:	P1	***Cell:***	***a*** 10.455(5)	***b*** 31.715(15)	***c*** 10.492(7)
Space Group No.:	1	***(Å,°)***	α 110.50(5)	β 116.74(5)	γ 80.36(5)

R-Factor (%): 6.8 ***Temperature(K):*** 295 ***Density(g/cm³):*** 1.413

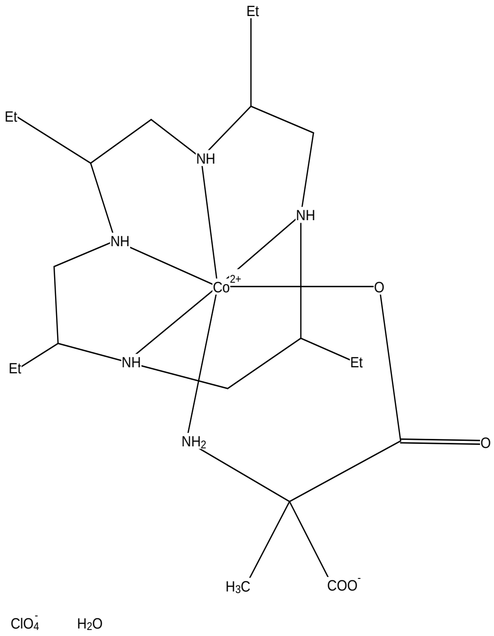

KEJDUZ

Reference: S.S.Massoud, F.A.Mautner, R.Vicente, B.M.Rodrigue (2006) *Inorg.Chim.Acta*,**359**,3321

Formula: $C_{24} H_{44} N_8 Ni_2 O_4$ $^{2+}$,2($Cl_1 O_4$ $^{1-}$)

Compound Name: (μ_2-Terephthalato-O,O',O'',O''')-bis((1,4,7,10-tetraazacyclododecane)-nickel(ii)) diperchlorate

Space Group:	Pnnm	***Cell:***	***a*** 10.908(2)	***b*** 15.985(3)	***c*** 9.246(2)
Space Group No.:	58	***(Å,°)***	α 90.00	β 90.00	γ 90.00

R-Factor (%): 4.19 ***Temperature(K):*** 100 ***Density(g/cm³):*** 1.699

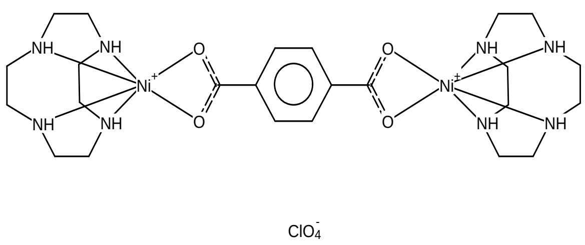

Search search1 (Mon May 04 10:12:16 2015): Hits 33-36

KEJMUH

Reference: A.Bencini, A.Bianchi, E.Garcia-Espana, Y.Jeannin, M.Julve, V.Marcelino, M.Philoche-Levisalles (1990) *Inorg.Chem.*,**29**, 963

Formula: $2(C_8 H_{24} N_4 Ni_1 O_2{}^{2+}),C_4 O_4{}^{2-},2(Cl_1 O_4{}^{1-})$

Compound Name: bis(Diaqua-(1,4,7,10-tetra-azacyclododecane-N,N',N'',N''')-nickel(ii)) squarate diperchlorate

Space Group:	P21cn	***Cell:***	***a*** 11.124(3)	***b*** 11.461(3)	***c*** 25.735(6)		
Space Group No.:	33	***(Å,°)***	α 90.00	β 90.00	γ 90.00		

R-Factor (%): 3.45 ***Temperature(K):*** 295 ***Density(g/cm³):*** 1.711

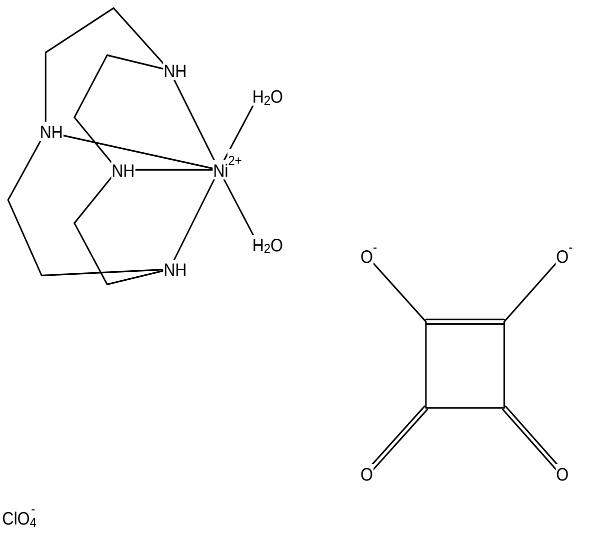

LEFGAE

Reference: A.H.Krotz, L.Y.Kuo, J.K.Barton (1993) *Inorg.Chem.*,**32**, 5963

Formula: $C_{22} H_{30} N_6 Rh_1{}^{3+},3(C_1 N_1 S_1{}^{1-}),2(H_2 O_1)$

Compound Name: (9,10-Phenanthrenequinone di-imine)-(1,4,7,10-tetra-azacyclododecane) -rhodium(iii) tris(thiocyanate) dihydrate

Space Group:	P-1	***Cell:***	***a*** 15.479(5)	***b*** 12.312(2)	***c*** 8.679(2)
Space Group No.:	2	***(Å,°)***	α 72.10(2)	β 83.98(2)	γ 71.52(2)

R-Factor (%): 4.2 ***Temperature(K):*** 295 ***Density(g/cm³):*** 1.539

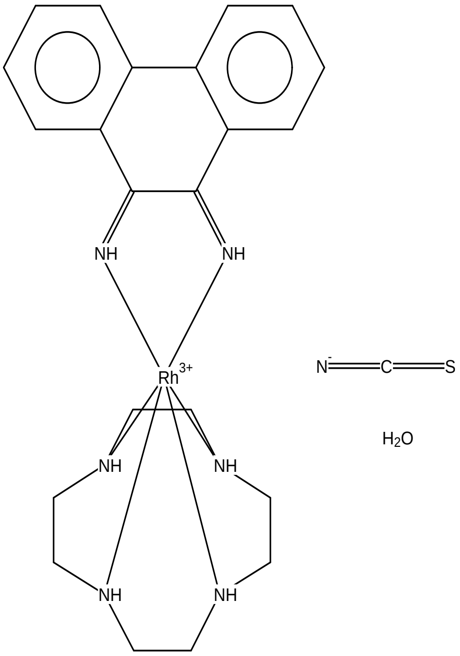

LEYWES

Reference: S.A.Baudron, M.W.Hosseini, N.Kyritsakas, M.Kurmoo (2007) *Discussion of Faraday Soc.*,1129

Formula: $C_{40} H_{52} N_{12} Ni_2 Pd_1 S_4{}^{2+},2(B_1 F_4{}^{1-}),6(C_3 H_7 N_1 O_1)$

Compound Name: bis(μ_2-5H-pyrido[3',2':4,5]cyclopenta[1,2-b]pyridin-5-ylidenemethanedithiolato-N,N',S,S')-bis(1,4,7,10-tetra-azacyclododecane) -palladium-di-nickel(ii) bis(tetrafluoroborate) dimethylformamide solvate

Synonym: bis(μ_2-4,5-diazafluoren-9-ylidenemethanedithiolato-N,N',S,S')-bis(1,4,7,10-tetra-azacyclododecane)-palladium-di-nickel(ii) bis(tetrafluoroborate) dimethylformamide solvate

Space Group:	P-1	***Cell:***	***a*** 8.479(0)	***b*** 8.666(0)	***c*** 25.747(1)
Space Group No.:	2	***(Å,°)***	α 84.40(0)	β 88.14(0)	γ 80.47(0)

R-Factor (%): 6.65 ***Temperature(K):*** 173 ***Density(g/cm³):*** 1.489

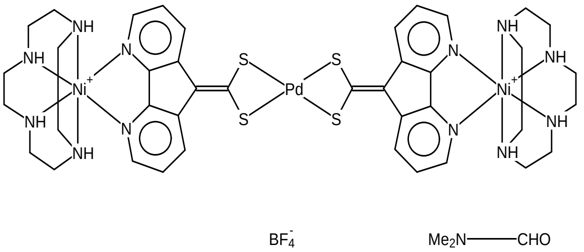

LISKON

Reference: Wai-Fun Yeung, Wing-Tak Wong, Jing-Lin Zuo, Tai-Chu Lau (2000) *J.Chem.Soc.,Dalton Trans.*,629

Formula: $C_{10} H_{20} Au_1 Cu_1 N_6{}^{1+},C_2 Au_1 N_2{}^{1-}$

Compound Name: (μ_2-Cyano)-(1,4,7,10-tetra-azacyclododecane)-cyano-copper(ii)-gold(i) dicyano-gold(i)

Space Group:	Pbcn	***Cell:***	***a*** 7.267(1)	***b*** 31.143(2)	***c*** 16.758(2)
Space Group No.:	60	***(Å,°)***	α 90.00	β 90.00	γ 90.00

R-Factor (%): 7.48 ***Temperature(K):*** 295 ***Density(g/cm³):*** 2.57

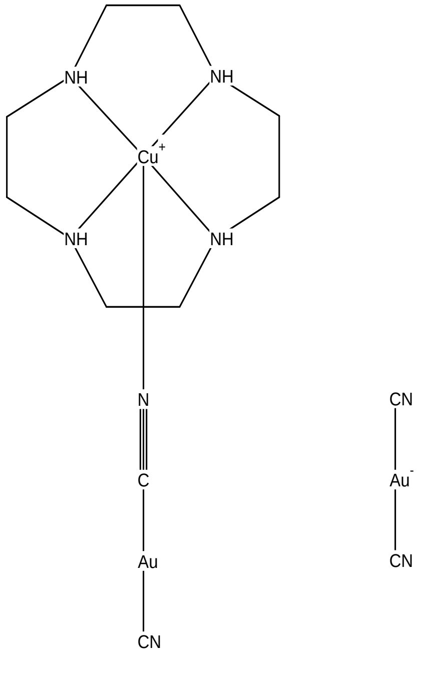

Search search1 (Mon May 04 10:12:16 2015): Hits 37-40

NAHKEN

Reference: J.Fornies, S.Ibanez, A.Martin, B.Gil, E.Lalinde, M.T.Moreno (2004) *Organometallics,* **23,** 3963

Formula: $C_{32} H_{20} Cd_1 F_{20} N_4 Pt_1,2(C_3 H_6 O_1)$

Compound Name: (1,4,7,10-Tetraazacyclodecane)-tetrakis(pentafluorophenyl)-cadmium(ii)-platinum(ii) acetone solvate

Space Group:	C2/c	***Cell:***	***a*** 23.837(2)	***b*** 9.963(0)	***c*** 17.496(1)
Space Group No.:	15	***(Å,°)***	α 90.00	β 96.82(0)	γ 90.00

R-Factor (%): 3.4 ***Temperature(K):*** 173 ***Density(g/cm³):*** 2.035

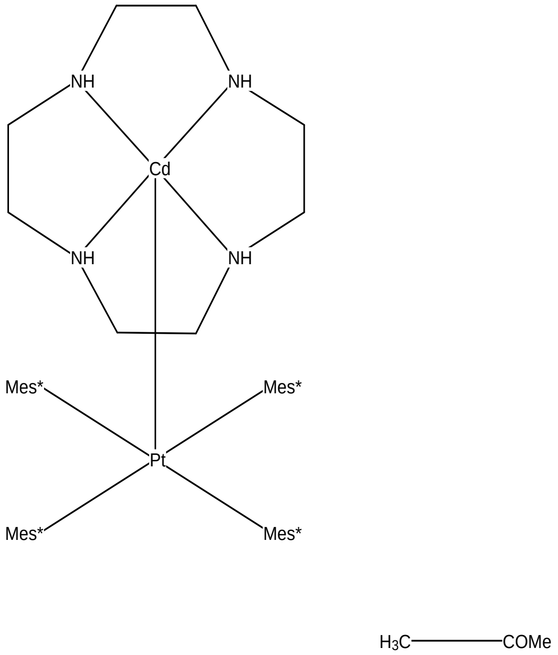

NAHKIR

Reference: J.Fornies, S.Ibanez, A.Martin, B.Gil, E.Lalinde, M.T.Moreno (2004) *Organometallics,* **23,** 3963

Formula: $C_{36} H_{30} Cd_1 F_{10} N_4 Pt_1,2(C_3 H_6 O_1)$

Compound Name: (μ_2-2-Phenylacetylen-1,1-diyl)-(2-phenylacetylenyl)-(1,4,7,10-tetraazacyclotetradecane)-bis(pentafluorophenyl)-cadmium(ii)-platinum(ii) acetone solvate

Space Group:	Pbca	***Cell:***	***a*** 16.534(0)	***b*** 19.222(0)	***c*** 27.539(0)
Space Group No.:	61	***(Å,°)***	α 90.00	β 90.00	γ 90.00

R-Factor (%): 3.71 ***Temperature(K):*** 173 ***Density(g/cm³):*** 1.719

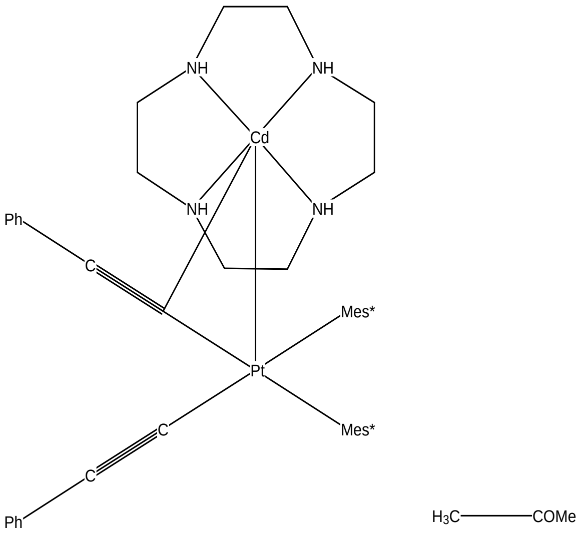

NAHKOX

Reference: J.Fornies, S.Ibanez, A.Martin, B.Gil, E.Lalinde, M.T.Moreno (2004) *Organometallics,* **23,** 3963

Formula: $C_{33} H_{28} Cd_1 F_{10} N_5 Pt_1{}^{1+},Cl_1 O_4{}^{1-},0.5(C_1 H_4 O_1)$

Compound Name: (7,8-Benzoquinolinato)-(1,4,7,10-tetraazacyclotetradecane)-bis(pentafluorophenyl)-cadmium(ii)-platinum(ii) perchlorate methanol solvate

Space Group:	P21/n	***Cell:***	***a*** 12.798(1)	***b*** 17.239(3)	***c*** 17.132(3)
Space Group No.:	14	***(Å,°)***	α 90.00	β 90.24(0)	γ 90.00

R-Factor (%): 5.1 ***Temperature(K):*** 293 ***Density(g/cm³):*** 1.946

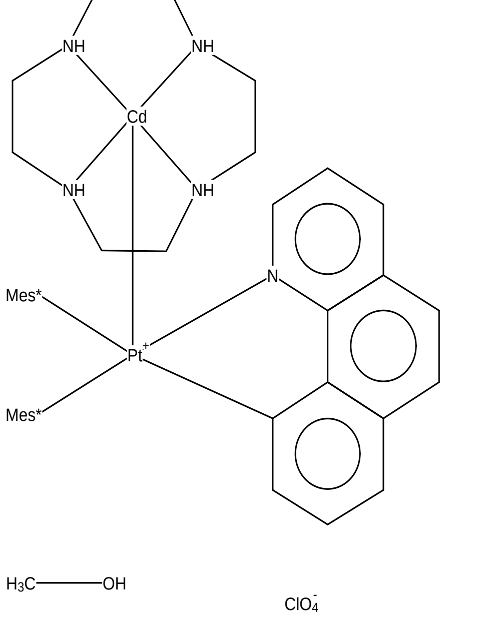

NAHKUD

Reference: J.Fornies, S.Ibanez, A.Martin, B.Gil, E.Lalinde, M.T.Moreno (2004) *Organometallics,* **23,** 3963

Formula: $C_{40} H_{40} Cd_2 Cl_4 F_{20} N_8 Pt_2,2(C_3 H_6 O_1)$

Compound Name: bis(μ_3-Chloro)-dichloro-bis(1,4,7,10-tetraazacyclotetradecane)-tetrakis(pentafluorophenyl)-di-cadmium(ii)-di-platinum(ii) acetone solvate

Space Group:	P-1	***Cell:***	***a*** 9.796(1)	***b*** 12.198(1)	***c*** 12.651(1)
Space Group No.:	2	***(Å,°)***	α 79.63(0)	β 81.82(0)	γ 74.38(0)

R-Factor (%): 4.4 ***Temperature(K):*** 100 ***Density(g/cm³):*** 2.197

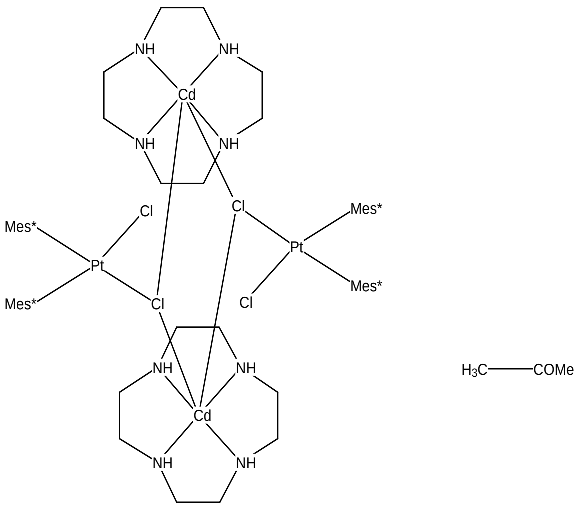

Search search1 (Mon May 04 10:12:16 2015): Hits 41-44

NAHLAK

Reference: J.Fornies, S.Ibanez, A.Martin, B.Gil, E.Lalinde, M.T.Moreno (2004) *Organometallics,* **23,** 3963

Formula: $C_{16}\ H_{40}\ Cd_2\ Cl_2\ N_8\ ^{2+}$,2($Cl_1\ O_4\ ^{1-}$)

Compound Name: bis(μ_2-Chloro)-bis(1,4,7,10-tetraazatetradecane)-di-cadmium(ii) diperclorate

Space Group:	Pnma	***Cell:***	***a*** 12.537(1)	***b*** 15.237(1)	***c*** 15.552(1)		
Space Group No.:	62	***(Å,°)***	α 90.00	β 90.00	γ 90.00		
R-Factor (%):	4.33	***Temperature(K):***	173	***Density(g/cm^3):***	1.876		

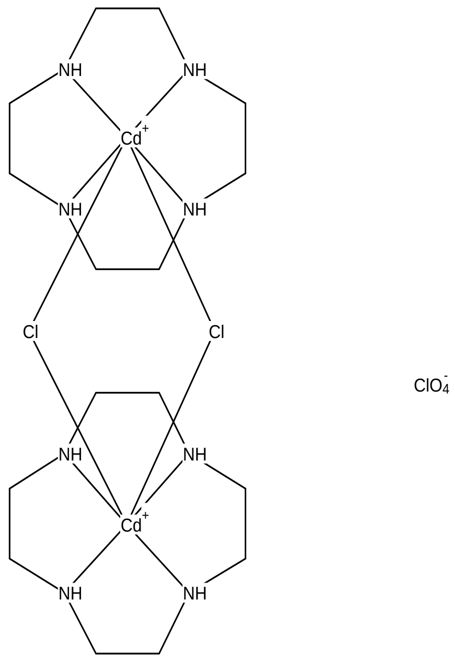

NEZDEB

Reference: K.Kobayashi, M.Shionoya, E.Kimura, K.Tsuboyama, S.Tsuboyama (1995) *Anal.Sci.,* **11,** 1029

Formula: $C_{16}\ H_{36}\ Cl_1\ N_4\ Zn_1\ ^{1+}$,$Cl_1\ O_4\ ^{1-}$

Compound Name: Chloro-((2S,5S,8S,11S)-2,5,8,11-tetraethyl-1,4,7,10-tetra-azacyclododecane)-zinc(ii) perchlorate

Space Group:	P212121	***Cell:***	***a*** 15.638(8)	***b*** 16.692(4)	***c*** 9.611(6)		
Space Group No.:	19	***(Å,°)***	α 90.00	β 90.00	γ 90.00		
R-Factor (%):	7.5	***Temperature(K):***	295	***Density(g/cm^3):***	1.283		

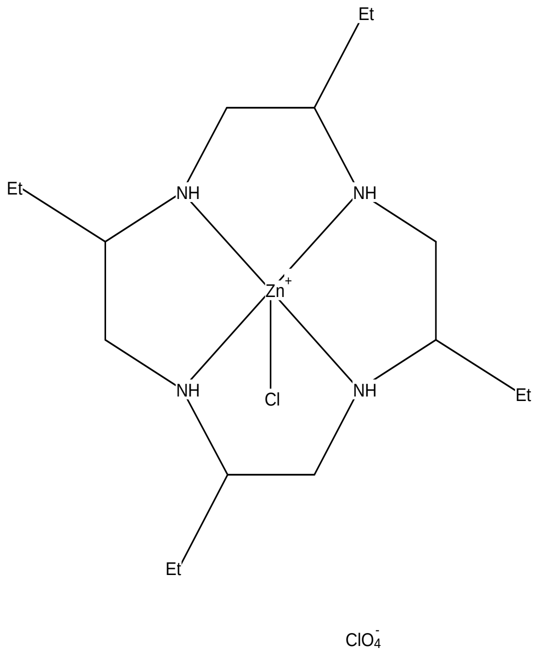

NOPCOK

Reference: A.Schrodt, A.Neubrand, R.van Eldik (1997) *Inorg.Chem.,* **36,** 4579

Formula: $C_{25}\ H_{60}\ N_{12}\ O_3\ Zn_3\ ^{4+}$,4($Cl_1\ O_4\ ^{1-}$),2($H_2\ O_1$)

Compound Name: (μ_3-Carbonato)-tris((1,4,7,10-tetra-azacyclododecane)-zinc(ii)) tetraperchlorate dihydrate

Space Group:	Pbca	***Cell:***	***a*** 21.852(5)	***b*** 21.854(5)	***c*** 21.845(5)		
Space Group No.:	61	***(Å,°)***	α 90.00	β 90.00	γ 90.00		
R-Factor (%):	8.8	***Temperature(K):***	295	***Density(g/cm^3):***	1.537		

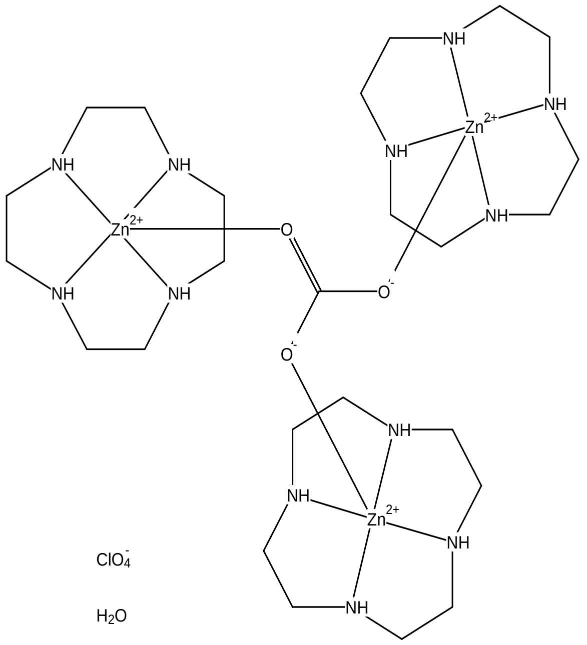

NOPCUQ

Reference: A.Schrodt, A.Neubrand, R.van Eldik (1997) *Inorg.Chem.,* **36,** 4579

Formula: $C_{10}\ H_{26}\ N_4\ O_1\ Zn_1\ ^{2+}$,2($Cl_1\ O_4\ ^{1-}$),$C_2\ H_6\ O_1$

Compound Name: (1,4,7,10-Tetra-azacyclododecane)-ethanolato-zinc(ii) diperchlorate ethanol solvate

Space Group:	P21/n	***Cell:***	***a*** 12.707(5)	***b*** 11.780(5)	***c*** 15.642(5)		
Space Group No.:	14	***(Å,°)***	α 90.00	β 105.25(0)	γ 90.00		
R-Factor (%):	4.6	***Temperature(K):***	295	***Density(g/cm^3):***	1.555		

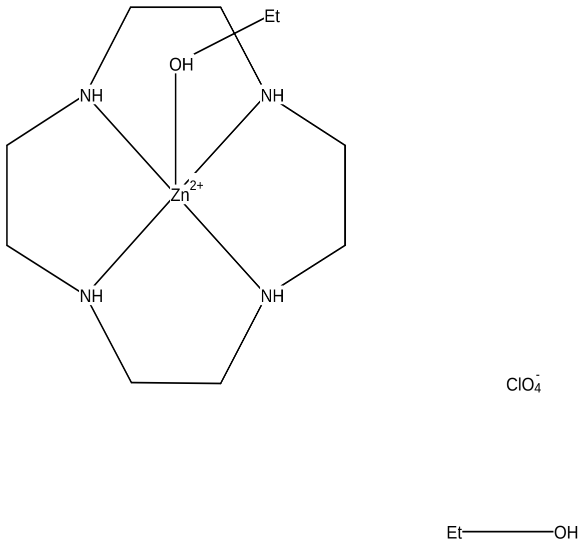

Search search1 (Mon May 04 10:12:16 2015): Hits 45-48

NUJDIF

Reference: K.Kobayashi, S.Tsuboyama, K.Tsuboyama, T.Ito (1996) *Anal.Sci.,***12,**821

Formula: C_{18} H_{40} Co_1 N_5 O_2 $^{2+}$,Cl_1 O_4 $^{1-}$,Br_1 $^{1-}$,H_2 O_1

Compound Name: cis-(SSSR)-β_1-Glycinato((2R,5R,8R,11R)-2,5,8,11-tetraethyl-1,4,7,10-tetra-azacyclododecane)-cobalt(iii) bromide perchlorate monohydrate

Space Group:	P43	***Cell:***	***a***	14.386(4)	***b***	14.386(4)	***c***	12.970(7)
Space Group No.:	78	***(Å,°)***	α	90.00	β	90.00	γ	90.00

R-Factor (%): 4.0 ***Temperature(K):*** 295 ***Density(g/cm^3):*** 1.521

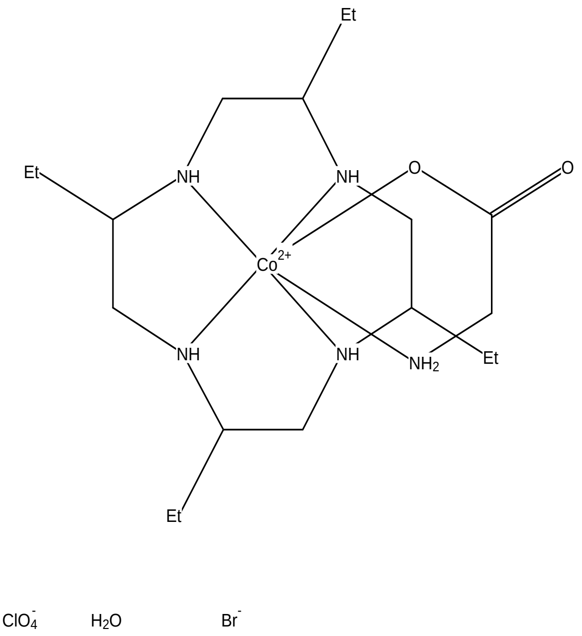

PAVYAN

Reference: Yan-Wei Ren, Jun Li, Su-Min Zhao, Feng-Xing Zhang (2005) *Struct.Chem.,***16,**439

Formula: C_8 H_{24} N_4 Ni_1 O_2 $^{2+}$,C_8 H_4 O_4 $^{2-}$

Compound Name: cis-Diaqua-(1,4,7,10-tetra-azacyclotetradecane-N,N',N'',N''')-nickel(ii) terephthalate

Space Group:	Ibca	***Cell:***	***a***	11.917(6)	***b***	17.232(8)	***c***	20.091(9)
Space Group No.:	73	***(Å,°)***	α	90.00	β	90.00	γ	90.00

R-Factor (%): 5.55 ***Temperature(K):*** 298 ***Density(g/cm^3):*** 1.388

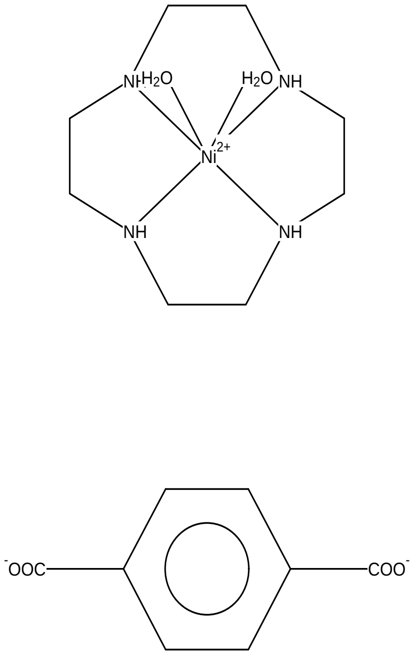

PAVYER

Reference: Yan-Wei Ren, Jun Li, Su-Min Zhao, Feng-Xing Zhang (2005) *Struct.Chem.,***16,**439

Formula: C_8 H_{22} Cu_1 N_4 O_1 $^{2+}$,C_8 H_4 O_4 $^{2-}$,3(H_2 O_1)

Compound Name: Aqua-(1,4,7,10-tetra-azacyclotetradecane-N,N',N'',N''')-copper(ii) terephthalate trihydrate

Space Group:	P-1	***Cell:***	***a***	7.815(3)	***b***	10.137(4)	***c***	14.460(5)
Space Group No.:	2	***(Å,°)***	α	83.04(0)	β	75.02(0)	γ	73.87(0)

R-Factor (%): 4.68 ***Temperature(K):*** 273 ***Density(g/cm^3):*** 1.477

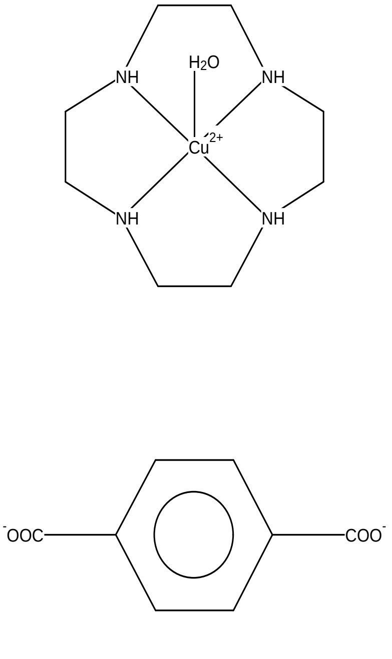

PAYPIP

Reference: Yan-Wei Ren, Jun Li, Feng-Xing Zhang, Jin-Hua Zhang, Hui Guo (2005) *Chin.J.Chem.(Engl.),***23,**418

Formula: C_{16} H_{40} Mn_2 N_8 O_2 $^{3+}$,3(Cl_1 O_4 $^{1-}$),4(H_2 O_1)

Compound Name: bis((μ_2-Oxo)-(1,4,7,11-tetra-azacyclododecane))-di-manganese(iii,iv) triperchlorate tetrahydrate

Space Group:	Cmca	***Cell:***	***a***	16.033(16)	***b***	15.746(15)	***c***	13.818(13)
Space Group No.:	64	***(Å,°)***	α	90.00	β	90.00	γ	90.00

R-Factor (%): 6.64 ***Temperature(K):*** 293 ***Density(g/cm^3):*** 1.631

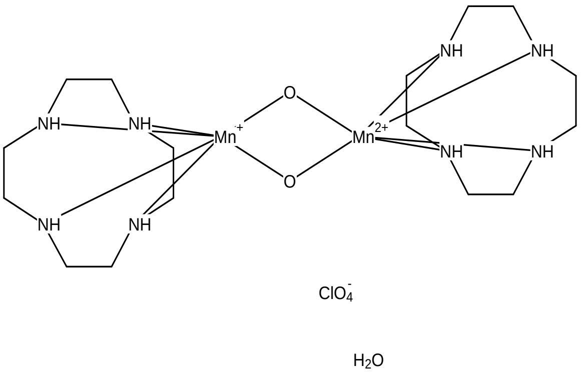

Search search1 (Mon May 04 10:12:16 2015): Hits 49-52

PERZAN

Reference: M.Shionoya, E.Kimura, M.Shiro (1993) *J.Am.Chem.Soc.*, **115,**6730

Formula: $C_{18}\,H_{32}\,N_9\,O_4\,Zn_1{}^{1+},Cl_1\,O_4{}^{1-},2(H_2\,O_1)$

Compound Name: (1,4,7,10-Tetra-azacyclododecane)-(3'-azido-3'-deoxythymidine-N3)-zinc(ii) perchlorate dihydrate

Space Group: P21 ***Cell:*** ***a*** 8.950(4) ***b*** 34.966(4) ***c*** 8.843(3)
Space Group No.: 4 ***(Å,°)*** α 90.00 β 93.13(3) γ 90.00

R-Factor (%): 7.8 ***Temperature(K):*** 295 ***Density(g/cm^3):*** 1.537

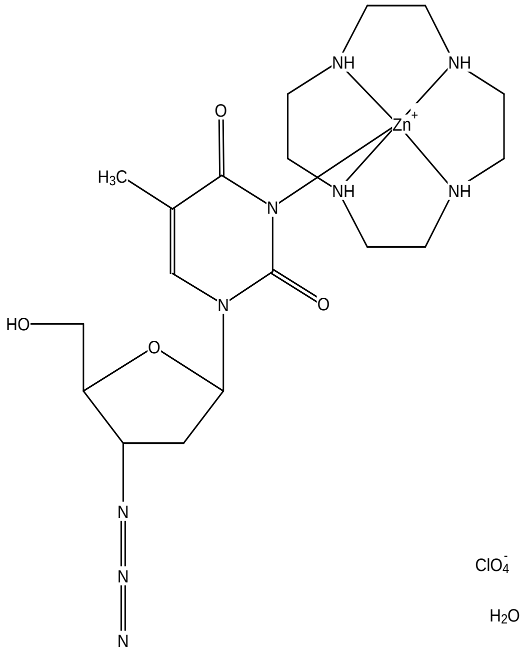

PIFDIS

Reference: Jun Du, Mo Jia, Yi-Zhi Li (2007) *Acta Crystallogr.,Sect.E(Structure Rep.Online)*,**63,**'m1629'

Formula: $C_8\,H_{20}\,I_1\,N_4\,Zn_1{}^{1+},I_3{}^{1-}$

Compound Name: Iodo-(1,4,7,10-tetra-azacyclododecane)-zinc(ii) tri-iodide

Space Group: P-1 ***Cell:*** ***a*** 8.596(1) ***b*** 9.120(1) ***c*** 12.342(2)
Space Group No.: 2 ***(Å,°)*** α 94.89(0) β 104.71(0) γ 91.30(0)

R-Factor (%): 6.34 ***Temperature(K):*** 298 ***Density(g/cm^3):*** 2.657

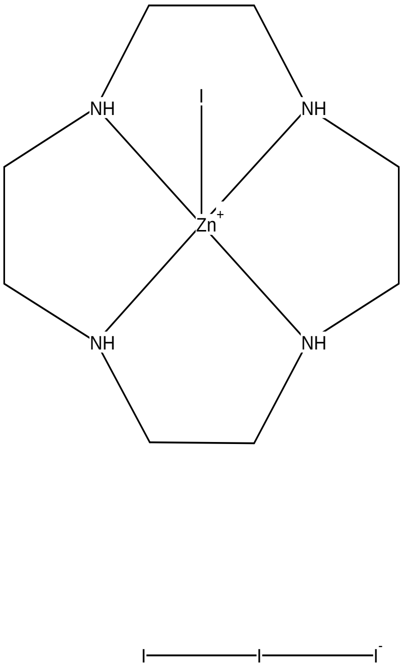

PUPPEV

Reference: K.Kobayashi, S.Tsuboyama, N.Tabata, K.Tsuboyama, T.Sakurai (1996) *Anal.Sci.*,**12,**531

Formula: $C_{21}\,H_{46}\,Co_1\,N_5\,O_2\,S_1{}^{2+},2(Cl_1\,O_4{}^{1-}),H_2\,O_1$

Compound Name: (R)-Methionato-(SSSR)-((2R,5R,8R,11R)-2,5,8,11-tetraethyl-1,4,7,10-tetra-azacyclododecane)-cobalt(iii) diperchlorate monohydrate

Space Group: P212121 ***Cell:*** ***a*** 15.677(3) ***b*** 17.036(4) ***c*** 11.878(3)
Space Group No.: 19 ***(Å,°)*** α 90.00 β 90.00 γ 90.00

R-Factor (%): 8.0 ***Temperature(K):*** 295 ***Density(g/cm^3):*** 1.484

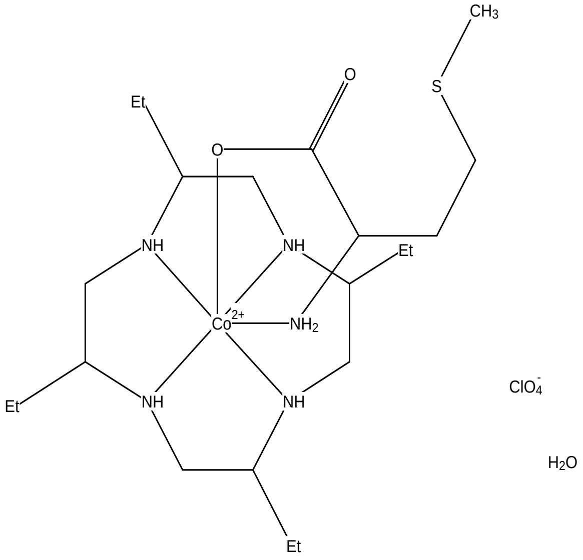

PUPPIZ

Reference: K.Kobayashi, S.Tsuboyama, N.Tabata, K.Tsuboyama, T.Sakurai (1996) *Anal.Sci.*,**12,**531

Formula: $C_{21}\,H_{46}\,Co_1\,N_5\,O_2\,S_1{}^{2+},2(Cl_1\,O_4{}^{1-}),H_2\,O_1$

Compound Name: (S)-Methionato-(SSSR)-((2R,5R,8R,11R)-2,5,8,11-tetraethyl-1,4,7,10-tetra-azacyclododecane)-cobalt(iii) diperchlorate monohydrate

Space Group: P212121 ***Cell:*** ***a*** 15.486(3) ***b*** 17.015(4) ***c*** 11.954(2)
Space Group No.: 19 ***(Å,°)*** α 90.00 β 90.00 γ 90.00

R-Factor (%): 6.0 ***Temperature(K):*** 295 ***Density(g/cm^3):*** 1.494

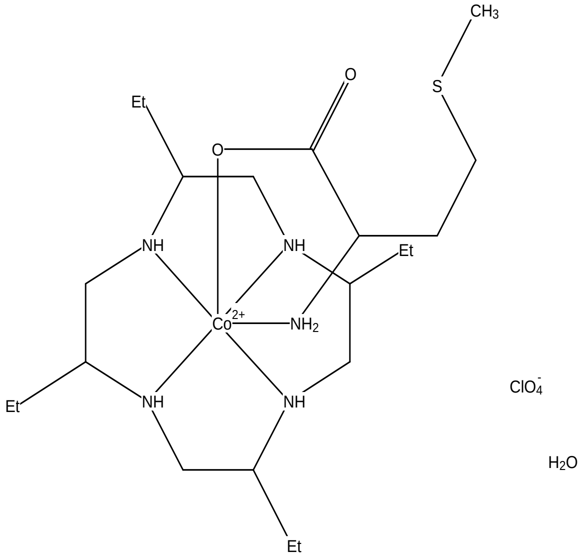

Search search1 (Mon May 04 10:12:16 2015): Hits 53-56

REMPIJ

Reference: L.A.Berben, M.C.Faia, N.R.M.Crawford, J.R.Long (2006)
Inorg.Chem.,**45,**6378

Formula: $C_{54}\ H_{84}\ N_{18}\ Ru_3\ ^{6+}$,6($Cl_1\ ^{1-}$),$C_4\ H_8\ O_1$,31($H_2\ O_1$)

Compound Name: tris(μ_2-4,4'-bipyridine)-tris(1,4,7,10-tetraazacyclododecane)-tri-ruthenium(ii) hexachloride tetrahydrofuran clathrate hydrate

Space Group: P-62m ***Cell:*** ***a*** 19.016(1) ***b*** 19.016(1) ***c*** 8.976(0)
Space Group No.: 189 ***(Å,°)*** α 90.00 β 90.00 γ 120.00

R-Factor (%): 4.37 ***Temperature(K):*** 293 ***Density(g/cm^3):*** 1.259

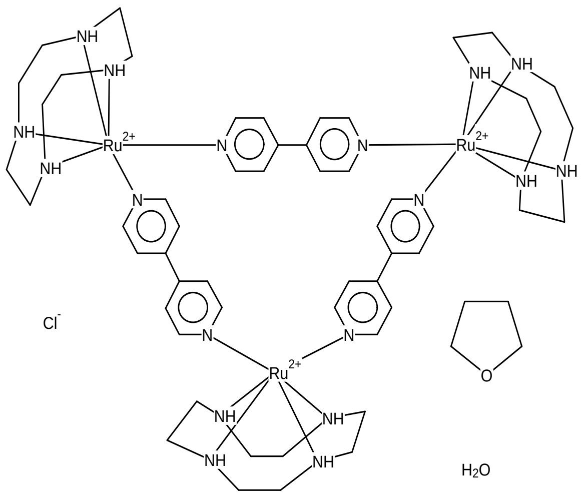

REMPOP

Reference: L.A.Berben, M.C.Faia, N.R.M.Crawford, J.R.Long (2006)
Inorg.Chem.,**45,**6378

Formula: $C_{72}\ H_{112}\ N_{24}\ Ru_4\ ^{8+}$,8($C_1\ F_3\ O_3\ S_1\ ^{1-}$),2($C_1\ H_1\ F_3\ O_3\ S_1$),5($C_1\ H_4\ O_1$)

Compound Name: tetrakis(μ_2-4,4'-bipyridine)-tetrakis(1,4,7,10-tetraazacyclododecane)-tetra-ruthenium(ii) octakis(trifluoromethanesulfonate) trifluoromethanesulfonic acid methanol solvate

Space Group: C-1 ***Cell:*** ***a*** 39.586(8) ***b*** 9.325(1) ***c*** 24.118(5)
Space Group No.: 2 ***(Å,°)*** α 90.00 β 121.96(1) γ 90.00

R-Factor (%): 10.03 ***Temperature(K):*** 193 ***Density(g/cm^3):*** 1.482

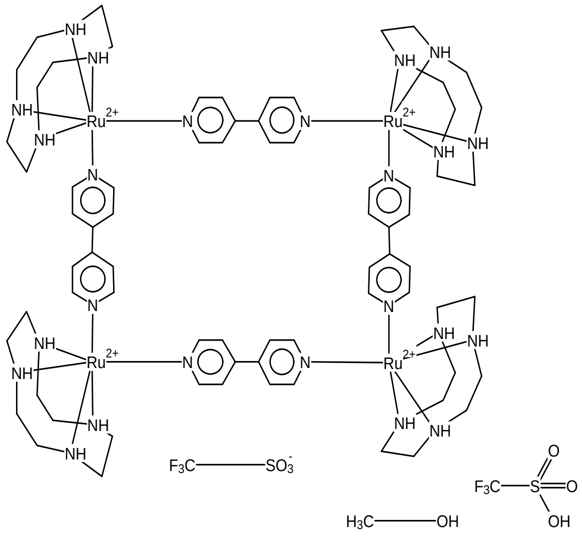

REMPUV

Reference: L.A.Berben, M.C.Faia, N.R.M.Crawford, J.R.Long (2006)
Inorg.Chem.,**45,**6378

Formula: $C_{12}\ H_{29}\ N_5\ O_1\ Ru_1\ S_1\ ^{2+}$,2($B_1\ F_4\ ^{1-}$)

Compound Name: (acetonitrile)-(dimethyl sulfoxide)-(1,4,7,10-tetraazacyclododecane)-ruthenium(ii) bis(tetrafluoroborate)

Space Group: Pc ***Cell:*** ***a*** 9.016(1) ***b*** 19.309(2) ***c*** 12.373(1)
Space Group No.: 7 ***(Å,°)*** α 90.00 β 90.24(0) γ 90.00

R-Factor (%): 6.75 ***Temperature(K):*** 293 ***Density(g/cm^3):*** 1.746

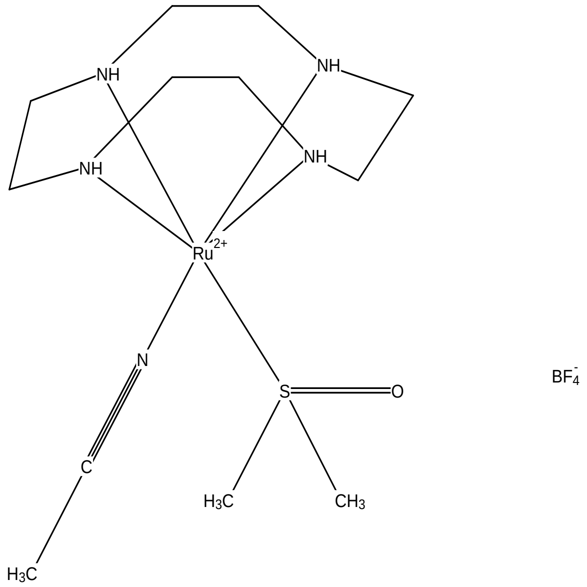

ROBCIU

Reference: E.Kimura, S.Aoki, T.Koike, M.Shiro (1997)
J.Am.Chem.Soc.,**119,**3068

Formula: $C_{30}\ H_{64}\ N_{13}\ O_6\ P_1\ Zn_3\ ^{4+}$,4($Cl_1\ O_4\ ^{1-}$),2($H_2\ O_1$)

Compound Name: (μ_3-4-Nitrophenylphosphato)-tris((1,4,7,10-tetra-azacyclododecane)-zinc(ii)) tetraperchlorate dihydrate

Space Group: P21/a ***Cell:*** ***a*** 18.481(8) ***b*** 13.901(3) ***c*** 22.556(6)
Space Group No.: 14 ***(Å,°)*** α 90.00 β 111.80(3) γ 90.00

R-Factor (%): 6.1 ***Temperature(K):*** 295 ***Density(g/cm^3):*** 1.684

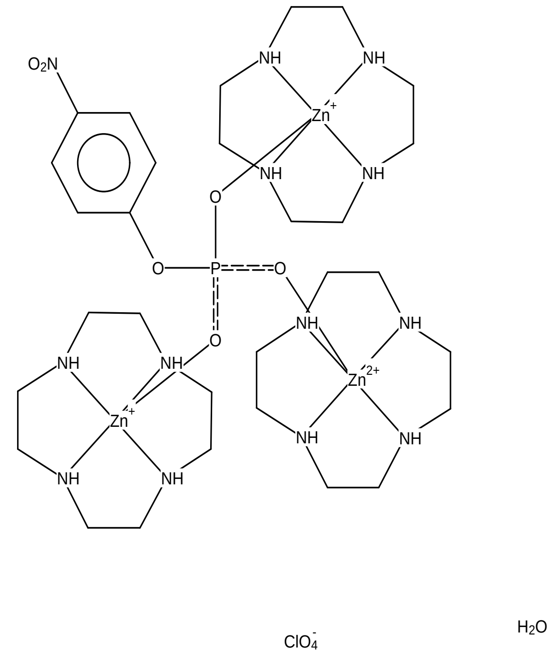

Search search1 (Mon May 04 10:12:16 2015): Hits 57-60

ROKLEI

Reference: Tian-Huey Lu, Jyh-Liong Lin, Wei-Jen Lan, Chung-Sun Chung (1997) *Acta Crystallogr.,Sect.C(Cr.Str.Comm.),* **53,**1598

Formula: $2(C_9\ H_{20}\ Cu_1\ N_5\ S_1\ ^{1+}),C_4\ H_4\ Ca_1\ N_4\ O_2\ S_4\ ^{2-},2(H_2\ O_1)$

Compound Name: bis((1,4,7,10-Tetra-azacyclododecane)-thiocyanato-copper(ii)) diaqua-tetraisothiocyanato-calcium dihydrate

Space Group:	P21/c	***Cell:***	***a*** 8.050(1)	***b*** 12.490(2)	***c*** 20.193(4)		
Space Group No.:	14	***(Å,°)***	α 90.00	β 95.97(1)	γ 90.00		
R-Factor (%):	2.6	***Temperature(K):***	295	***Density(g/cm³):***	1.533		

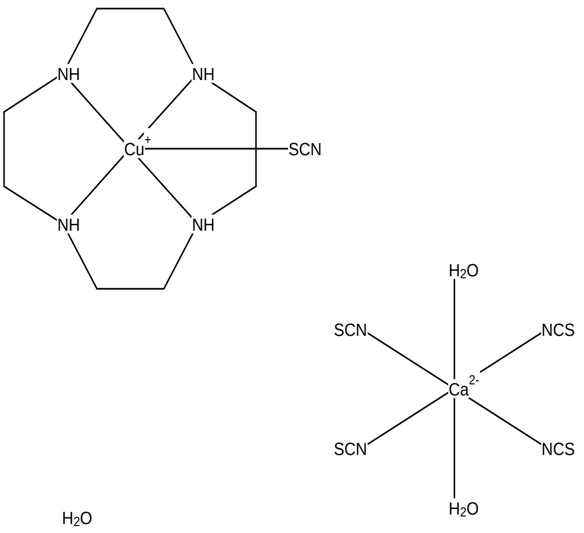

SEVMUB

Reference: D.A.Buckingham, C.R.Clark, A.J.Rogers, J.Simpson (1998) *Aust.J.Chem.*,**51,**461

Formula: $C_{11}\ H_{26}\ Co_1\ N_5\ O_2\ ^{2+},2(I_1\ ^{1-}),H_2\ O_1$

Compound Name: (1,4,7,10-Tetra-azacyclododecane)-((S)-alaninato-N,O)-cobalt di-iodide monohydrate

Space Group:	P43212	***Cell:***	***a*** 8.552(0)	***b*** 8.552(0)	***c*** 51.869(1)		
Space Group No.:	96	***(Å,°)***	α 90.00	β 90.00	γ 90.00		
R-Factor (%):	3.43	***Temperature(K):***	133	***Density(g/cm³):***	2.07		

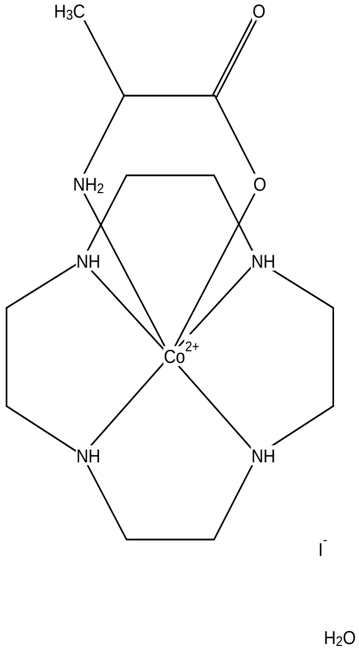

SEVNIQ

Reference: D.A.Buckingham, C.R.Clark, A.J.Rogers, J.Simpson (1998) *Aust.J.Chem.*,**51,**461

Formula: $C_{11}\ H_{26}\ Co_1\ N_5\ O_2\ ^{2+},2(Cl_1\ O_4\ ^{1-}),H_2\ O_1$

Compound Name: (1,4,7,10-Tetra-azacyclododecane)-((S)-alaninato-N,O)-cobalt diperchlorate monohydrate

Space Group:	P212121	***Cell:***	***a*** 8.499(3)	***b*** 14.538(5)	***c*** 16.592(4)		
Space Group No.:	19	***(Å,°)***	α 90.00	β 90.00	γ 90.00		
R-Factor (%):	3.88	***Temperature(K):***	193	***Density(g/cm³):***	1.737		

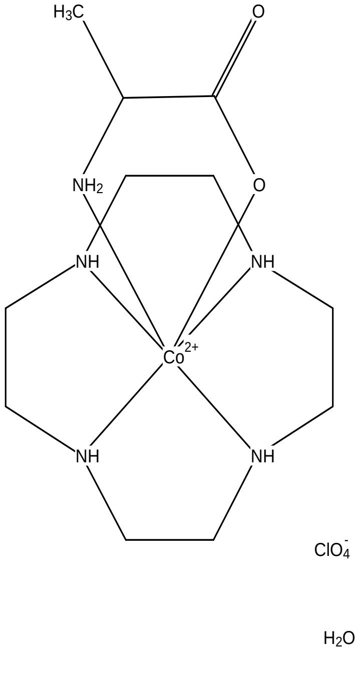

SEVQAL

Reference: D.A.Buckingham, C.R.Clark, A.J.Rogers, J.Simpson (1998) *Aust.J.Chem.*,**51,**461

Formula: $C_{11}\ H_{26}\ Co_1\ N_5\ O_2\ ^{2+},Br_4\ Zn_1\ ^{2-}$

Compound Name: (1,4,7,10-Tetra-azacyclododecane)-((S)-alaninato-N,O)-cobalt tetrabromo-zinc

Space Group:	P21/c	***Cell:***	***a*** 7.618(2)	***b*** 13.806(4)	***c*** 19.094(7)		
Space Group No.:	14	***(Å,°)***	α 90.00	β 95.76(2)	γ 90.00		
R-Factor (%):	7.26	***Temperature(K):***	193	***Density(g/cm³):***	2.341		

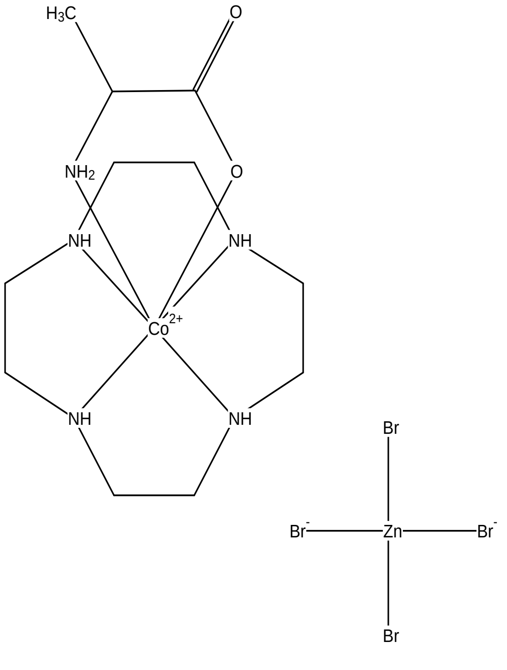

Search search1 (Mon May 04 10:12:16 2015): Hits 61-64

TAZCOC10

Reference: J.H.Loehlin, E.B.Fleischer (1976) *Acta Crystallogr.,Sect.B,***32,**3063

Formula: $C_9 H_{20} Co_1 N_4 O_3{}^{1+}, Cl_1 O_4{}^{1-}, H_2 O_1$

Compound Name: Carbonato-(1,4,7,10-tetra-azacyclododecane)-cobalt(iii) perchlorate monohydrate

Space Group: P21212 ***Cell:*** ***a*** 13.680(30) ***b*** 13.320(30) ***c*** 8.920(20)
Space Group No.: 18 ***(Å,°)*** α 90.00 β 90.00 γ 90.00

R-Factor (%): 6.7 ***Temperature(K):*** 295 ***Density(g/cm³):*** 1.67

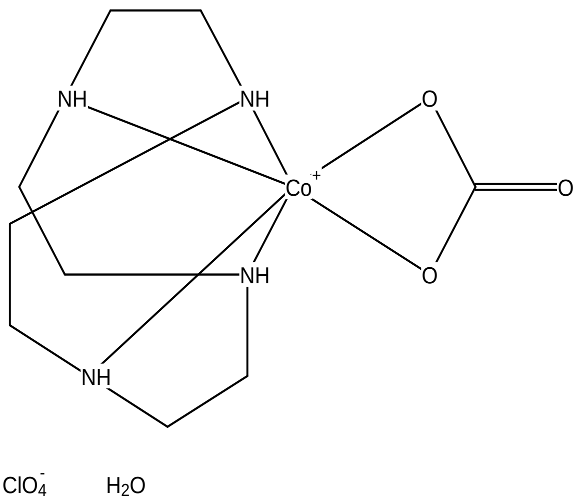

TUMTAW

Reference: S.Tsuboyama, K.Kobayashi, K.Tsuboyama, T.Sakurai (1995) *Anal.Sci.,***11,**707

Formula: $C_{20} H_{44} Co_1 N_5 O_3{}^{2+}, Cl_1 O_4{}^{1-}, Br_1{}^{1-}$

Compound Name: (R)-Threonato-(S,S,S,R)-((2R,5R,8R,11R)-2,5,8,11-tetraethyl-1,4,7,10-tetra-azacyclododecane)-cobalt(iii) perchlorate bromide

Space Group: P43 ***Cell:*** ***a*** 14.235(4) ***b*** 14.235(4) ***c*** 13.853(2)
Space Group No.: 78 ***(Å,°)*** α 90.00 β 90.00 γ 90.00

R-Factor (%): 7.4 ***Temperature(K):*** 295 ***Density(g/cm³):*** 1.516

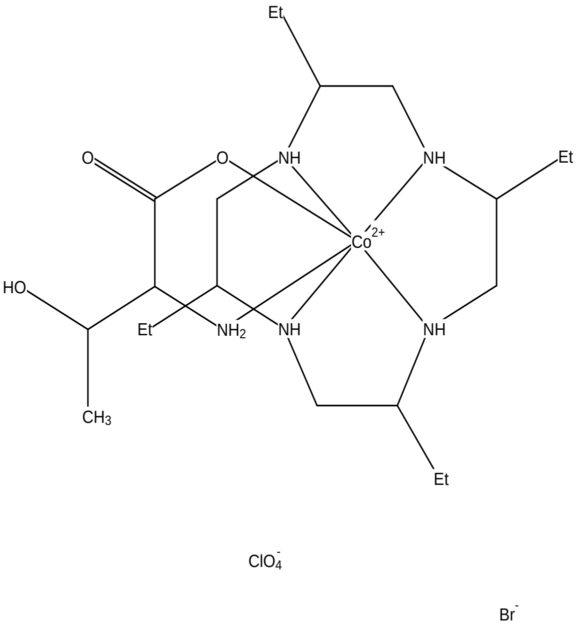

TZCDCU

Reference: R.Clay, P.Murray-Rust, J.Murray-Rust (1979) *Acta Crystallogr.,Sect.B,***35,**1894

Formula: $C_8 H_{20} Cu_1 N_5 O_3{}^{1+}, N_1 O_3{}^{1-}$

Compound Name: Nitrato-(1,4,7,10-tetra-azacyclododecane)-copper(ii) nitrate

Space Group: P21/n ***Cell:*** ***a*** 12.000(10) ***b*** 13.760(10) ***c*** 8.860(20)
Space Group No.: 14 ***(Å,°)*** α 90.00 β 90.10(3) γ 90.00

R-Factor (%): 5.2 ***Temperature(K):*** 295 ***Density(g/cm³):*** 1.634

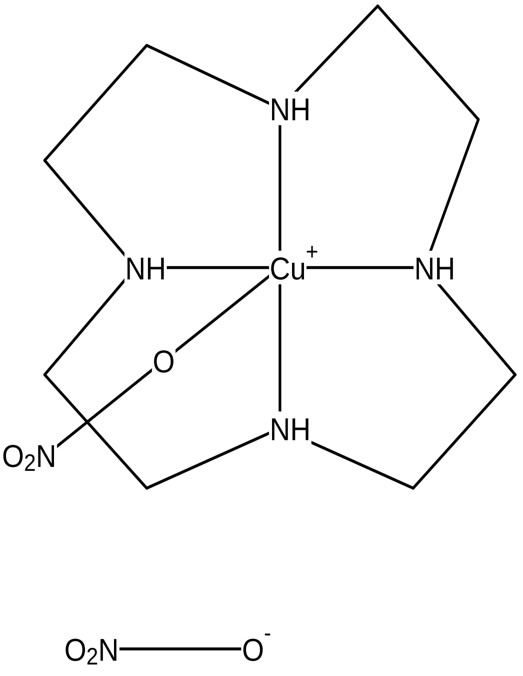

ULOBEC

Reference: Yung-Chan Lin, Tian-Huey Lu, Fen-Ling Liao, Chung-Sun Chung (2003) *Anal.Sci.,***19,**967

Formula: $C_{20} H_{28} N_6 Ni_1{}^{2+}, 2(Cl_1 O_4{}^{1-})$

Compound Name: (1,10-Phenanthroline-N,N')-(1,4,7,10-tetra-azacyclododecane-N,N',N'',N''')-nickel(ii) diperchlorate

Space Group: Pbca ***Cell:*** ***a*** 14.144(1) ***b*** 14.923(1) ***c*** 24.191(2)
Space Group No.: 61 ***(Å,°)*** α 90.00 β 90.00 γ 90.00

R-Factor (%): 5.05 ***Temperature(K):*** 295 ***Density(g/cm³):*** 1.587

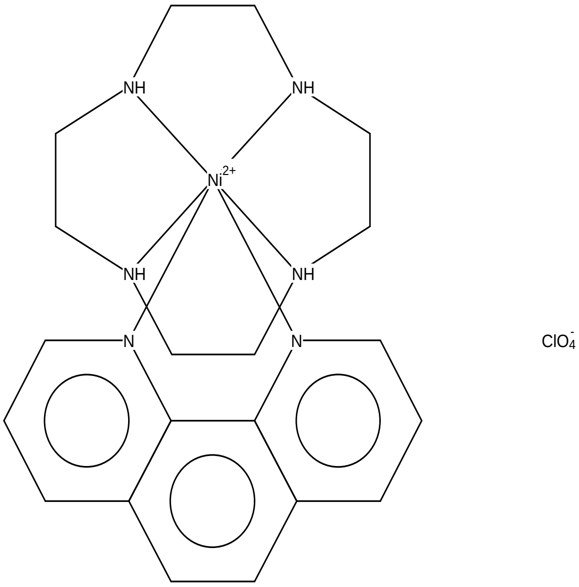

Search search1 (Mon May 04 10:12:16 2015): Hits 65-68

VANMUS

Reference: S.Tsuboyama, M.Matsudo, K.Tsuboyama, T.Sakurai (1989) *Acta Crystallogr.,Sect.C(Cr.Str.Comm.),* **45,** 872

Formula: $C_{21}\ H_{44}\ Co_1\ N_5\ O_2\ ^{2+},Br_1\ ^{1-},Cl_1\ O_4\ ^{1-},H_2\ O_1$

Compound Name: cis(SSSR)-β_1-(R)-(((R)-Prolinato-O,N)-(2R,5R,8R,11R-2,5,8,11-tetraethyl-1,4,7,10-tetra-azacyclododecane-N,N',N'',N'''))-cobalt(iii) bromide perchlorate monohydrate

Space Group:	P43	***Cell:***	***a*** 14.427(6)	***b*** 14.427(6)	***c*** 13.678(2)
Space Group No.:	78	***(Å,°)***	α 90.00	β 90.00	γ 90.00

R-Factor (%): 4.8 ***Temperature(K):*** 295 ***Density(g/cm³):*** 1.528

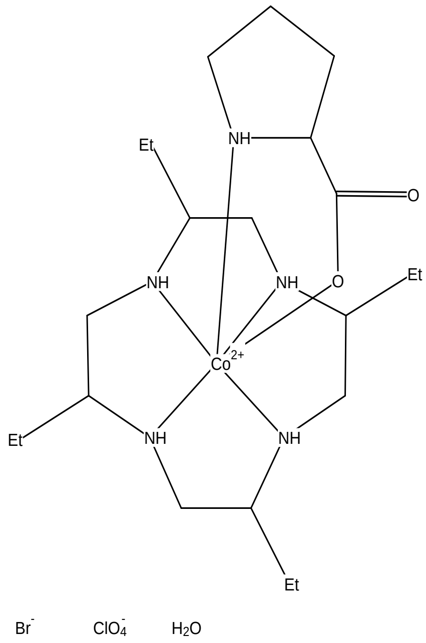

VANNAZ

Reference: S.Tsuboyama, M.Matsudo, K.Tsuboyama, T.Sakurai (1989) *Acta Crystallogr.,Sect.C(Cr.Str.Comm.),* **45,** 872

Formula: $C_{21}\ H_{44}\ Co_1\ N_5\ O_2\ ^{2+},2(Cl_1\ O_4\ ^{1-}),2(H_2\ O_1)$

Compound Name: cis(SSSR)-β_1-(S)-(((S)-Prolinato-O,N)-(2R,5R,8R,11R-2,5,8,11-tetraethyl-1,4,7,10-tetra-azacyclododecane-N,N',N'',N'''))-cobalt(iii) diperchlorate dihydrate

Space Group:	P212121	***Cell:***	***a*** 12.575(3)	***b*** 25.192(5)	***c*** 9.757(7)
Space Group No.:	19	***(Å,°)***	α 90.00	β 90.00	γ 90.00

R-Factor (%): 5.6 ***Temperature(K):*** 295 ***Density(g/cm³):*** 1.488

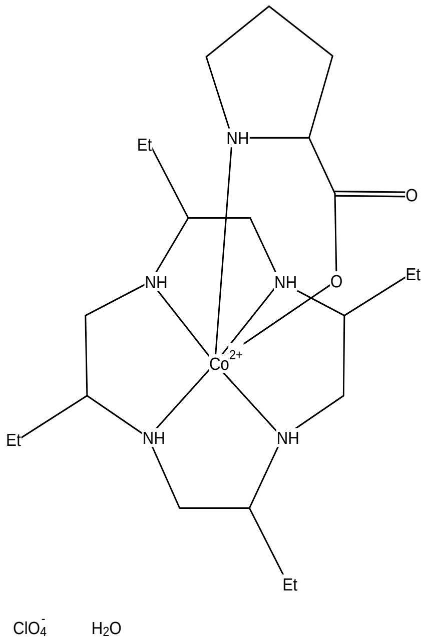

VASWIV

Reference: S.Tsuboyama, S.Miki, K.Kobayashi, K.Tsuboyama (1989) *Acta Crystallogr.,Sect.C(Cr.Str.Comm.),* **45,** 1621

Formula: $C_{17}\ H_{36}\ Co_1\ N_4\ O_3\ ^{1+},Cl_1\ O_4\ ^{1-},3(H_2\ O_1)$

Compound Name: cis(SSSR)-β_1-Carbonato-((2R,5R,8R,11R)-2,5,8,11-tetraethyl-1,4,7,10-tetra-azacyclododecane-N,N',N'',N''')-cobalt(iii) perchlorate trihydrate

Space Group:	P21	***Cell:***	***a*** 18.936(3)	***b*** 13.768(5)	***c*** 9.419(2)
Space Group No.:	4	***(Å,°)***	α 90.00	β 100.70(2)	γ 90.00

R-Factor (%): 5.7 ***Temperature(K):*** 295 ***Density(g/cm³):*** 1.533

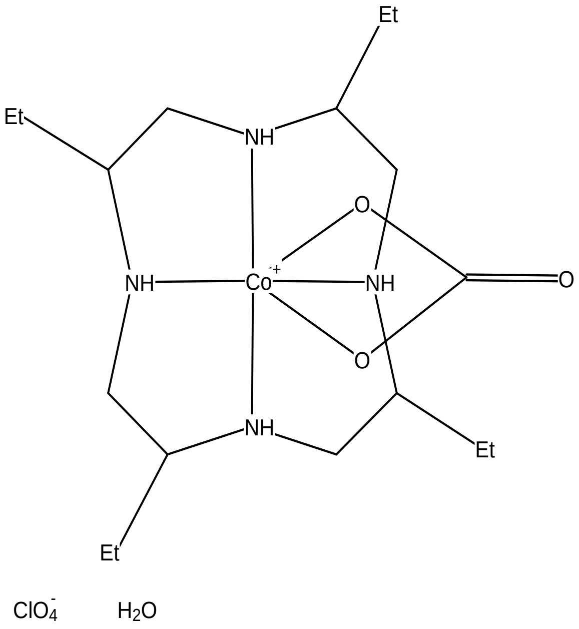

VINQIS

Reference: K.Kobayashi, H.Takahashi, M.Nishio, Y.Umezawa, K.Tsuboyama, S.Tsuboyama (2000) *Anal.Sci.,* **16,** 1103

Formula: $C_{26}\ H_{48}\ Co_1\ N_5\ O_3\ ^{2+},Br_1\ ^{1-},Cl_1\ O_4\ ^{1-},2(H_2\ O_1)$

Compound Name: ((S)-α-Methyltyrosinato-N,O)-(SSSR)-((2R,5R,8R,11R)-2,5,8,11-tetraethyl-1,4,7,10-tetra-azacyclododecane-N,N',N'',N''')-cobalt(iii) bromide perchlorate dihydrate

Space Group:	P212121	***Cell:***	***a*** 23.094(6)	***b*** 15.722(2)	***c*** 9.626(4)
Space Group No.:	19	***(Å,°)***	α 90.00	β 90.00	γ 90.00

R-Factor (%): 8.7 ***Temperature(K):*** 295 ***Density(g/cm³):*** 1.431

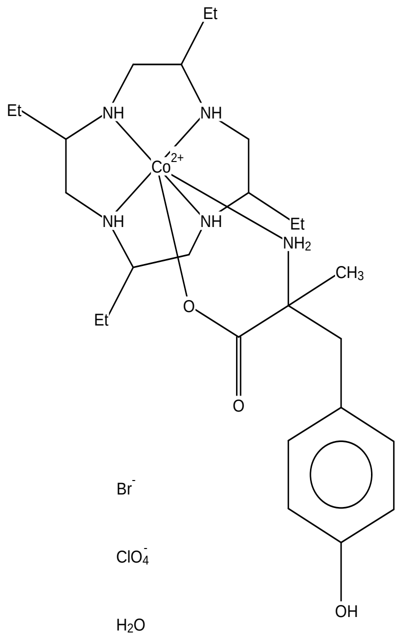

Search search1 (Mon May 04 10:12:16 2015): Hits 69-72

WABFIO

Reference:	P.A.Goodson, D.J.Hodgson, J.Glerup, K.Michelsen, H.Weihe (1992) *Inorg.Chim.Acta,* **197,**141
Formula:	$C_{16}H_{40}Mn_2N_8O_2{}^{3+}$,$H_8Li_1O_4{}^{1+}$,4($Cl_1{}^{1-}$),$H_2O_1$
Compound Name:	Tetra-aqua-lithium bis((μ_2-oxo)-(1,4,7,10-tetra-azacyclododecane-N,N',N'',N'''))-manganese(iv)-manganese(iii) tetrachloride monohydrate

Space Group:	C2/c	***Cell:***	***a***	20.284(4)	***b*** 10.576(2)	***c***	15.345(2)
Space Group No.:	15	***(Å,°)***	α	90.00	β 105.70(1)	γ	90.00

R-Factor (%): 3.44 ***Temperature(K):*** 295 ***Density(g/cm³):*** 1.52

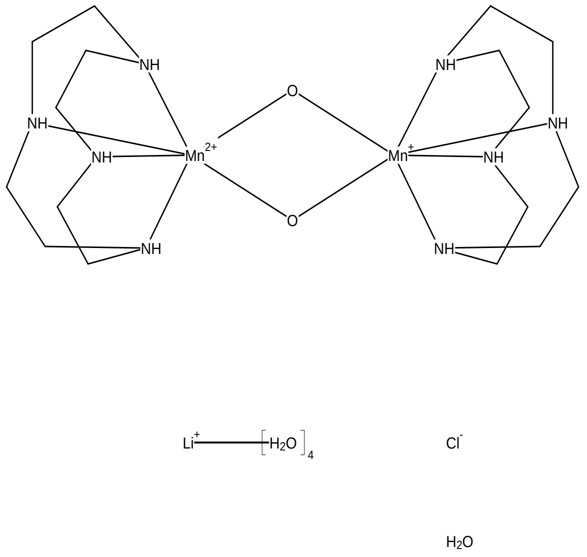

WEMFUQ

Reference:	Yuan-Jang Chen, Puhui Xie, M.J.Heeg, J.F.Endicott (2006) *Inorg.Chem.,* **45,**6282
Formula:	$C_{18}H_{28}N_6Ru_1{}^{2+}$,2($F_6P_1{}^{1-}$)
Compound Name:	(2,2'-Bipyridine-N,N')-(1,4,7,10-tetra-azacyclododecane-N,N',N'',N''')-ruthenium(ii) bis(hexafluorophosphate)

Space Group:	P21/c	***Cell:***	***a***	11.386(0)	***b*** 15.359(0)	***c***	14.345(0)
Space Group No.:	14	***(Å,°)***	α	90.00	β 90.57(0)	γ	90.00

R-Factor (%): 3.5 ***Temperature(K):*** 100 ***Density(g/cm³):*** 1.905

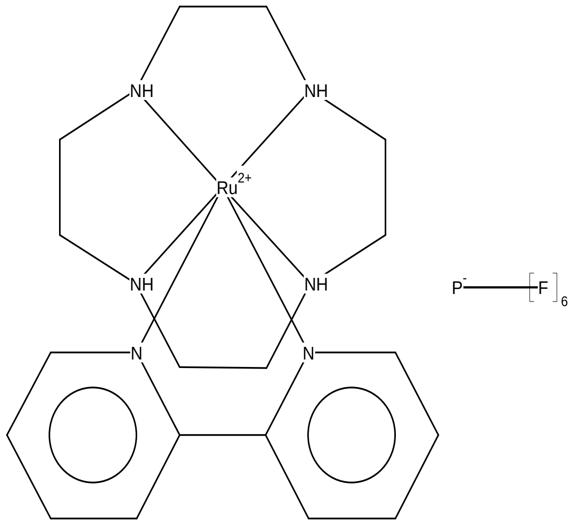

WUJLAO

Reference:	I.P.Y.Shek, Wing-Tak Wong, Song Gao, Tai-Chu Lau (2002) *New J.Chem.(Nouv.J.Chim.),* **26,**1099
Formula:	($C_{22}H_{40}Fe_1N_{14}Ni_2$)n,8n($H_2O_1$)
Compound Name:	catena-(bis(μ_3-Cyano)-tetracyano-bis(1,4,7,10-tetra-azacyclododecane)-iron(ii)-di-nickel(ii) octahydrate)

Space Group:	P2/m	***Cell:***	***a***	12.048(2)	***b*** 17.729(2)	***c***	9.155(1)
Space Group No.:	10	***(Å,°)***	α	90.00	β 112.08(1)	γ	90.00

R-Factor (%): 3.32 ***Temperature(K):*** 296 ***Density(g/cm³):*** 1.499

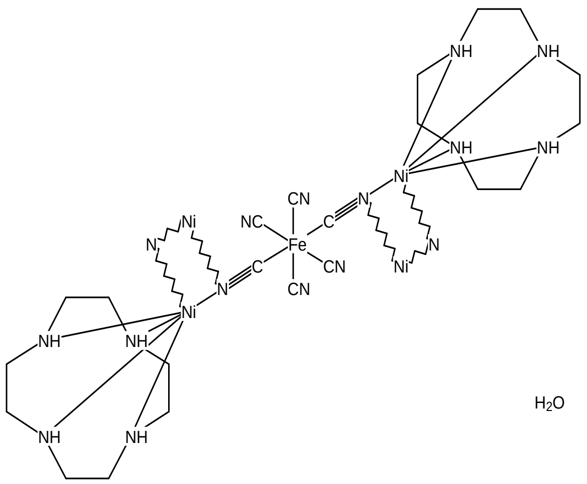

XADMAR

Reference:	Jun Li, Yan-Wei Ren, Jin-Hua Zhang, Pin Yang (2004) *J.Chem.Cryst.,* **34,**409
Formula:	$C_{10}H_{23}N_4Ni_1O_2{}^{1+}$,$B_1F_4{}^{1-}$
Compound Name:	(Acetato-O,O')-(1,4,7,10-tetra-azacyclododecane)-nickel(ii) tetrafluoroborate

Space Group:	Pnnm	***Cell:***	***a***	12.045(5)	***b*** 14.906(6)	***c***	8.913(4)
Space Group No.:	58	***(Å,°)***	α	90.00	β 90.00	γ	90.00

R-Factor (%): 5.58 ***Temperature(K):*** 298 ***Density(g/cm³):*** 1.564

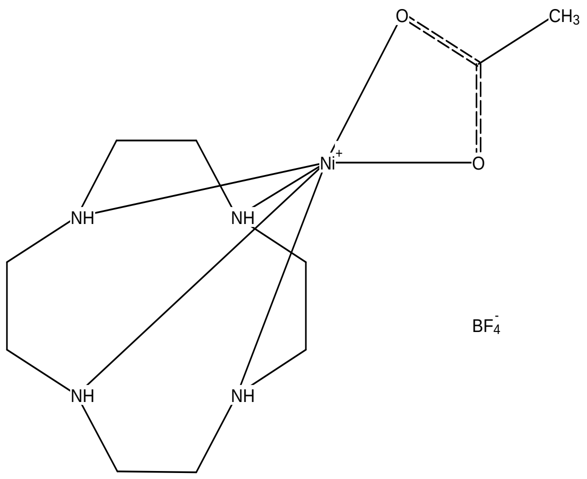

Search search1 (Mon May 04 10:12:16 2015): Hits 73-76

XAZRUL

Reference: A.J.Clarkson, D.A.Buckingham, A.J.Rogers, A.G.Blackman, C.R.Clark (2000) *Inorg.Chem.*,**39,**4769

Formula: $C_8\,H_{26}\,Co_1\,N_6\,^{3+}$,3($Cl_1\,O_4\,^{1-}$),$H_2\,O_1$

Compound Name: syn,anti-Diammine-(cyclen)-cobalt(iii) triperchlorate monohydrate

Space Group: Pnma ***Cell:*** ***a*** 17.805(4) ***b*** 12.123(3) ***c*** 9.493(2)
Space Group No.: 62 ***(Å,°)*** α 90.00 β 90.00 γ 90.00

R-Factor (%): 3.0 ***Temperature(K):*** 163 ***Density(g/cm³):*** 1.885

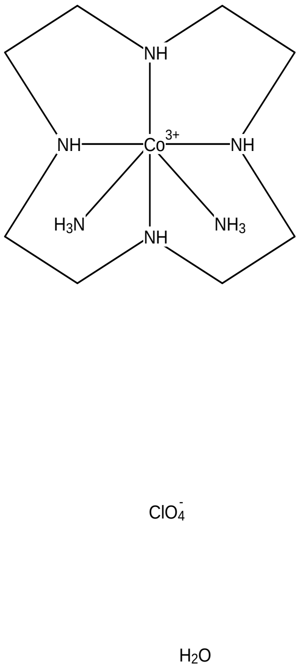

XAZSAS

Reference: A.J.Clarkson, D.A.Buckingham, A.J.Rogers, A.G.Blackman, C.R.Clark (2000) *Inorg.Chem.*,**39,**4769

Formula: $C_{10}\,H_{28}\,Co_1\,N_6\,^{3+}$,3($Cl_1\,O_4\,^{1-}$)

Compound Name: (Cyclen)-(1,2-diaminoethane)-cobalt(iii) triperchlorate

Space Group: P21/n ***Cell:*** ***a*** 8.892(2) ***b*** 15.285(3) ***c*** 15.466(3)
Space Group No.: 14 ***(Å,°)*** α 90.00 β 91.05(3) γ 90.00

R-Factor (%): 6.57 ***Temperature(K):*** 149 ***Density(g/cm³):*** 1.864

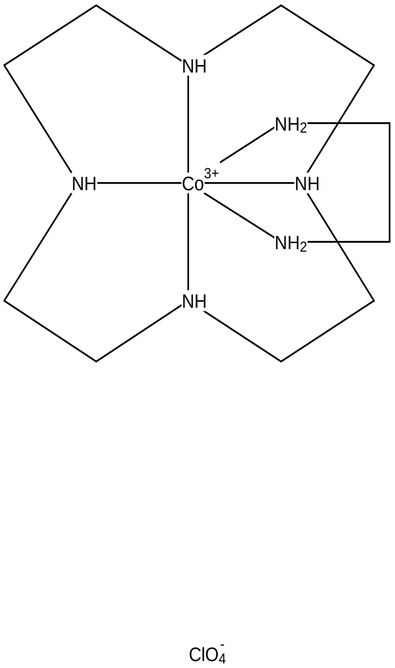

XAZSEW

Reference: A.J.Clarkson, D.A.Buckingham, A.J.Rogers, A.G.Blackman, C.R.Clark (2000) *Inorg.Chem.*,**39,**4769

Formula: $C_{11}\,H_{30}\,Co_1\,N_6\,^{3+}$,3($Br_1\,^{1-}$)

Compound Name: (Cyclen)-(propane-1,3-diamine)-cobalt(iii) tribromide

Space Group: Pca21 ***Cell:*** ***a*** 14.170(4) ***b*** 10.623(3) ***c*** 12.362(4)
Space Group No.: 29 ***(Å,°)*** α 90.00 β 90.00 γ 90.00

R-Factor (%): 2.89 ***Temperature(K):*** 168 ***Density(g/cm³):*** 1.946

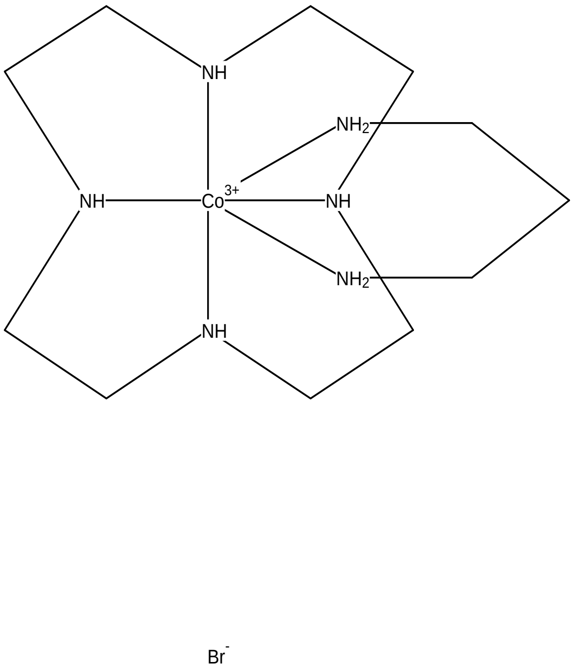

XEQTAP

Reference: Zhihui Zhang, Yanchun Lu, Jianhua Guo (2006) *J.Chem.Cryst.*,**36,**543

Formula: ($C_{18}\,H_{28}\,N_6\,Ni_1\,^{2+}$)n,2n($Cl_1\,O_4\,^{1-}$)

Compound Name: catena-((μ_2-4,4'-Bipyridine-N,N')-(1,4,7,10-tetra-azadodecane)-nickel(ii) diperchlorate)

Space Group: C2/c ***Cell:*** ***a*** 10.534(1) ***b*** 14.565(2) ***c*** 16.131(3)
Space Group No.: 15 ***(Å,°)*** α 90.00 β 102.80(0) γ 90.00

R-Factor (%): 4.41 ***Temperature(K):*** 293 ***Density(g/cm³):*** 1.613

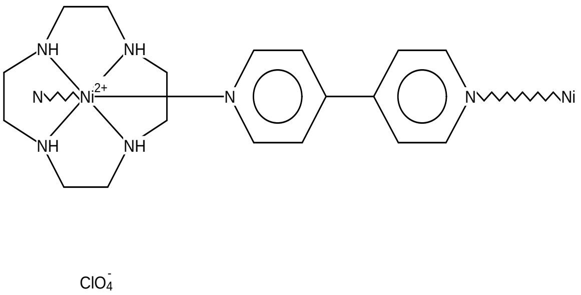

Search search1 (Mon May 04 10:12:16 2015): Hits 77-80

XESGAD

Reference: A.J.Clarkson, A.G.Blackman, C.R.Clark (2001)
J.Chem.Soc.,Dalton Trans.,758

Formula: $C_{10}\,H_{20}\,Co_1\,N_4\,O_4\,^{1+},Cl_1\,O_4\,^{1-}$

Compound Name: syn,syn-(1,4,7,10-Tetra-azacyclododecane-N,N',N'',N''')-(oxalato-O,O')-cobalt perchlorate

Space Group: Pna21 ***Cell:*** ***a*** 13.277(8) ***b*** 13.314(7) ***c*** 8.669(5)
Space Group No.: 33 ***(Å,°)*** α 90.00 β 90.00 γ 90.00

R-Factor (%): 6.09 ***Temperature(K):*** 163 ***Density(g/cm³):*** 1.815

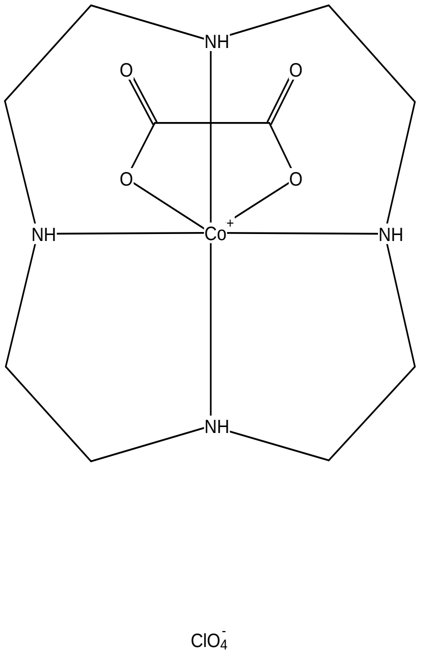

XESGEH

Reference: A.J.Clarkson, A.G.Blackman, C.R.Clark (2001)
J.Chem.Soc.,Dalton Trans.,758

Formula: $C_{10}\,H_{20}\,Co_1\,N_4\,O_4\,^{1+},Cl_1\,O_4\,^{1-}$

Compound Name: syn,anti-(1,4,7,10-Tetra-azacyclododecane-N,N',N'',N''')-(oxalato-O,O')-cobalt perchlorate

Space Group: P-1 ***Cell:*** ***a*** 7.406(4) ***b*** 8.610(5) ***c*** 14.305(10)
Space Group No.: 2 ***(Å,°)*** α 77.85(1) β 86.81(1) γ 68.77(1)

R-Factor (%): 4.8 ***Temperature(K):*** 158 ***Density(g/cm³):*** 1.673

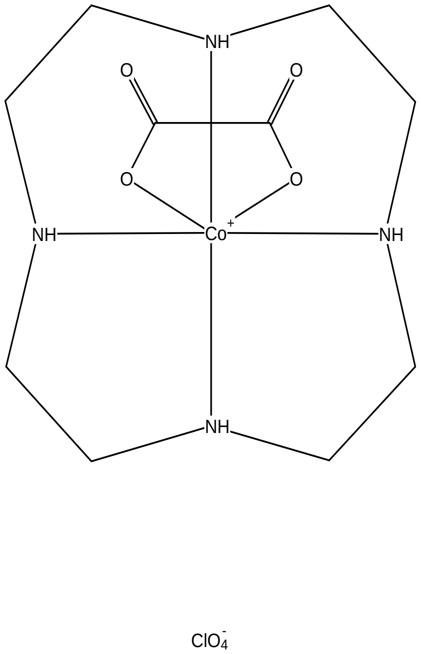

XESGIL

Reference: A.J.Clarkson, A.G.Blackman, C.R.Clark (2001)
J.Chem.Soc.,Dalton Trans.,758

Formula: $C_{11}\,H_{22}\,Co_1\,N_4\,O_4\,^{1+},Cl_1\,O_4\,^{1-},H_2\,O_1$

Compound Name: syn,anti-(1,4,7,10-Tetra-azacyclododecane-N,N',N'',N''')-(malonato-O,O')-cobalt perchlorate monohydrate

Space Group: P21/c ***Cell:*** ***a*** 7.576(0) ***b*** 14.301(1) ***c*** 15.980(1)
Space Group No.: 14 ***(Å,°)*** α 90.00 β 91.51(0) γ 90.00

R-Factor (%): 3.04 ***Temperature(K):*** 158 ***Density(g/cm³):*** 1.73

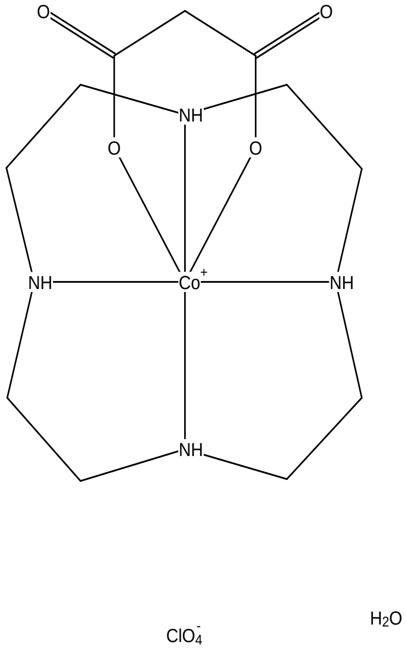

XESGOR

Reference: A.J.Clarkson, A.G.Blackman, C.R.Clark (2001)
J.Chem.Soc.,Dalton Trans.,758

Formula: $C_{11}\,H_{22}\,Co_1\,N_4\,O_4\,^{1+},Cl_1\,O_4\,^{1-},H_2\,O_1$

Compound Name: syn,syn-(1,4,7,10-Tetra-azacyclododecane-N,N',N'',N''')-(malonato-O,O')-cobalt perchlorate monohydrate

Space Group: P21/c ***Cell:*** ***a*** 15.268(5) ***b*** 7.616(2) ***c*** 15.285(5)
Space Group No.: 14 ***(Å,°)*** α 90.00 β 92.92(0) γ 90.00

R-Factor (%): 3.69 ***Temperature(K):*** 170 ***Density(g/cm³):*** 1.687

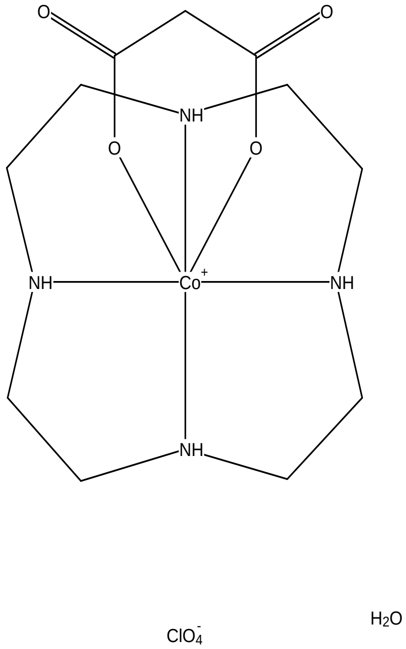

Search search1 (Mon May 04 10:12:16 2015): Hits 81-84

XESGUX

Reference: A.J.Clarkson, A.G.Blackman, C.R.Clark (2001) *J.Chem.Soc.,Dalton Trans.*,758

Formula: $C_{11}\ H_{23}\ Co_1\ N_4\ O_4\ ^{2+}$,2($Cl_1\ O_4\ ^{1-}$),2($H_2\ O_1$)

Compound Name: syn,anti-(1,4,7,10-Tetra-azacyclododecane-N,N',N'',N''')-(hydrogen malonato-O,O')-cobalt diperchlorate dihydrate

Space Group: Pbca	***Cell:***	***a*** 18.022(4)	***b*** 14.943(3)	***c*** 32.020(8)
Space Group No.: 61	***(Å,°)***	α 90.00	β 90.00	γ 90.00

R-Factor (%): 5.09 ***Temperature(K):*** 168 ***Density(g/cm³):*** 1.754

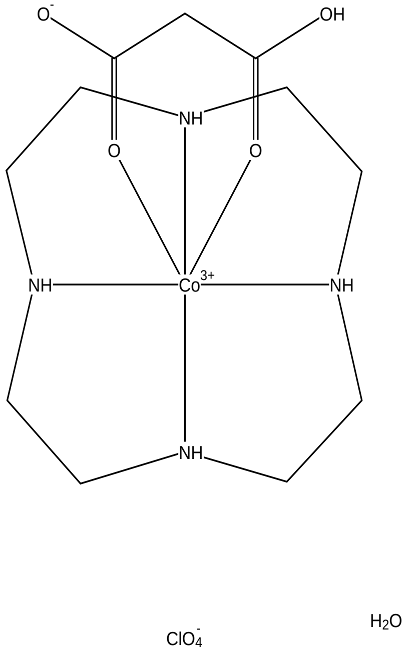

XIJKAC

Reference: C.Pariya, Ta-Yung Chi, T.K.Mishra, Chung-Sun Chung (2002) *Inorganic Chemistry Communications,* **5,**119

Formula: $C_{19}\ H_{43}\ N_{10}\ Zn_2\ ^{3+}$,3($Cl_1\ O_4\ ^{1-}$)

Compound Name: (μ_2-Imidazolato-N,N')-bis(1,4,7,10-tetraazacyclododecane)-zinc(ii) triperchlorate

Space Group: Pbca	***Cell:***	***a*** 16.546(0)	***b*** 15.459(0)	***c*** 27.174(0)
Space Group No.: 61	***(Å,°)***	α 90.00	β 90.00	γ 90.00

R-Factor (%): 7.14 ***Temperature(K):*** 293 ***Density(g/cm³):*** 1.607

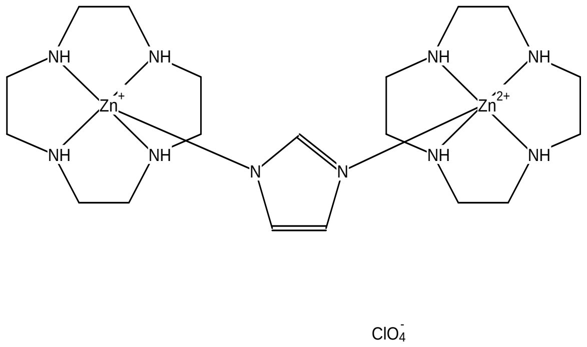

XIJKEG

Reference: C.Pariya, Ta-Yung Chi, T.K.Mishra, Chung-Sun Chung (2002) *Inorganic Chemistry Communications,* **5,**119

Formula: $C_{11}\ H_{24}\ Cl_1\ N_6\ Ni_1\ O_4\ ^{1+}$,$Cl_1\ O_4\ ^{1-}$

Compound Name: (1,4,7,10-Tetraazacyclododecane)-(3-imidazolyl)-perchlorato-nickel(ii)

Space Group: P21/n	***Cell:***	***a*** 8.899(0)	***b*** 16.185(0)	***c*** 13.658(0)
Space Group No.: 14	***(Å,°)***	α 90.00	β 94.81(0)	γ 90.00

R-Factor (%): 8.29 ***Temperature(K):*** 293 ***Density(g/cm³):*** 1.687

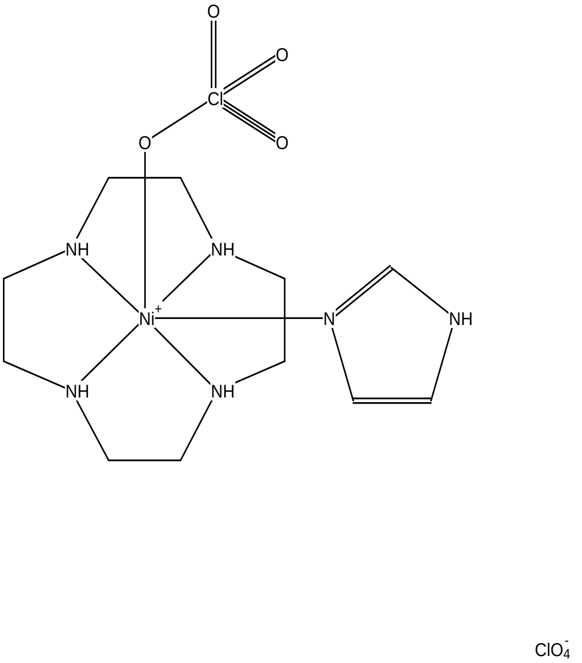

YACTIF

Reference: S.Tsuboyama, Y.Shiga, Y.Takasyo, T.Chijimatsu, K.Kobayashi, K.Tsuboyama, T.Sakurai (1992) *J.Chem.Soc.,Dalton Trans.*,1783

Formula: $C_{19}\ H_{42}\ Co_1\ N_5\ O_2\ ^{2+}$,2($Cl_1\ O_4\ ^{1-}$),2($H_2\ O_1$)

Compound Name: cis-β-(RSRS)-((S)-Alaninato-N,O)-((2R,5R,8R,11R)-2,5,8,11-tetraethyl-1, 4,7,10-tetra-azacyclododecane)-cobalt(iii) diperchlorate dihydrate

Space Group: P212121	***Cell:***	***a*** 14.962(3)	***b*** 14.729(3)	***c*** 13.675(2)
Space Group No.: 19	***(Å,°)***	α 90.00	β 90.00	γ 90.00

R-Factor (%): 8.2 ***Temperature(K):*** 295 ***Density(g/cm³):*** 1.469

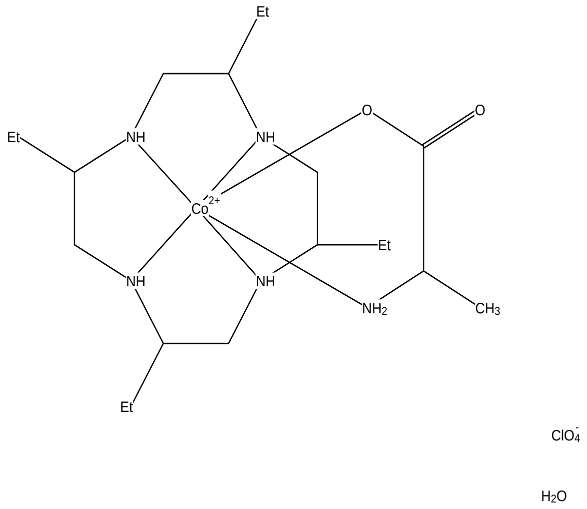

Search search1 (Mon May 04 10:12:16 2015): Hits 85-88

YACTOL

Reference: S.Tsuboyama, Y.Shiga, Y.Takasyo, T.Chijimatsu, K.Kobayashi, K.Tsuboyama, T.Sakurai (1992) _J.Chem.Soc.,Dalton Trans._,1783

Formula: C_{20} H_{44} Co_1 N_5 O_3,Cl_4 Zn_1,H_2 O_1

Compound Name: cis-β-(SSSR)-((S)-Threonato-N,O)-((2R,5R,8R,11R)-2,5,8,11-tetraethyl-1,4,7,10-tetra-azacyclododecane)-cobalt(iii) tetrachloro-zinc monohydrate

**_Space Group:_**	P212121	**_Cell:_**	**_a_**	19.152(7)	**_b_**	16.222(5)	**_c_**	9.684(3)
**_Space Group No.:_**	19	**_(Å,°)_**	α	90.00	β	90.00	γ	90.00

R-Factor (%): 10.3 **_Temperature(K):_** 295 **_Density(g/cm^3):_** 1.516

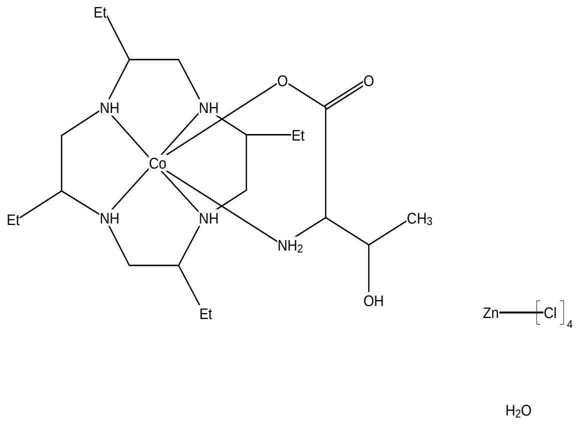

YACTUR

Reference: S.Tsuboyama, Y.Shiga, Y.Takasyo, T.Chijimatsu, K.Kobayashi, K.Tsuboyama, T.Sakurai (1992) _J.Chem.Soc.,Dalton Trans._,1783

Formula: C_{20} H_{44} Co_1 N_5 O_3 $^{2+}$,2(Cl_1 O_4 $^{1-}$),3(H_2 O_1)

Compound Name: cis-β-(RSRS)-((S)-Threonato-N,O)-((2R,5R,8R,11R)-2,5,8,11-tetraethyl-1,4,7,10-tetra-azacyclododecane)-cobalt(iii) diperchlorate trihydrate

**_Space Group:_**	P212121	**_Cell:_**	**_a_**	14.998(9)	**_b_**	14.757(5)	**_c_**	14.323(4)
**_Space Group No.:_**	19	**_(Å,°)_**	α	90.00	β	90.00	γ	90.00

R-Factor (%): 6.6 **_Temperature(K):_** 295 **_Density(g/cm^3):_** 1.497

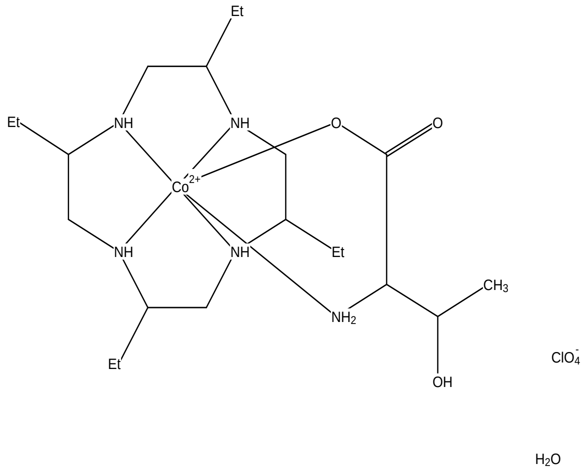

YATKAG

Reference: M.H.Salter Junior, J.H.Reibenspies, S.B.Jones, R.D.Hancock (2005) _Inorg.Chem._,**44,**2791

Formula: C_9 H_{24} N_6 S_1 Zn_1 $^{2+}$,2(N_1 O_3 $^{1-}$)

Compound Name: (1,4,7,10-Tetra-azacyclododecane)-(thiourea-S)-zinc(ii) dinitrate

**_Space Group:_**	P212121	**_Cell:_**	**_a_**	11.417(1)	**_b_**	12.200(1)	**_c_**	12.530(1)
**_Space Group No.:_**	19	**_(Å,°)_**	α	90.00	β	90.00	γ	90.00

R-Factor (%): 5.04 **_Temperature(K):_** 110 **_Density(g/cm^3):_** 1.666

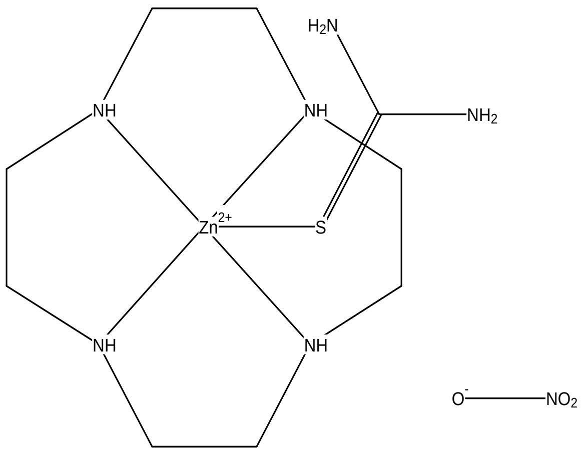

YAVQUH

Reference: Soo-Young Kim, In-Sun Jung, Eunsung Lee, Jaheon Kim, S.Sakamoto, K.Yamaguchi, Kimoon Kim (2001) _Angew.Chem.,Int.Ed.Engl._,**40,**2119

Formula: C_{48} H_{48} N_{32} O_{16},C_8 H_{22} Cu_1 N_4 O_1 $^{2+}$,2(N_1 O_3 $^{1-}$),16(H_2 O_1)

Compound Name: Aqua-cucurbit(8)uril (1,4,7,10-tetra-azacyclododecane-copper(ii)) clathrate dinitrate hexadecahydrate

**_Space Group:_**	R-3	**_Cell:_**	**_a_**	29.858(0)	**_b_**	29.858(0)	**_c_**	24.876(0)
**_Space Group No.:_**	148	**_(Å,°)_**	α	90.00	β	90.00	γ	120.00

R-Factor (%): 10.44 **_Temperature(K):_** 188 **_Density(g/cm^3):_** 1.553

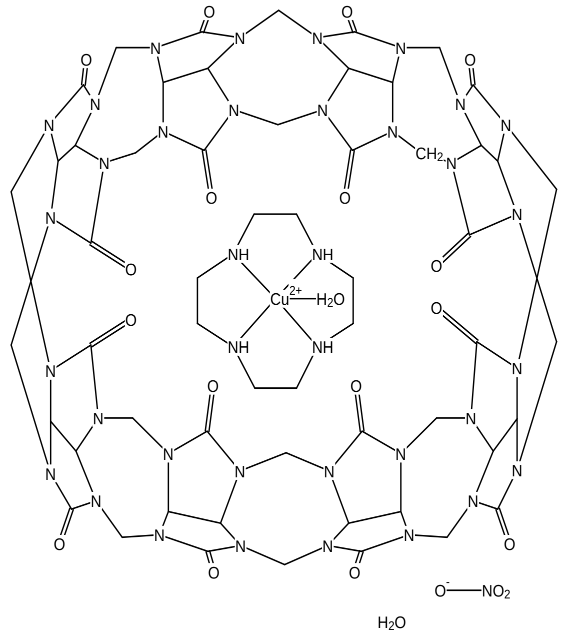

Search search1 (Mon May 04 10:12:16 2015): Hits 89-92

ZOCNAG

Reference: C.R.Randall, Lijin Shu, Yu-Min Chiou, K.S.Hagen, M.Ito, N.Kitajima, R.J.Lachicotte, Yan Zang, L.Que Junior (1995) *Inorg.Chem.*,**34,**1036

Formula: $C_{10}\,H_{23}\,Fe_1\,N_4\,O_2\,^{1+},C_1\,F_3\,O_3\,S_1\,^{1-}$

Compound Name: Acetato-(1,4,7,10-tetra-azacyclododecane)-iron trifluoromethanesulfonate

Space Group:	C2/m	***Cell:***	***a*** 18.058(1)	***b*** 9.001(1)	***c*** 11.232(1)		
Space Group No.:	12	***(Å,°)***	α 90.00	β 97.64(2)	γ 90.00		
R-Factor (%):	4.11	***Temperature(K):***	295	***Density(g/cm³):***	1.601		

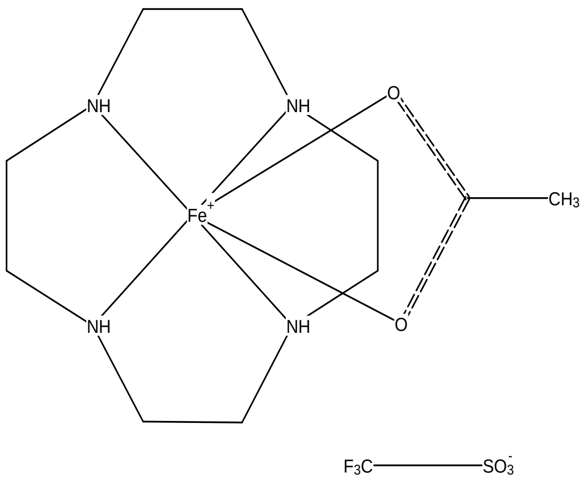

DUQXET

Reference: D.Rohde, K.Merzweiler (2010) *Acta Crystallogr.,Sect.E(Structure Rep.Online)*,**66,**'m894'

Formula: $C_8\,H_{20}\,Cu_1\,Mo_1\,N_4\,O_4,H_2\,O_1$

Compound Name: (μ_2-Oxo)-(1,4,7,10-tetra-azacyclododecane)-trioxido-copper(ii)-molybdenum monohydrate

Space Group:	P-1	***Cell:***	***a*** 8.699(0)	***b*** 8.978(0)	***c*** 9.005(0)		
Space Group No.:	2	***(Å,°)***	α 90.36(0)	β 91.95(0)	γ 100.74(0)		
R-Factor (%):	2.39	***Temperature(K):***	200	***Density(g/cm³):***	1.99		

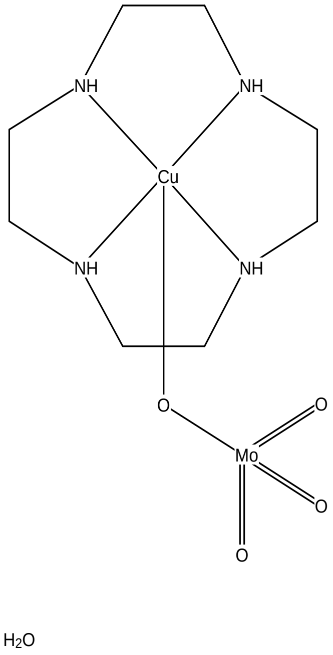

NURXII

Reference: Jun-Fang Guo, Xiu-Teng Wang, Bing-Wu Wang, Guan-Cheng Xu, Song Gao, Lap Szeto, Wing-Tak Wong, Wai-Yeung Wong, Tai-Chu Lau (2010) *Chemistry-A European Journal*,**16,**3524

Formula: $(C_{20}\,H_{34}\,N_6\,Ni_1\,O_4\,Ru_1\,^{1+})n,n(C_1\,H_4\,O_1),n(Cl_1\,O_4\,^{1-})$

Compound Name: catena-(bis(μ_2-cyanido)-bis(acetylacetonato)-(1,4,7,10-tetraazacyclododecane)-nickel(ii)-ruthenium(iii) perchlorate methanol solvate)

Space Group:	P21/c	***Cell:***	***a*** 12.316(0)	***b*** 14.925(0)	***c*** 16.736(1)		
Space Group No.:	14	***(Å,°)***	α 90.00	β 100.07(0)	γ 90.00		
R-Factor (%):	4.25	***Temperature(K):***	296	***Density(g/cm³):***	1.565		

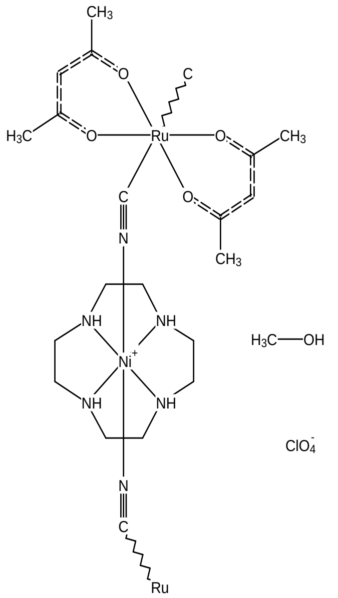

FOQBIX

Reference: Gao-mai Yang, Jun Li, Yan-wei Ren, Hui Guo, Min-yue Duan, Feng-xing Zhang, Xiongfu Zhang (2009) *Transition Met.Chem.*,**34,**191

Formula: $C_{20}\,H_{44}\,N_8\,Ni_2\,O_4\,^{2+},2(Cl_1\,^{1-}),2(H_2\,O_1)$

Compound Name: (μ_2-Succinato-O,O',O'',O''')-bis(1,4,7,10-tetra-azacyclododecane-N,N',N'',N''')-di-nickel(ii) dichloride dihydrate

Space Group:	P21/n	***Cell:***	***a*** 7.843(2)	***b*** 13.159(4)	***c*** 15.011(4)		
Space Group No.:	14	***(Å,°)***	α 90.00	β 99.20(0)	γ 90.00		
R-Factor (%):	2.58	***Temperature(K):***	273	***Density(g/cm³):***	1.488		

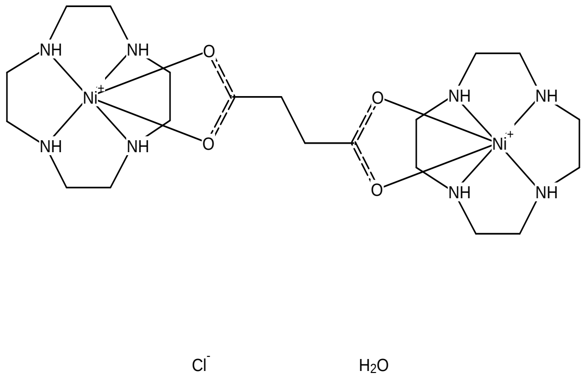

Search search1 (Mon May 04 10:12:16 2015): Hits 93-96

KUBTEH

Reference: Z.Vargova, R.Gyepes, L.Arabuli, K.Gyoryova, P.Hermann, I.Lukes (2009) *Inorg.Chim.Acta,* **362,** 3860

Formula: $C_{10}\,H_{25}\,N_5\,O_2\,Zn_1{}^{2+},2(Cl_1\,O_4{}^{1-}),H_2\,O_1$

Compound Name: (cdelta/Λ)-(trans-I-(++++)-1,4,7,10-Tetraazacyclododecane-N,N',N'',N''')-(glycine-O)-zinc(ii) diperchlorate monohydrate

Space Group:	P21/c	***Cell:***	***a*** 28.347(0)	***b*** 8.842(0)	***c*** 17.371(0)
Space Group No.:	14	***(Å,°)***	α 90.00	β 107.89(0)	γ 90.00

R-Factor (%): 4.18 ***Temperature(K):*** 150 ***Density(g/cm³):*** 1.698

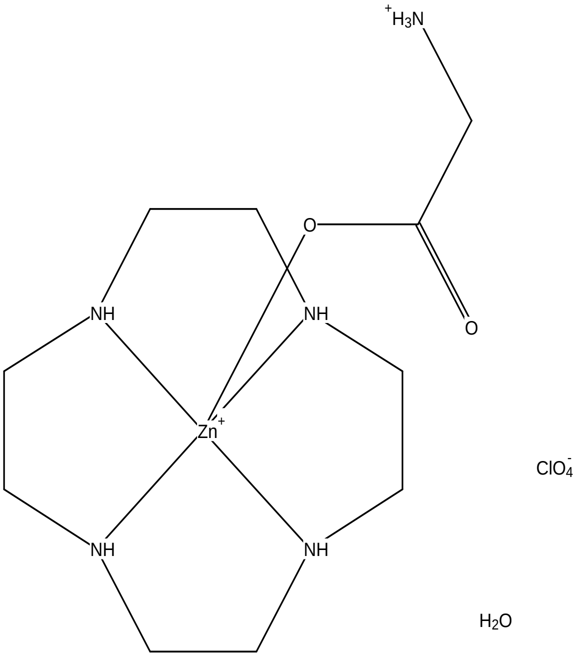

KUBTIL

Reference: Z.Vargova, R.Gyepes, L.Arabuli, K.Gyoryova, P.Hermann, I.Lukes (2009) *Inorg.Chim.Acta,* **362,** 3860

Formula: $C_{17}\,H_{31}\,N_5\,O_2\,Zn_1{}^{2+},2(Cl_1\,O_4{}^{1-})$

Compound Name: (cdelta/Λ)-(trans-I-(++++)-1,4,7,10-Tetraazacyclododecane-N,N',N'',N''')-((S)-phenylalanine-O)-zinc(ii) diperchlorate

Space Group:	P1	***Cell:***	***a*** 10.330(0)	***b*** 11.368(0)	***c*** 11.397(0)
Space Group No.:	1	***(Å,°)***	α 69.16(0)	β 89.70(0)	γ 86.85(0)

R-Factor (%): 3.89 ***Temperature(K):*** 150 ***Density(g/cm³):*** 1.6

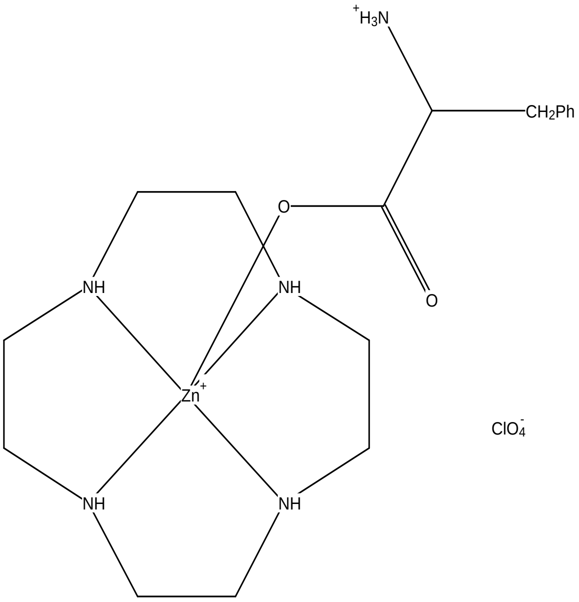

KUBTOR

Reference: Z.Vargova, R.Gyepes, L.Arabuli, K.Gyoryova, P.Hermann, I.Lukes (2009) *Inorg.Chim.Acta,* **362,** 3860

Formula: $C_{11}\,H_{27}\,N_5\,O_2\,Zn_1{}^{2+},2(Cl_1\,O_4{}^{1-})$

Compound Name: (Δ)-(trans-I-(++++)-1,4,7,10-Tetraazacyclododecane)-((S)-alanine)-zinc(ii) diperchlorate

Space Group:	P212121	***Cell:***	***a*** 8.675(0)	***b*** 8.834(0)	***c*** 27.698(0)
Space Group No.:	19	***(Å,°)***	α 90.00	β 90.00	γ 90.00

R-Factor (%): 5.64 ***Temperature(K):*** 150 ***Density(g/cm³):*** 1.645

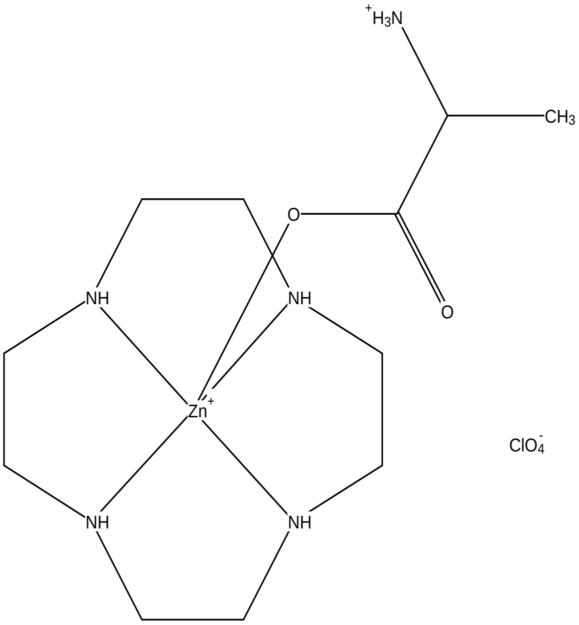

NUKFAB

Reference: F.A.Mautner, M.Mikuriya, H.Ishida, H.Sakiyama, F.R.Louka, J.W.Humphrey, S.S.Massoud (2009) *Inorg.Chim.Acta,* **362,** 4073

Formula: $C_{20}\,H_{40}\,N_{14}\,Ni_2{}^{2+},2(Cl_1\,O_4{}^{1-})$

Compound Name: bis((μ$_2$-1,5-dicyanamido)-(1,4,7,10-tetraazacyclododecane)-nickel) diperchlorate

Space Group:	P42/mnm	***Cell:***	***a*** 11.952(2)	***b*** 11.952(2)	***c*** 11.128(2)
Space Group No.:	136	***(Å,°)***	α 90.00	β 90.00	γ 90.00

R-Factor (%): 4.44 ***Temperature(K):*** 100 ***Density(g/cm³):*** 1.657

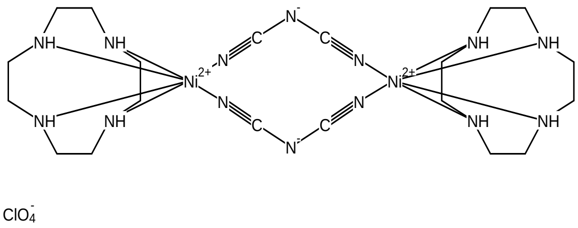

Search search1 (Mon May 04 10:12:16 2015): Hits 97-100

QUHHEH

Reference: S.S.Massoud, E.Druel, M.Dufort, R.Lalancette, J.Kitchen, J.Grebowicz, R.Vicente, U.Mukhopadhyay, I.Bernal, F.A.Mautner (2009) *Polyhedron,* **28,** 3849

Formula: $C_{18}\,H_{28}\,Cu_1\,N_6\,^{2+}$,2$(Cl_1\,O_4\,^{1-})$

Compound Name: (4,4'-Bipyridine)-(1,4,7,10-tetraazacyclododecane)-copper(ii) diperchlorate

Space Group: C2/c ***Cell:*** ***a*** 14.956(0) ***b*** 13.608(0) ***c*** 12.055(0)
Space Group No.: 15 ***(Å,°)*** α 90.00 β 91.92(0) γ 90.00

R-Factor (%): 3.23 ***Temperature(K):*** 100 ***Density(g/cm³):*** 1.601

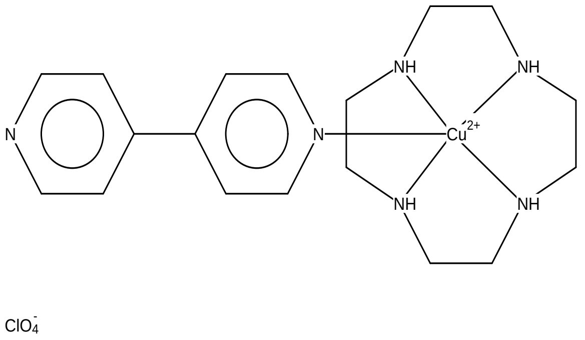

QUHHUX

Reference: S.S.Massoud, E.Druel, M.Dufort, R.Lalancette, J.Kitchen, J.Grebowicz, R.Vicente, U.Mukhopadhyay, I.Bernal, F.A.Mautner (2009) *Polyhedron,* **28,** 3849

Formula: $C_{28}\,H_{52}\,Cu_2\,N_{10}\,^{4+}$,4$(Cl_1\,O_4\,^{1-})$

Compound Name: (μ_2-1,2-bis(pyridin-4-yl)ethane)-bis(1,4,7,10-tetraazacyclododecane)-di-copper tetraperchlorate

Space Group: P-1 ***Cell:*** ***a*** 9.328(0) ***b*** 11.204(0) ***c*** 12.069(0)
Space Group No.: 2 ***(Å,°)*** α 63.55(0) β 74.45(0) γ 71.32(0)

R-Factor (%): 5.28 ***Temperature(K):*** 100 ***Density(g/cm³):*** 1.654

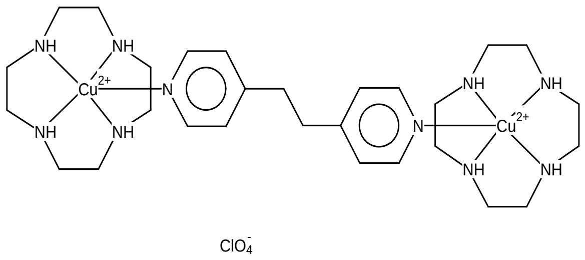

AGAMUR

Reference: Guangbin Wang, C.Slebodnick, G.T.Yee (2008) *Inorg.Chim.Acta,* **361,** 3593

Formula: $(C_{14}\,H_{20}\,Mn_1\,N_8\,^{1+})n,n(C_6\,N_4\,^{1-})$

Compound Name: catena-((μ_2-gem-Tetracyanoethylene radical-N,N')-(1,4,7,10-tetra-azacyclododecane)-manganese(ii) tetracyanoethylene radical anion)

Space Group: P212121 ***Cell:*** ***a*** 10.890(1) ***b*** 11.765(1) ***c*** 16.981(4)
Space Group No.: 19 ***(Å,°)*** α 90.00 β 90.00 γ 90.00

R-Factor (%): 5.1 ***Temperature(K):*** 100 ***Density(g/cm³):*** 1.476

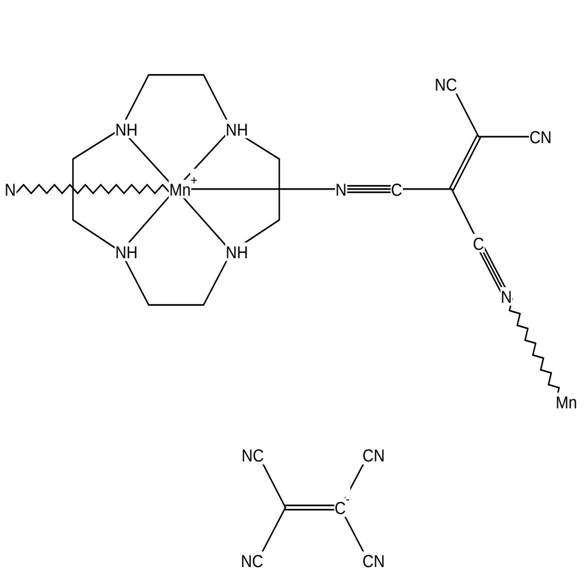

EGILAI

Reference: J.P.Hill, H.Palza, S.Alam, K.Ariga, A.L.Schumacher, F.D'Souza, C.E.Anson, A.K.Powell (2008) *Inorg.Chem.,* **47,** 8306

Formula: $C_{18}\,H_{43}\,Mn_2\,N_8\,O_3\,^{3+}$,3$(Cl_1\,O_4\,^{1-})$

Compound Name: (μ_2-Acetato)-(μ_2-oxo)-bis(1,4,7,10-tetra-azacyclododecane)-manganese(iii)-manganese(iv) triperchlorate

Space Group: Pca21 ***Cell:*** ***a*** 13.932(1) ***b*** 16.233(1) ***c*** 14.679(0)
Space Group No.: 29 ***(Å,°)*** α 90.00 β 90.00 γ 90.00

R-Factor (%): 4.77 ***Temperature(K):*** 200 ***Density(g/cm³):*** 1.656

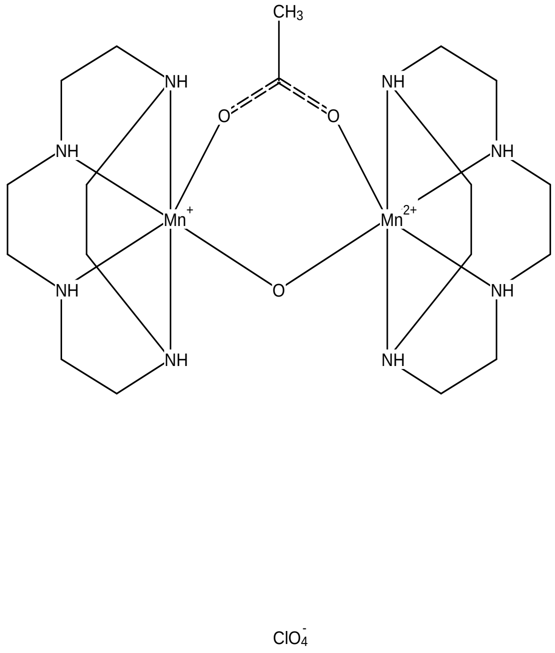

Search search1 (Mon May 04 10:12:16 2015): Hits 101-104

EGILEM

Reference: J.P.Hill, H.Palza, S.Alam, K.Ariga, A.L.Schumacher, F.D'Souza, C.E.Anson, A.K.Powell (2008) *Inorg.Chem.*,**47,**8306

Formula: $C_{16}\ H_{40}\ Mn_2\ N_8\ O_2\ ^{3+}, C_5\ Mn_1\ N_5\ S_5\ ^{3-}$

Compound Name: bis(μ_2-oxo)-bis(1,4,7,10-tetra-azacyclododecane)-manganese(iii)-manganese(iv) pentakis(isothiocyanato)-manganese

Space Group:	I2/a	***Cell:***	***a*** 18.270(1)	***b*** 11.238(0)	***c*** 18.643(0)
Space Group No.:	15	***(Å,°)***	α 90.00	β 114.51(0)	γ 90.00

R-Factor (%): 5.36 ***Temperature(K):*** 200 ***Density(g/cm³):*** 1.586

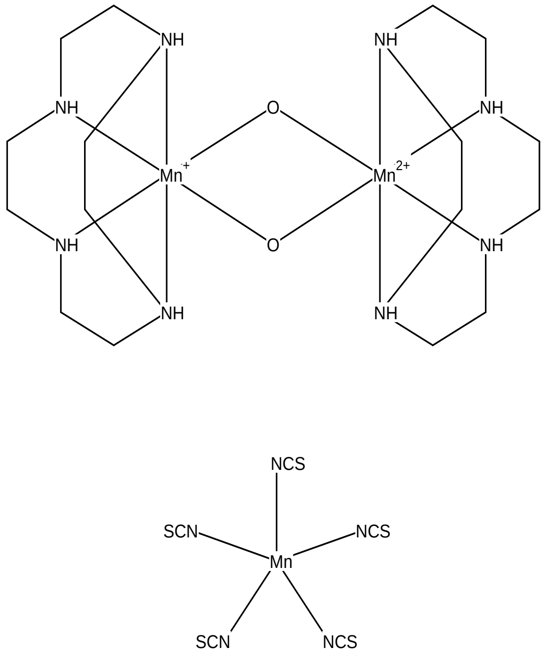

EGILIQ

Reference: J.P.Hill, H.Palza, S.Alam, K.Ariga, A.L.Schumacher, F.D'Souza, C.E.Anson, A.K.Powell (2008) *Inorg.Chem.*,**47,**8306

Formula: $(C_{16}\ H_{40}\ Mn_2\ N_8\ O_2\ ^{3+})2n,n(C_{14}\ Mn_4\ O_{28}\ ^{6-})_{,1}8.5n(H_2\ O_1)$

Compound Name: catena-(bis(bis(μ_2-oxo)-bis(1,4,7,10-tetra-azacyclododecane)-manganese(iii)-manganese(iv)) pentakis(μ_2-oxalato)-bis(oxalato)-tetra-manganese(ii) hydrate)

Space Group:	P-1	***Cell:***	***a*** 9.544(1)	***b*** 10.376(1)	***c*** 21.851(2)
Space Group No.:	2	***(Å,°)***	α 83.72(1)	β 80.11(1)	γ 85.46(1)

R-Factor (%): 4.98 ***Temperature(K):*** 200 ***Density(g/cm³):*** 1.682

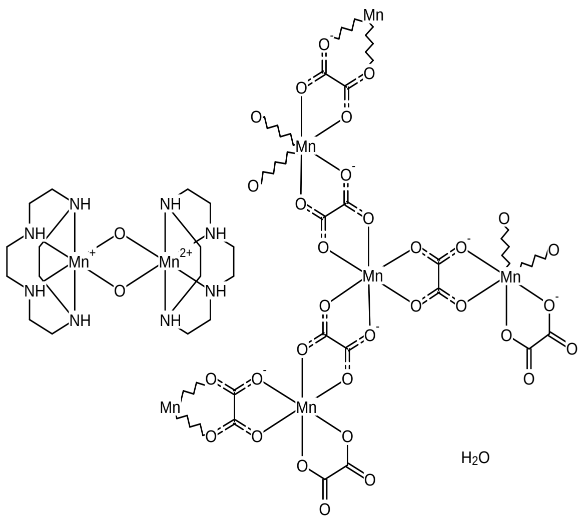

KITKUU01

Reference: J.P.Hill, H.Palza, S.Alam, K.Ariga, A.L.Schumacher, F.D'Souza, C.E.Anson, A.K.Powell (2008) *Inorg.Chem.*,**47,**8306

Formula: $C_{16}\ H_{40}\ Mn_2\ N_8\ O_2\ ^{3+}, 2(Cl_1\ ^{1-}), Cl_1\ O_4\ ^{1-}$

Compound Name: bis((μ_2-oxo)-(1,4,7,10-tetra-azacyclododecane))-manganese(iii)-manganese(iv) dichloride perchlorate

Space Group:	Pnma	***Cell:***	***a*** 22.385(1)	***b*** 9.193(0)	***c*** 13.242(1)
Space Group No.:	62	***(Å,°)***	α 90.00	β 90.00	γ 90.00

R-Factor (%): 4.8 ***Temperature(K):*** 200 ***Density(g/cm³):*** 1.601

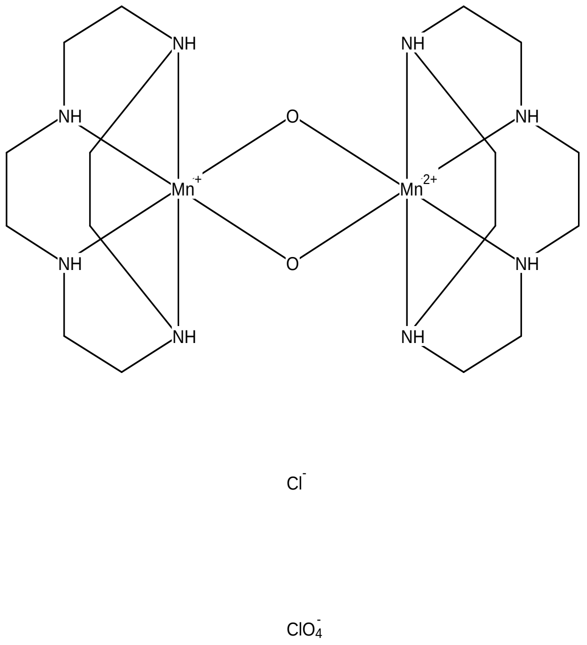

FOQBIX

Reference: Gao-mai Yang, Jun Li, Yan-wei Ren, Hui Guo, Min-yue Duan, Feng-xing Zhang, Xiongfu Zhang (2009) *Transition Met.Chem.*,**34,**191

Formula: $C_{20}\ H_{44}\ N_8\ Ni_2\ O_4\ ^{2+}, 2(Cl_1\ ^{1-}), 2(H_2\ O_1)$

Compound Name: (μ_2-Succinato-O,O',O'',O''')-bis(1,4,7,10-tetra-azacyclododecane-N,N',N'',N''')-di-nickel(ii) dichloride dihydrate

Space Group:	P21/n	***Cell:***	***a*** 7.843(2)	***b*** 13.159(4)	***c*** 15.011(4)
Space Group No.:	14	***(Å,°)***	α 90.00	β 99.20(0)	γ 90.00

R-Factor (%): 2.58 ***Temperature(K):*** 273 ***Density(g/cm³):*** 1.488

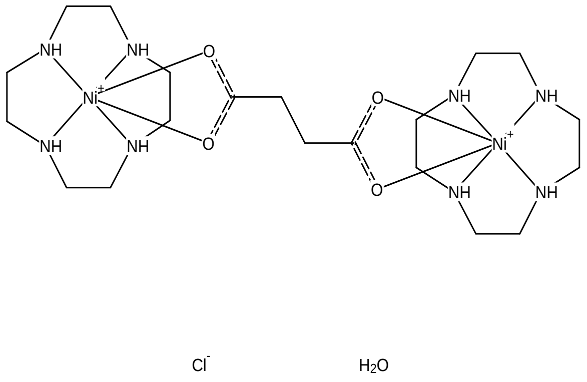

Search search1 (Mon May 04 10:12:16 2015): Hits 105-108

SOTMEU

Reference: A.El Majzoub, C.Cadiou, I.Dechamps-Olivier, F.Chuburu, M.Aplincourt, B.Tinant (2009) *Inorg.Chim.Acta,* **362,**1169

Formula: $C_{16}\,H_{27}\,Cu_1\,N_6{}^{2+}$,2($B_1\,F_4{}^{1-}$),$C_4\,H_{10}\,O_1$

Compound Name: (1-(1H-Benzimidazol-2-ylmethyl)-1,4,7,10-tetraazacyclododecane-N,N',N'',N''',N'''')-copper(ii) bis(tetrafluoroborate) diethyl ether solvate

Space Group:	P21/n	***Cell:***	***a*** 8.945(3)	***b*** 11.788(4)	***c*** 25.820(7)
Space Group No.:	14	***(Å,°)***	α 90.00	β 91.74(2)	γ 90.00

R-Factor (%): 4.24 ***Temperature(K):*** 120 ***Density(g/cm³):*** 1.5

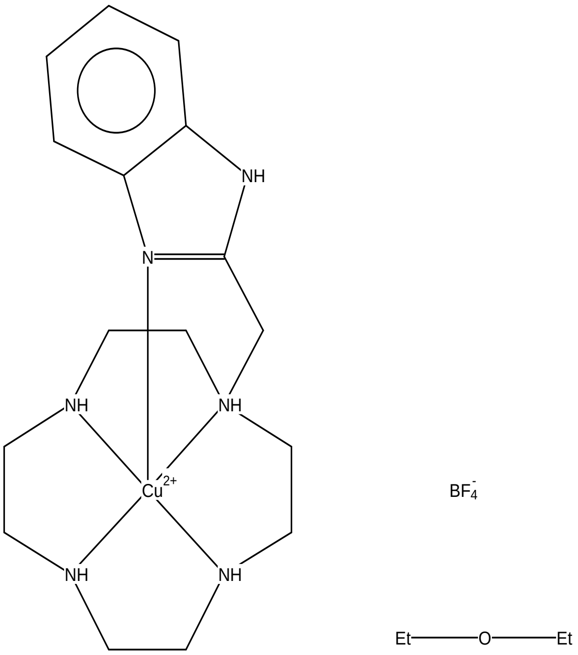

MIKBOY

Reference: Z.Vargova, J.Kotek, J.Rudovsky, J.Plutnar, R.Gyepes, P.Hermann, K.Gyoryova, I.Lukes (2007) *Eur.J.Inorg.Chem.,*3974

Formula: $C_8\,H_{20}\,N_5\,O_3\,Zn_1{}^{1+}$,$Cl_1\,O_4{}^{1-}$

Compound Name: (1,4,7,10-Tetraazacyclododecane)-nitrato-zinc(ii) perchlorate

Space Group:	P21/n	***Cell:***	***a*** 8.847(0)	***b*** 14.978(0)	***c*** 12.022(0)
Space Group No.:	14	***(Å,°)***	α 90.00	β 93.51(0)	γ 90.00

R-Factor (%): 4.71 ***Temperature(K):*** 293 ***Density(g/cm³):*** 1.667

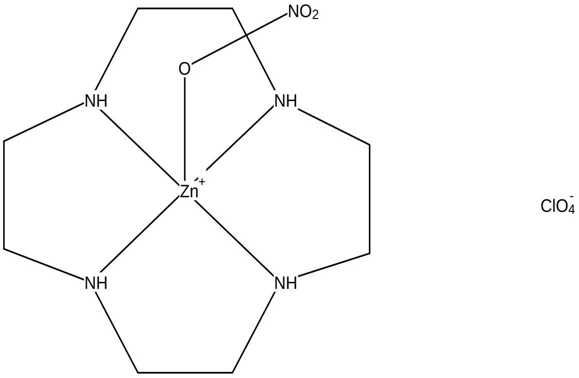

MIKBUE

Reference: Z.Vargova, J.Kotek, J.Rudovsky, J.Plutnar, R.Gyepes, P.Hermann, K.Gyoryova, I.Lukes (2007) *Eur.J.Inorg.Chem.,*3974

Formula: $C_{14}\,H_{26}\,N_6\,O_1\,Zn_1{}^{2+}$,2($Cl_1\,O_4{}^{1-}$)

Compound Name: (1,4,7,10-Tetraazacyclododecane)-(3-pyridinecarboxamide-N)-zinc(ii) diperchlorate

Synonym: (1,4,7,10-Tetraazacyclododecane)-(nicotinamide-N)-zinc(ii) diperchlorate

Space Group:	P21/n	***Cell:***	***a*** 8.803(0)	***b*** 18.718(0)	***c*** 13.332(0)
Space Group No.:	14	***(Å,°)***	α 90.00	β 96.06(0)	γ 90.00

R-Factor (%): 5.42 ***Temperature(K):*** 150 ***Density(g/cm³):*** 1.699

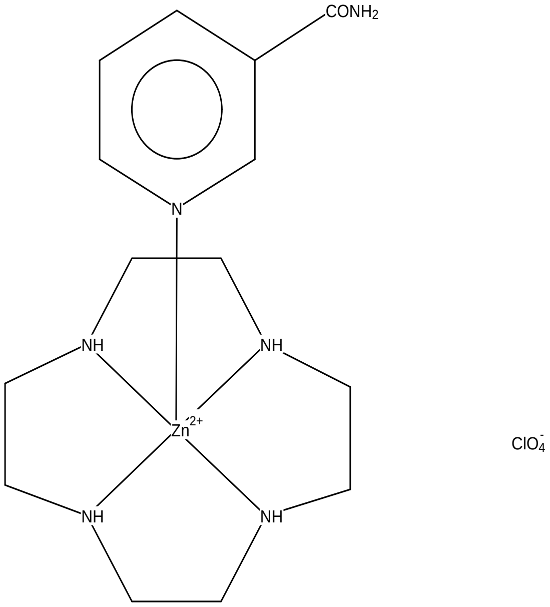

MIKCAL

Reference: Z.Vargova, J.Kotek, J.Rudovsky, J.Plutnar, R.Gyepes, P.Hermann, K.Gyoryova, I.Lukes (2007) *Eur.J.Inorg.Chem.,*3974

Formula: $C_{22}\,H_{44}\,N_9\,O_2\,Zn_2{}^{3+}$,3($Cl_1\,O_4{}^{1-}$),$H_2\,O_1$

Compound Name: (μ_2-Pyridine-2-carboxylato)-bis(1,4,7,10-tetraazacyclododecane)-di-zinc(ii) triperchlorate monohydrate

Synonym: (μ_2-Picolinato)-bis(1,4,7,10-tetraazacyclododecane)-di-zinc(ii) triperchlorate monohydrate

Space Group:	P21/c	***Cell:***	***a*** 8.692(0)	***b*** 22.919(0)	***c*** 18.945(0)
Space Group No.:	14	***(Å,°)***	α 90.00	β 90.75(0)	γ 90.00

R-Factor (%): 12.11 ***Temperature(K):*** 293 ***Density(g/cm³):*** 1.608

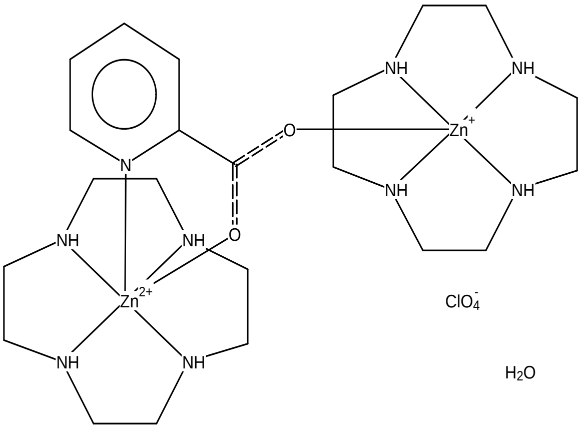

Search search1 (Mon May 04 10:12:16 2015): Hits 109-112

MIKCEP

Reference:	Z.Vargova, J.Kotek, J.Rudovsky, J.Plutnar, R.Gyepes, P.Hermann, K.Gyoryova, I.Lukes (2007) *Eur.J.Inorg.Chem.*,3974
Formula:	$C_{22}\ H_{44}\ N_9\ O_2\ Zn_2\ ^{3+}$,3($Cl_1\ O_4\ ^{1-}$)
Compound Name:	(μ_2-Pyridine-3-carboxylato)-bis(1,4,7,10-tetraazacyclododecane)-di-zinc(ii) triperchlorate
Synonym:	(μ_2-Nicotinato)-bis(1,4,7,10-tetraazacyclododecane)-di-zinc(ii) triperchlorate

Space Group:	P21/c	***Cell:***	***a*** 15.583(0)	***b*** 17.172(0)	***c*** 14.425(0)		
Space Group No.:	14	***(Å,°)***	α 90.00	β 114.49(0)	γ 90.00		
R-Factor (%):	3.91	***Temperature(K):***	150	***Density(g/cm³):***	1.694		

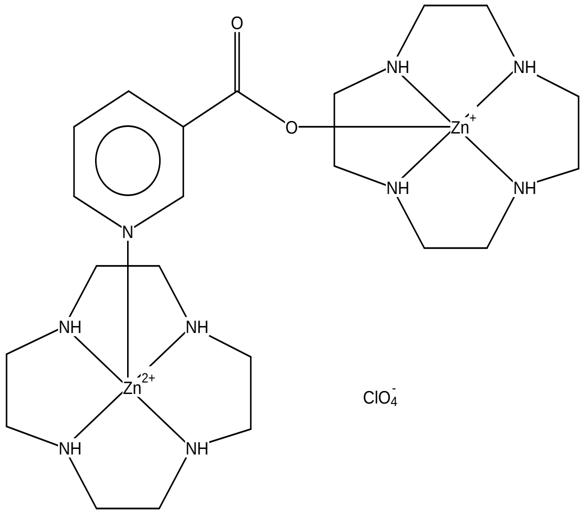

HINSIH

Reference:	B.M.Bartlett, T.D.Harris, M.W.DeGroot, J.R.Long (2007) *Z.Anorg.Allg.Chem.*,**633,**2380
Formula:	$C_{48}\ H_{80}\ B_2\ Fe_2\ N_{30}\ Ni_3\ ^{4+}$,4($B_1\ F_4\ ^{1-}$),4.5($H_2\ O_1$)
Compound Name:	hexakis(μ_2-Cyano)-bis(hydrogen tris(pyrazolyl)borato)-tris(1,4,7,10-tetraazacyclododecane)-di-iron(iii)-tri-nickel(ii) tetrakis(tetrafluoroborate) hydrate

Space Group:	I-43m	***Cell:***	***a*** 26.415(3)	***b*** 26.415(3)	***c*** 26.415(3)		
Space Group No.:	217	***(Å,°)***	α 90.00	β 90.00	γ 90.00		
R-Factor (%):	6.8	***Temperature(K):***	148	***Density(g/cm³):***	1.308		

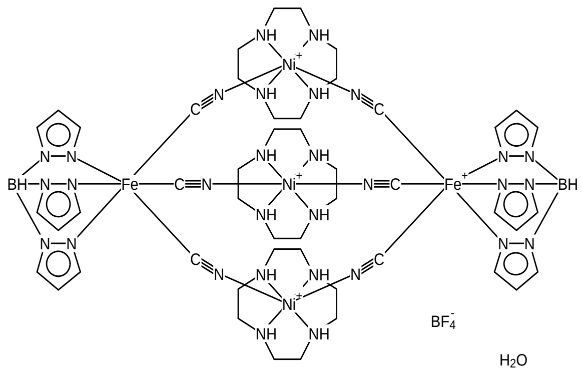

KITKUU

Reference:	J.P.Hill, S.Alam, K.Ariga, C.E.Anson, A.K.Powell (2008) *Chem.Commun.*,383
Formula:	$C_{16}\ H_{40}\ Mn_2\ N_8\ O_2\ ^{3+}$,2($Cl_1\ ^{1-}$),$Cl_1\ O_4\ ^{1-}$
Compound Name:	bis((μ_2-oxo)-(1,4,7,10-tetra-azacyclododecane))-manganese(iii)-manganese(iv) dichloride perchlorate

Space Group:	Pnma	***Cell:***	***a*** 22.385(1)	***b*** 9.193(0)	***c*** 13.242(1)		
Space Group No.:	62	***(Å,°)***	α 90.00	β 90.00	γ 90.00		
R-Factor (%):	4.8	***Temperature(K):***	200	***Density(g/cm³):***	1.601		

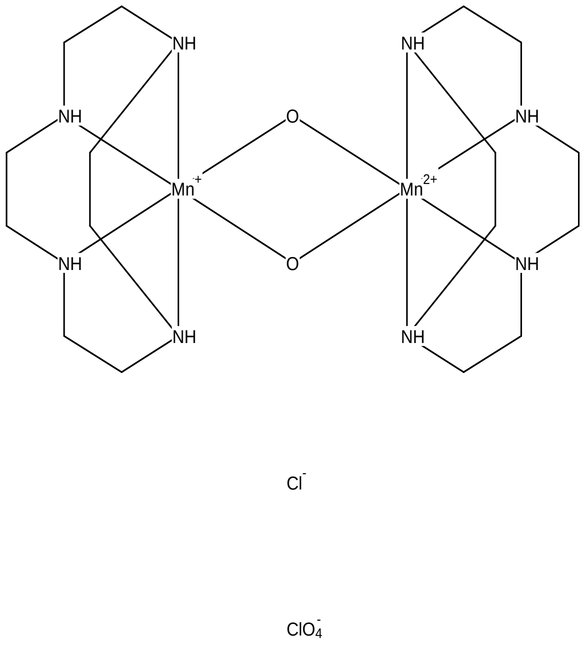

MIVQUE

Reference:	Jun-Fang Guo, Wai-Fun Yeung, Song Gao, Gene-Hsiang Lee, Shie-Ming Peng, M.H.-W.Lam, Tai-Chu Lau (2008) *Eur.J.Inorg.Chem.*,158
Formula:	($C_{12}\ H_{20}\ Cu_1\ Mn_1\ N_9$)n
Compound Name:	catena-(bis(μ_2-Cyano)-(1,4,7,10-tetraazacyclododecane)-dicyano-nitrido-copper(ii)-manganese(v))

Space Group:	P212121	***Cell:***	***a*** 7.627(0)	***b*** 12.809(0)	***c*** 17.087(0)		
Space Group No.:	19	***(Å,°)***	α 90.00	β 90.00	γ 90.00		
R-Factor (%):	5.23	***Temperature(K):***	150	***Density(g/cm³):***	1.627		

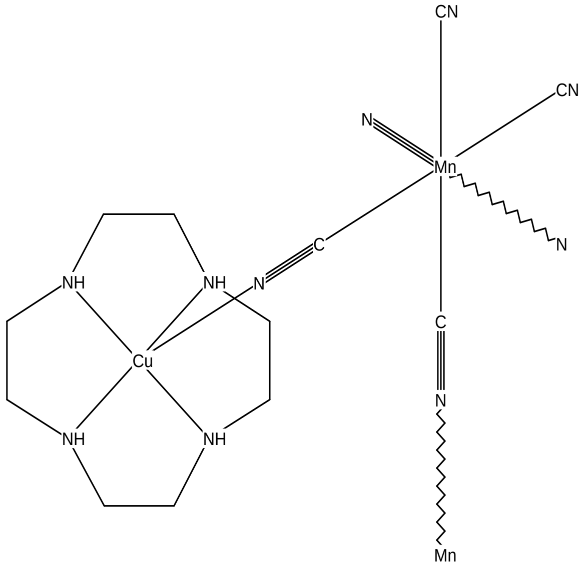

Search search1 (Mon May 04 10:12:16 2015): Hits 113-116

MIVRAL

Reference: Jun-Fang Guo, Wai-Fun Yeung, Song Gao, Gene-Hsiang Lee, Shie-Ming Peng, M.H.-W.Lam, Tai-Chu Lau (2008) *Eur.J.Inorg.Chem.*,158

Formula: $(C_{12} H_{20} Mn_1 N_9 Ni_1)n,n(C_1 H_4 O_1)$

Compound Name: catena-(bis(μ_2-Cyano)-(1,4,7,10-tetraazacyclododecane)-dicyano-nitrido-manganese(v)-nickel(ii) methanol solvate)

Space Group: C2/m
Space Group No.: 12
Cell: (Å,°) ***a*** 18.049(0) ***b*** 9.035(0) ***c*** 13.495(0) α 90.00 β 125.09(0) γ 90.00

R-Factor (%): 3.07 ***Temperature(K):*** 150 ***Density(g/cm3):*** 1.608

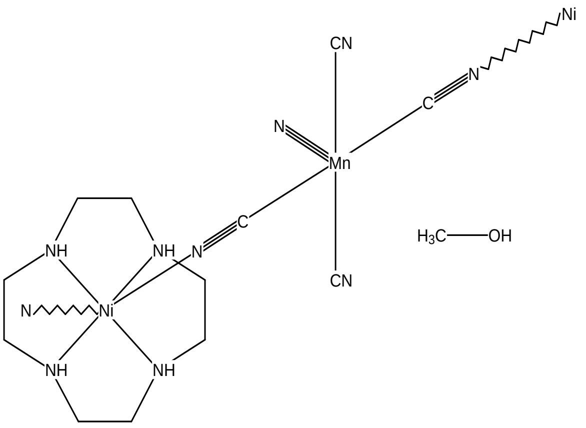

BAFJAV

Reference: E.A.Kovalenko, T.V.Mit'kina, O.A.Geras'ko, D.G.Samsonenko, D.Yu.Naumov, V.P.Fedin (2011) *Koord.Khim.*,**37,** 163

Formula: $C_8 H_{22} Cl_1 Co_1 N_4 O_1{}^{2+},2(Cl_1{}^{1-}),20(H_2 O_1),C_{48} H_{48} N_{32} O_{16}$

Compound Name: cis-(1,4,7,10-tetraazacyclododecane)-chloro-aqua-cobalt(iii) cucurbit(8)uril clathrate dichloride icosahydrate

Space Group: R-3
Space Group No.: 148
Cell: (Å,°) ***a*** 29.295(0) ***b*** 29.295(0) ***c*** 26.280(0) α 90.00 β 90.00 γ 120.00

R-Factor (%): 11.14 ***Temperature(K):*** 150 ***Density(g/cm3):*** 1.565

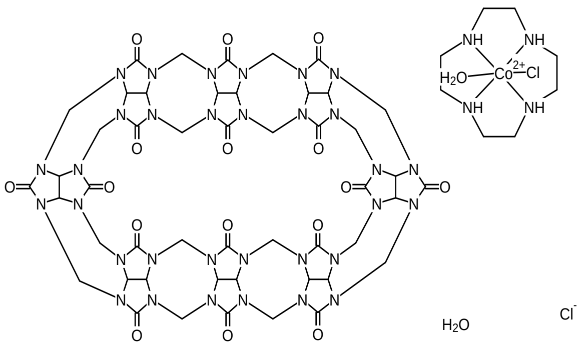

BAFJEZ

Reference: E.A.Kovalenko, T.V.Mit'kina, O.A.Geras'ko, D.G.Samsonenko, D.Yu.Naumov, V.P.Fedin (2011) *Koord.Khim.*,**37,** 163

Formula: $C_8 H_{22} Cl_1 N_4 Ni_1 O_1{}^{1+},C_{48} H_{48} N_{32} O_{16},Cl_1{}^{1-},12(H_2 O_1)$

Compound Name: cis-(1,4,7,10-Tetraazacyclododecane)-chloro-aqua-nickel(ii) cucurbit(8)uril clathrate chloride dodecahydrate

Space Group: R-3
Space Group No.: 148
Cell: (Å,°) ***a*** 38.995(3) ***b*** 38.995(3) ***c*** 13.698(2) α 90.00 β 90.00 γ 120.00

R-Factor (%): 14.45 ***Temperature(K):*** 120 ***Density(g/cm3):*** 1.545

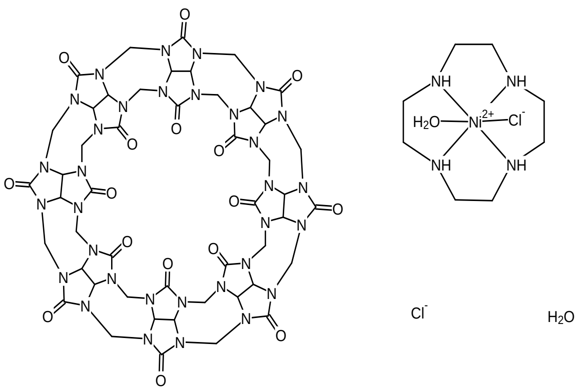

RAGJIU

Reference: E.A.Kovalenko, T.V.Mit'kina, O.A.Geras'ko, D.Yu.Naumov, D.G.Samsonenko, V.P.Fedin (2010) *Izv.Akad.Nauk SSSR,Ser.Khim.*, 2019

Formula: $6(H_3 O_1{}^{1+}),C_8 H_{20} Au_1 Cl_1 N_4{}^{2+},8(Cl_1{}^{1-}),18(H_2 O_1),C_{48} H_{48} N_{32} O_{16}$

Compound Name: hexakis(Hydronium) cucurbit(8)uril chloro-(1,4,8,11-tetraazacyclododecane)-gold(iii) clathrate octachloride octadecahydrate

Space Group: R-3
Space Group No.: 148
Cell: (Å,°) ***a*** 39.068(0) ***b*** 39.068(0) ***c*** 13.520(0) α 90.00 β 90.00 γ 120.00

R-Factor (%): 11.81 ***Temperature(K):*** 150 ***Density(g/cm3):*** 2.054

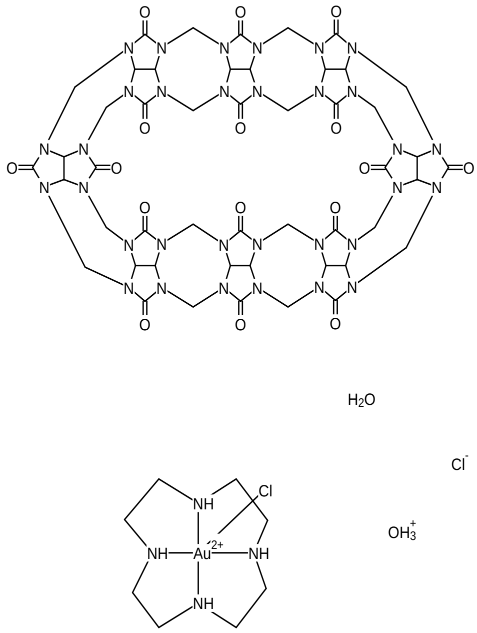

Supplement: Supplementary file 4 [file e-71-00693-sup4.pdf]
