# Supplementary material for: Crystal structures of (1,4,7,10-tetra­aza­cyclo­dodecane-κ4 N)bis­(tri­cyano­methanido-κN)nickel and (1,4,7,10-tetra­aza­cyclo­dodecane-κ4 N)(tri­cyano­methanido-κN)copper tri­cyano­methanide
Source: Acta Crystallogr E Crystallogr Commun. 2015 May 23;71(Pt 6):693–7. doi: 10.1107/S2056989015009524 (PMC4459349; doi:10.1107/S2056989015009524)

Search Overview

Search: search5

Date/Time done: Mon May 04 10:05:52 2015

Database(s):
CSD version 5.29 (November 2007)
CSD version 5.35 updates (Feb 2014)
CSD version 5.34 updates (Nov 2012)
CSD version 5.34 updates (Feb 2013)
CSD version 5.32 updates (Feb 2011)
CSD version 5.31 updates (Nov 2009)
CSD version 5.31 updates (Feb 2010)
CSD version 5.31 updates (May 2010)
CSD version 5.31 updates (Aug 2010)
CSD version 5.30 updates (Nov 2008)
CSD version 5.30 updates (Feb 2009)
CSD version 5.30 updates (May 2009)
CSD version 5.30 updates (Sep 2009)
CSD version 5.29 updates (Jan 2008)
CSD version 5.29 updates (Aug 2008)
CSD version 5.33 updates (Feb 2012)
CSD version 5.33 updates (May 2012)
CSD version 5.33 updates (Aug 2012)

Restriction Info: No refcode restrictions applied

Filters: None

Percentage Completed: 100%

Number of Hits: 23

Single query used. Search found structures that:

match
Query 1

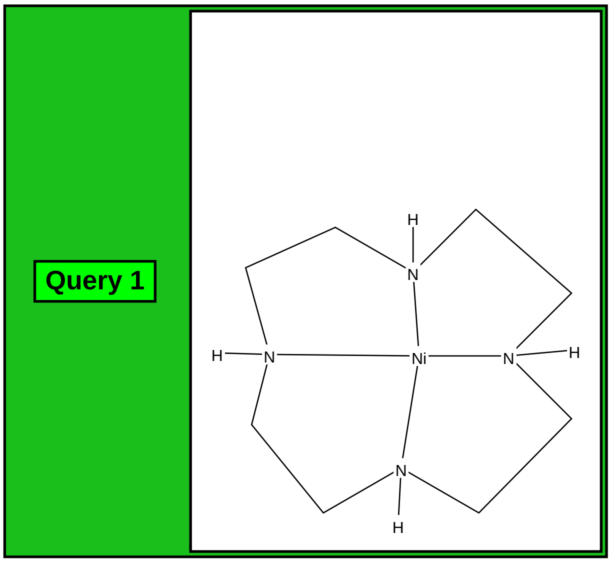

Search search5 (Mon May 04 10:05:52 2015): Hits 1-4

CEHBUN

Reference: Wai-Fun Yeung, Hoi-Ki Kwong, Tai-Chu Lau, Song Gao, Lap Szeto, Wing-Tak Wong (2006) *Polyhedron,* **25,**1256

Formula: C_{24} H_{40} N_{16} Ni_2 Pt_2,6(H_2 O_1)

Compound Name: tetrakis(μ_2-Cyano-C,N)-tetracyano-bis(1,4,7,10-tetra-azacyclododecane-N,N',N'',N''')-di-nickel(ii)-di-platinum hexahydrate

Space Group:	P21/c	***Cell:***	***a*** 8.680(3)	***b*** 14.218(3)	***c*** 15.758(4)
Space Group No.:	14	***(Å,°)***	α 90.00	β 93.69(3)	γ 90.00

R-Factor (%): 2.35 ***Temperature(K):*** 301 ***Density(g/cm³):*** 1.999

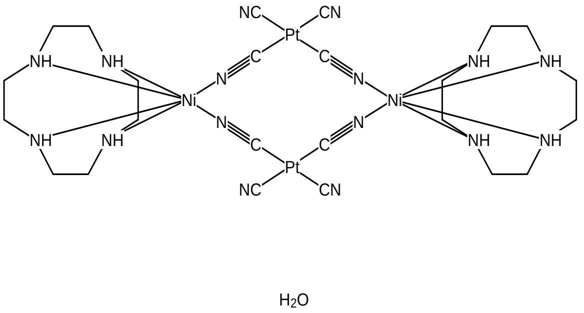

CEHCAU

Reference: Wai-Fun Yeung, Hoi-Ki Kwong, Tai-Chu Lau, Song Gao, Lap Szeto, Wing-Tak Wong (2006) *Polyhedron,* **25,**1256

Formula: C_{24} H_{40} N_{16} Ni_4,6(H_2 O_1)

Compound Name: tetrakis(μ_2-Cyano-C,N)-tetracyano-bis(1,4,7,10-tetra-azacyclododecane-N,N',N'',N''')-tetra-nickel(ii) hexahydrate

Space Group:	P21/c	***Cell:***	***a*** 8.643(2)	***b*** 14.218(4)	***c*** 15.542(5)
Space Group No.:	14	***(Å,°)***	α 90.00	β 93.96(1)	γ 90.00

R-Factor (%): 2.62 ***Temperature(K):*** 298 ***Density(g/cm³):*** 1.561

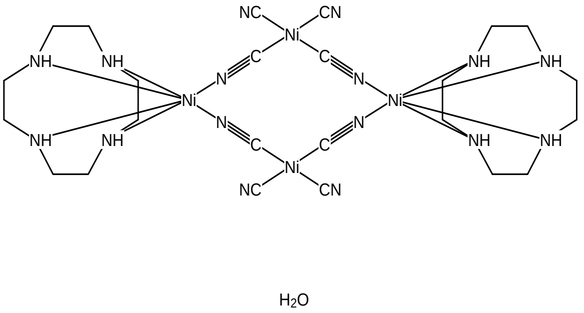

FABHEW

Reference: Tian-Huey Lu, K.Panneerselvam, Li-Hsuan Chen, Yung-Jan Lin, Fen-Ling Liao, Chung-Sun Chung (2001) *Anal.Sci.,* **17,** 571

Formula: C_{18} H_{28} N_6 Ni_1 $^{2+}$,2(Cl_1 O_4 $^{1-}$)

Compound Name: (2,2'-Bipyridyl)-(1,4,7,10-tetra-azacyclododecane)-nickel(ii) diperchlorate

Space Group:	Pbca	***Cell:***	***a*** 13.991(0)	***b*** 14.698(0)	***c*** 24.068(1)
Space Group No.:	61	***(Å,°)***	α 90.00	β 90.00	γ 90.00

R-Factor (%): 5.7 ***Temperature(K):*** 296 ***Density(g/cm³):*** 1.573

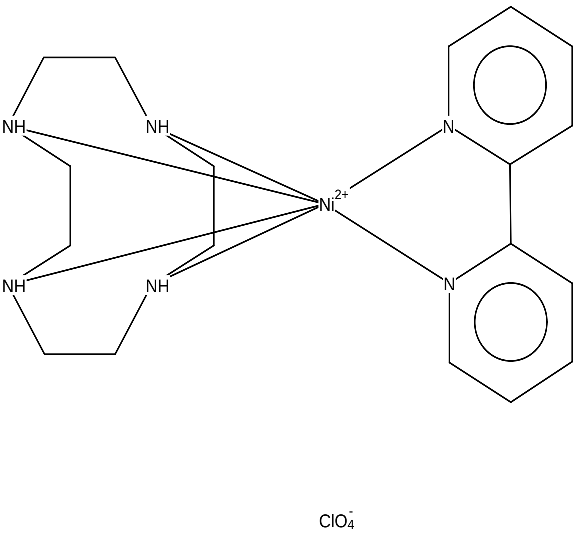

FAQBIJ

Reference: S.L.Heath, R.H.Laye, C.A.Muryn, N.Lima, R.Sessoli, R.Shaw, S.J.Teat, G.A.Timco, R.E.P.Winpenny (2004) *Angew.Chem.,Int.Ed.Engl.,* **43,**6132

Formula: C_{136} H_{258} Cr_{12} F_{16} N_8 Ni_3 O_{50},5.5(C_4 H_8 O_1),1.5(C_2 H_3 N_1)

Compound Name: hexadecakis(μ_2-Fluoro)-tetracosakis(μ_2-pivalato)-dihydroxy-bis(1,4,7,10-tetraazacyclododecane)-dodeca-chromium(iii)-tri-nickel(ii) tetrahydrofuran acetonitrile solvate

Space Group:	P-1	***Cell:***	***a*** 15.978(3)	***b*** 16.249(2)	***c*** 23.260(4)
Space Group No.:	2	***(Å,°)***	α 79.72(1)	β 72.17(1)	γ 84.78(1)

R-Factor (%): 11.06 ***Temperature(K):*** 100 ***Density(g/cm³):*** 1.283

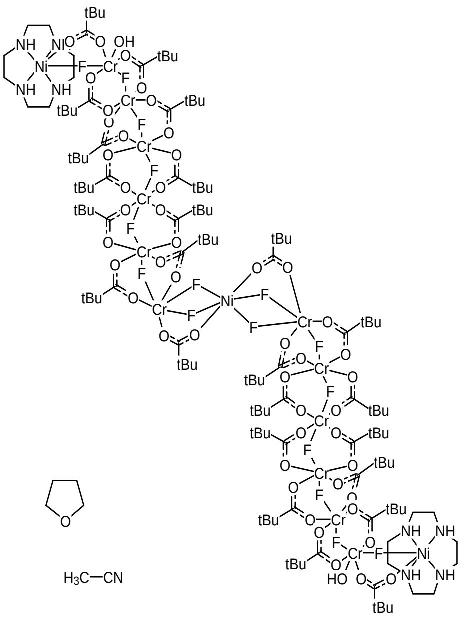

Search search5 (Mon May 04 10:05:52 2015): Hits 5-8

JIKJUI

Reference: B.Scott, K.J.Brewer, L.O.Spreer, C.A.Craig, J.W.Otvos, M.Calvin, S.Taylor (1990) *J.Coord.Chem.*,**21,**307

Formula: $C_8\ H_{24}\ N_4\ Ni_1\ O_2\ ^{2+}$,2($Cl_1\ O_4\ ^{1-}$),$H_2\ O_1$

Compound Name: Diaqua-(1,4,7,10-tetra-azacyclodecane)-nickel diperchlorate monohydrate

Space Group: P212121 ***Cell:*** ***a*** 11.173(5) ***b*** 11.976(5) ***c*** 13.969(4)
Space Group No.: 19 ***(Å,°)*** α 90.00 β 90.00 γ 90.00

R-Factor (%): 5.3 ***Temperature(K):*** 295 ***Density(g/cm^3):*** 1.72

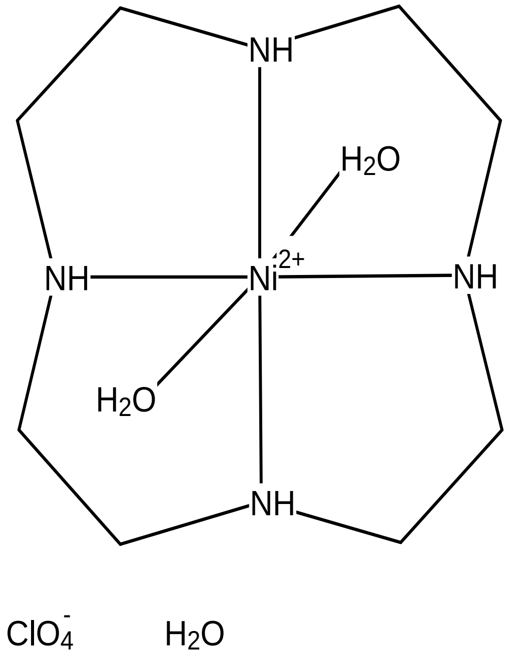

KEJDUZ

Reference: S.S.Massoud, F.A.Mautner, R.Vicente, B.M.Rodrigue (2006) *Inorg.Chim.Acta*,**359,**3321

Formula: $C_{24}\ H_{44}\ N_8\ Ni_2\ O_4\ ^{2+}$,2($Cl_1\ O_4\ ^{1-}$)

Compound Name: (μ_2-Terephthalato-O,O',O'',O''')-bis((1,4,7,10-tetraazacyclododecane)-nickel(ii)) diperchlorate

Space Group: Pnnm ***Cell:*** ***a*** 10.908(2) ***b*** 15.985(3) ***c*** 9.246(2)
Space Group No.: 58 ***(Å,°)*** α 90.00 β 90.00 γ 90.00

R-Factor (%): 4.19 ***Temperature(K):*** 100 ***Density(g/cm^3):*** 1.699

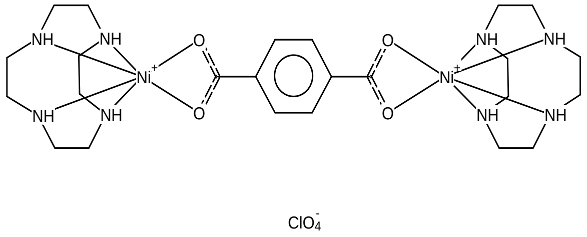

KEJMUH

Reference: A.Bencini, A.Bianchi, E.Garcia-Espana, Y.Jeannin, M.Julve, V.Marcelino, M.Philoche-Levisalles (1990) *Inorg.Chem.*,**29,** 963

Formula: 2($C_8\ H_{24}\ N_4\ Ni_1\ O_2\ ^{2+}$),$C_4\ O_4\ ^{2-}$,2($Cl_1\ O_4\ ^{1-}$)

Compound Name: bis(Diaqua-(1,4,7,10-tetra-azacyclododecane-N,N',N'',N''')-nickel(ii)) squarate diperchlorate

Space Group: P21cn ***Cell:*** ***a*** 11.124(3) ***b*** 11.461(3) ***c*** 25.735(6)
Space Group No.: 33 ***(Å,°)*** α 90.00 β 90.00 γ 90.00

R-Factor (%): 3.45 ***Temperature(K):*** 295 ***Density(g/cm^3):*** 1.711

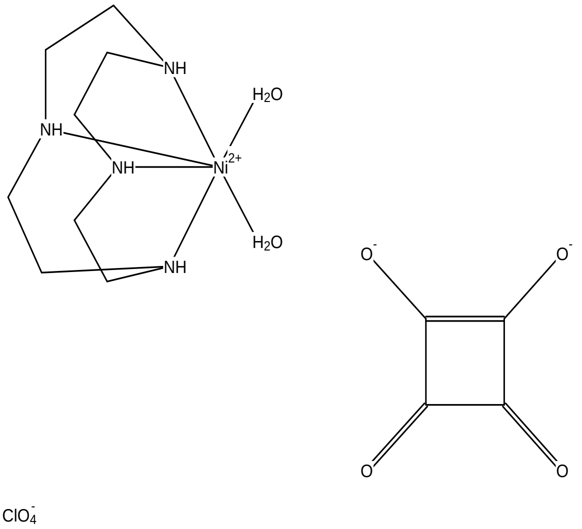

LEYWES

Reference: S.A.Baudron, M.W.Hosseini, N.Kyritsakas, M.Kurmoo (2007) *Discussion of Faraday Soc.*,1129

Formula: $C_{40}\ H_{52}\ N_{12}\ Ni_2\ Pd_1\ S_4\ ^{2+}$,2($B_1\ F_4\ ^{1-}$),6($C_3\ H_7\ N_1\ O_1$)

Compound Name: bis(μ_2-5H-pyrido[3',2':4,5]cyclopenta[1,2-b]pyridin-5-ylidenemethanedithiolato-N,N',S,S')-bis(1,4,7,10-tetra-azacyclododecane)-palladium-di-nickel(ii) bis(tetrafluoroborate) dimethylformamide solvate

Synonym: bis(μ_2-4,5-diazafluoren-9-ylidenemethanedithiolato-N,N',S,S')-bis(1,4,7,10-tetra-azacyclododecane)-palladium-di-nickel(ii) bis(tetrafluoroborate) dimethylformamide solvate

Space Group: P-1 ***Cell:*** ***a*** 8.479(0) ***b*** 8.666(0) ***c*** 25.747(1)
Space Group No.: 2 ***(Å,°)*** α 84.40(0) β 88.14(0) γ 80.47(0)

R-Factor (%): 6.65 ***Temperature(K):*** 173 ***Density(g/cm^3):*** 1.489

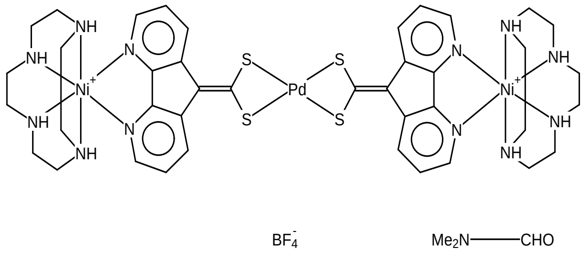

Search search5 (Mon May 04 10:05:52 2015): Hits 9-12

PAVYAN

Reference: Yan-Wei Ren, Jun Li, Su-Min Zhao, Feng-Xing Zhang (2005) *Struct.Chem.*,**16,**439

Formula: $C_8\,H_{24}\,N_4\,Ni_1\,O_2\,^{2+},C_8\,H_4\,O_4\,^{2-}$

Compound Name: cis-Diaqua-(1,4,7,10-tetra-azacyclotetradecane-N,N',N'',N''')-nickel(ii) terephthalate

Space Group: Ibca ***Cell:*** ***a*** 11.917(6) ***b*** 17.232(8) ***c*** 20.091(9)
Space Group No.: 73 ***(Å,°)*** α 90.00 β 90.00 γ 90.00

R-Factor (%): 5.55 ***Temperature(K):*** 298 ***Density(g/cm^3):*** 1.388

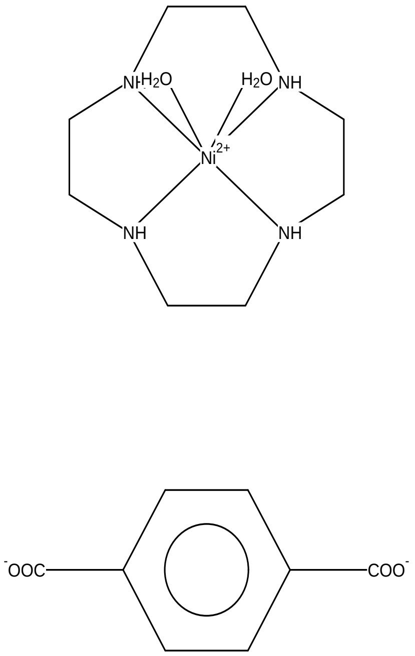

ULOBEC

Reference: Yung-Chan Lin, Tian-Huey Lu, Fen-Ling Liao, Chung-Sun Chung (2003) *Anal.Sci.*,**19,**967

Formula: $C_{20}\,H_{28}\,N_6\,Ni_1\,^{2+},2(Cl_1\,O_4\,^{1-})$

Compound Name: (1,10-Phenanthroline-N,N')-(1,4,7,10-tetra-azacyclododecane-N,N',N'',N''')-nickel(ii) diperchlorate

Space Group: Pbca ***Cell:*** ***a*** 14.144(1) ***b*** 14.923(1) ***c*** 24.191(2)
Space Group No.: 61 ***(Å,°)*** α 90.00 β 90.00 γ 90.00

R-Factor (%): 5.05 ***Temperature(K):*** 295 ***Density(g/cm^3):*** 1.587

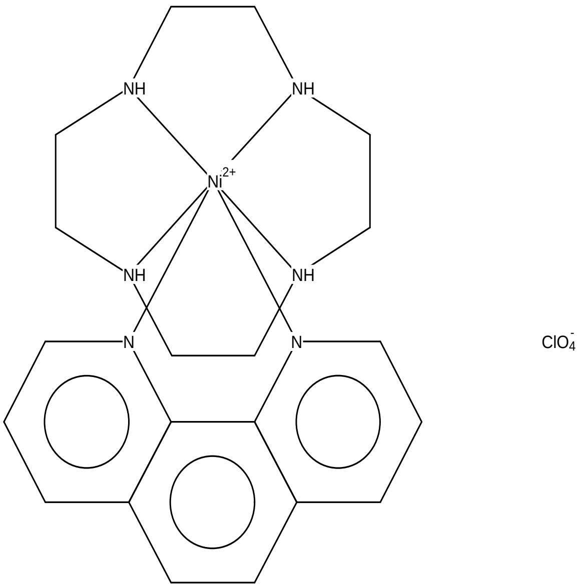

WUJLAO

Reference: I.P.Y.Shek, Wing-Tak Wong, Song Gao, Tai-Chu Lau (2002) *New J.Chem.(Nouv.J.Chim.)*,**26,**1099

Formula: $(C_{22}\,H_{40}\,Fe_1\,N_{14}\,Ni_2)n,8n(H_2\,O_1)$

Compound Name: catena-(bis(μ$_3$-Cyano)-tetracyano-bis(1,4,7,10-tetra-azacyclododecane)-iron(ii)-di-nickel(ii) octahydrate)

Space Group: P2/m ***Cell:*** ***a*** 12.048(2) ***b*** 17.729(2) ***c*** 9.155(1)
Space Group No.: 10 ***(Å,°)*** α 90.00 β 112.08(1) γ 90.00

R-Factor (%): 3.32 ***Temperature(K):*** 296 ***Density(g/cm^3):*** 1.499

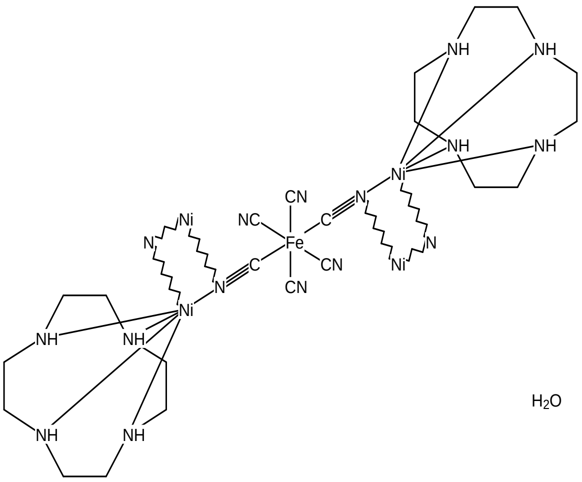

XADMAR

Reference: Jun Li, Yan-Wei Ren, Jin-Hua Zhang, Pin Yang (2004) *J.Chem.Cryst.*,**34,**409

Formula: $C_{10}\,H_{23}\,N_4\,Ni_1\,O_2\,^{1+},B_1\,F_4\,^{1-}$

Compound Name: (Acetato-O,O')-(1,4,7,10-tetra-azacyclododecane)-nickel(ii) tetrafluoroborate

Space Group: Pnnm ***Cell:*** ***a*** 12.045(5) ***b*** 14.906(6) ***c*** 8.913(4)
Space Group No.: 58 ***(Å,°)*** α 90.00 β 90.00 γ 90.00

R-Factor (%): 5.58 ***Temperature(K):*** 298 ***Density(g/cm^3):*** 1.564

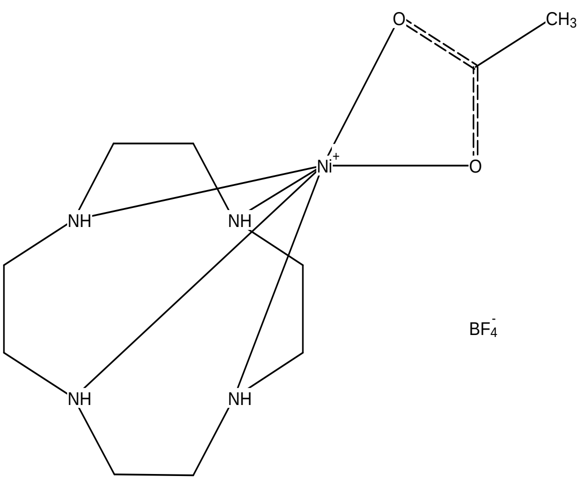

Search search5 (Mon May 04 10:05:52 2015): Hits 13-16

XEQTAP

Reference: Zhihui Zhang, Yanchun Lu, Jianhua Guo (2006) *J.Chem.Cryst.*,**36**,543

Formula: $(C_{18}\ H_{28}\ N_6\ Ni_1\ ^{2+})n,2n(Cl_1\ O_4\ ^{1-})$

Compound Name: catena-((μ_2-4,4'-Bipyridine-N,N')-(1,4,7,10-tetra-azadodecane)-nickel(ii) diperchlorate)

Space Group:	C2/c	***Cell:***	***a*** 10.534(1)	***b*** 14.565(2)	***c*** 16.131(3)
Space Group No.:	15	***(Å,°)***	α 90.00	β 102.80(0)	γ 90.00

R-Factor (%): 4.41 ***Temperature(K):*** 293 ***Density(g/cm^3):*** 1.613

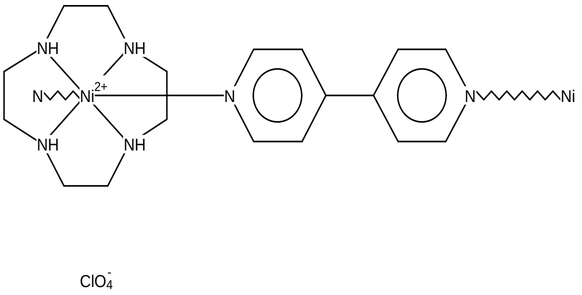

XIJKEG

Reference: C.Pariya, Ta-Yung Chi, T.K.Mishra, Chung-Sun Chung (2002) *Inorganic Chemistry Communications*,**5**,119

Formula: $C_{11}\ H_{24}\ Cl_1\ N_6\ Ni_1\ O_4\ ^{1+},Cl_1\ O_4\ ^{1-}$

Compound Name: (1,4,7,10-Tetraazacyclododecane)-(3-imidazolyl)-perchlorato-nickel(ii)

Space Group:	P21/n	***Cell:***	***a*** 8.899(0)	***b*** 16.185(0)	***c*** 13.658(0)
Space Group No.:	14	***(Å,°)***	α 90.00	β 94.81(0)	γ 90.00

R-Factor (%): 8.29 ***Temperature(K):*** 293 ***Density(g/cm^3):*** 1.687

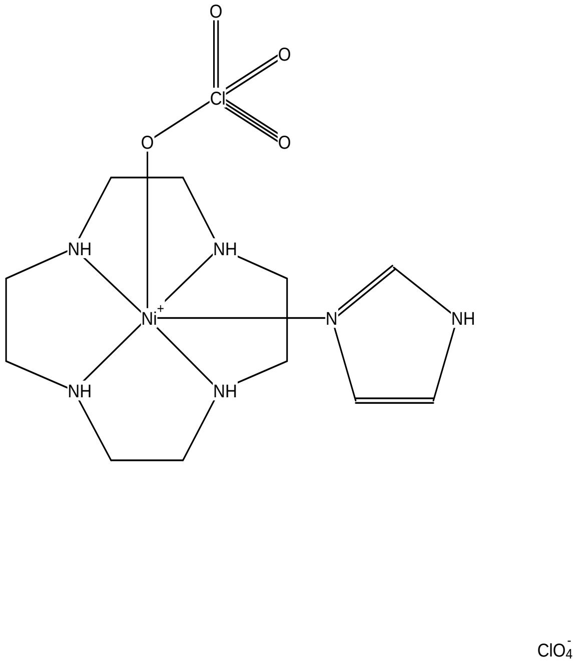

NURXII

Reference: Jun-Fang Guo, Xiu-Teng Wang, Bing-Wu Wang, Guan-Cheng Xu, Song Gao, Lap Szeto, Wing-Tak Wong, Wai-Yeung Wong, Tai-Chu Lau (2010) *Chemistry-A European Journal*,**16**,3524

Formula: $(C_{20}\ H_{34}\ N_6\ Ni_1\ O_4\ Ru_1\ ^{1+})n,n(C_1\ H_4\ O_1),n(Cl_1\ O_4\ ^{1-})$

Compound Name: catena-(bis(μ_2-cyanido)-bis(acetylacetonato)-(1,4,7,10-tetraazacyclododecane)-nickel(ii)-ruthenium(iii) perchlorate methanol solvate)

Space Group:	P21/c	***Cell:***	***a*** 12.316(0)	***b*** 14.925(0)	***c*** 16.736(1)
Space Group No.:	14	***(Å,°)***	α 90.00	β 100.07(0)	γ 90.00

R-Factor (%): 4.25 ***Temperature(K):*** 296 ***Density(g/cm^3):*** 1.565

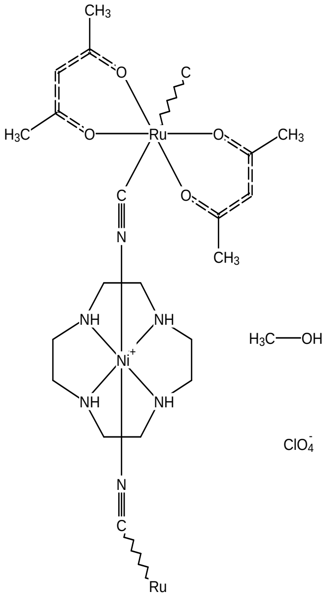

FOQBIX

Reference: Gao-mai Yang, Jun Li, Yan-wei Ren, Hui Guo, Min-yue Duan, Feng-xing Zhang, Xiongfu Zhang (2009) *Transition Met.Chem.*,**34**,191

Formula: $C_{20}\ H_{44}\ N_8\ Ni_2\ O_4\ ^{2+},2(Cl_1\ ^{1-}),2(H_2\ O_1)$

Compound Name: (μ_2-Succinato-O,O',O'',O''')-bis(1,4,7,10-tetra-azacyclododecane-N,N',N'',N''')-di-nickel(ii) dichloride dihydrate

Space Group:	P21/n	***Cell:***	***a*** 7.843(2)	***b*** 13.159(4)	***c*** 15.011(4)
Space Group No.:	14	***(Å,°)***	α 90.00	β 99.20(0)	γ 90.00

R-Factor (%): 2.58 ***Temperature(K):*** 273 ***Density(g/cm^3):*** 1.488

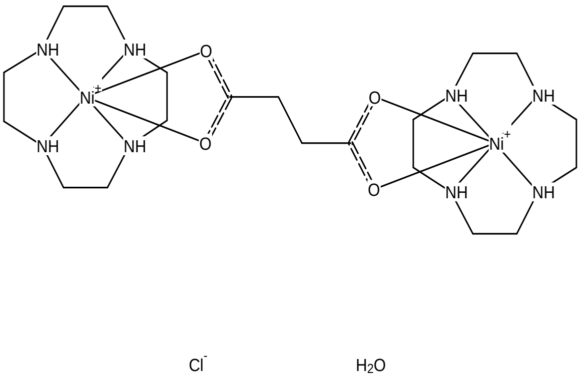

Search search5 (Mon May 04 10:05:52 2015): Hits 17-20

NUKFAB

Reference: F.A.Mautner, M.Mikuriya, H.Ishida, H.Sakiyama, F.R.Louka, J.W.Humphrey, S.S.Massoud (2009) *Inorg.Chim.Acta,* **362,** 4073

Formula: $C_{20}\ H_{40}\ N_{14}\ Ni_2\ ^{2+}$,2($Cl_1\ O_4\ ^{1-}$)

Compound Name: bis((μ_2-1,5-dicyanamido)-(1,4,7,10-tetraazacyclododecane)-nickel) diperchlorate

Space Group:	P42/mnm	***Cell:***	***a***	11.952(2)	***b***	11.952(2)	***c***	11.128(2)
Space Group No.:	136	***(Å,°)***	α	90.00	β	90.00	γ	90.00

R-Factor (%): 4.44 ***Temperature(K):*** 100 ***Density(g/cm³):*** 1.657

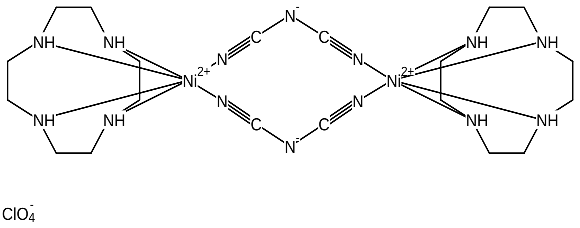

FOQBIX

Reference: Gao-mai Yang, Jun Li, Yan-wei Ren, Hui Guo, Min-yue Duan, Feng-xing Zhang, Xiongfu Zhang (2009) *Transition Met.Chem.,* **34,** 191

Formula: $C_{20}\ H_{44}\ N_8\ Ni_2\ O_4\ ^{2+}$,2($Cl_1\ ^{1-}$),2($H_2\ O_1$)

Compound Name: (μ_2-Succinato-O,O',O'',O''')-bis(1,4,7,10-tetra-azacyclododecane-N,N',N'',N''')-di-nickel(ii) dichloride dihydrate

Space Group:	P21/n	***Cell:***	***a***	7.843(2)	***b***	13.159(4)	***c***	15.011(4)
Space Group No.:	14	***(Å,°)***	α	90.00	β	99.20(0)	γ	90.00

R-Factor (%): 2.58 ***Temperature(K):*** 273 ***Density(g/cm³):*** 1.488

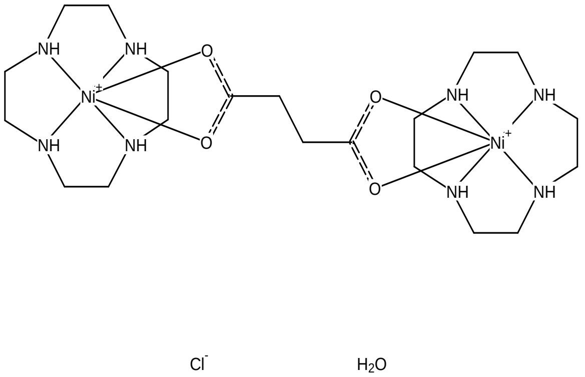

HINSIH

Reference: B.M.Bartlett, T.D.Harris, M.W.DeGroot, J.R.Long (2007) *Z.Anorg.Allg.Chem.,* **633,** 2380

Formula: $C_{48}\ H_{80}\ B_2\ Fe_2\ N_{30}\ Ni_3\ ^{4+}$,4($B_1\ F_4\ ^{1-}$),4.5($H_2\ O_1$)

Compound Name: hexakis(μ_2-Cyano)-bis(hydrogen tris(pyrazolyl)borato)-tris(1,4,7,10-tetraazacyclododecane)-di-iron(iii)-tri-nickel(ii) tetrakis(tetrafluoroborate) hydrate

Space Group:	I-43m	***Cell:***	***a***	26.415(3)	***b***	26.415(3)	***c***	26.415(3)
Space Group No.:	217	***(Å,°)***	α	90.00	β	90.00	γ	90.00

R-Factor (%): 6.8 ***Temperature(K):*** 148 ***Density(g/cm³):*** 1.308

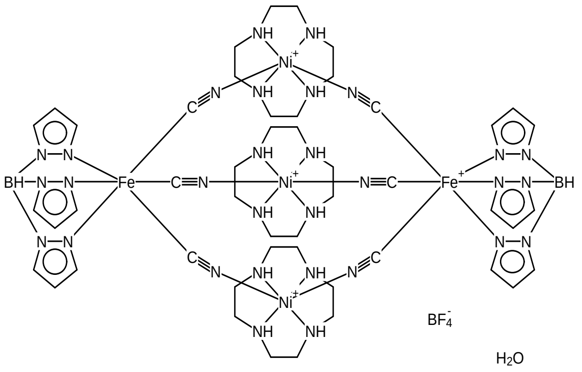

MIVRAL

Reference: Jun-Fang Guo, Wai-Fun Yeung, Song Gao, Gene-Hsiang Lee, Shie-Ming Peng, M.H.-W.Lam, Tai-Chu Lau (2008) *Eur.J.Inorg.Chem.,* 158

Formula: ($C_{12}\ H_{20}\ Mn_1\ N_9\ Ni_1$)n,n($C_1\ H_4\ O_1$)

Compound Name: catena-(bis(μ_2-Cyano)-(1,4,7,10-tetraazacyclododecane)-dicyano-nitrido-manganese(v)-nickel(ii) methanol solvate)

Space Group:	C2/m	***Cell:***	***a***	18.049(0)	***b***	9.035(0)	***c***	13.495(0)
Space Group No.:	12	***(Å,°)***	α	90.00	β	125.09(0)	γ	90.00

R-Factor (%): 3.07 ***Temperature(K):*** 150 ***Density(g/cm³):*** 1.608

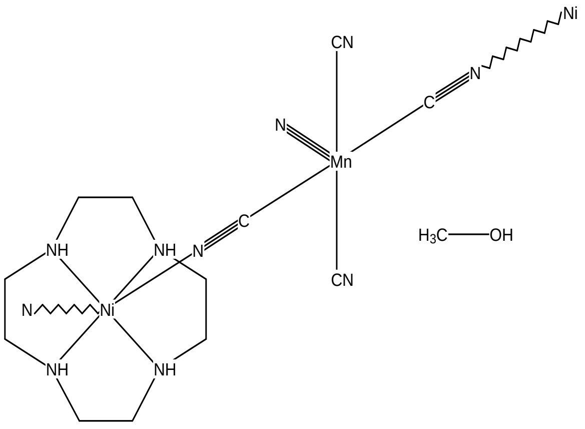

Search search5 (Mon May 04 10:05:52 2015): Hit 21

BAFJEZ

Reference:	E.A.Kovalenko, T.V.Mit'kina, O.A.Geras'ko, D.G.Samsonenko, D.Yu.Naumov, V.P.Fedin (2011) *Koord.Khim.*,**37**, 163
Formula:	$C_8 H_{22} Cl_1 N_4 Ni_1 O_1\ ^{1+}, C_{48} H_{48} N_{32} O_{16}, Cl_1\ ^{1-}, 12(H_2 O_1)$
Compound Name:	cis-(1,4,7,10-Tetraazacyclododecane)-chloro-aqua-nickel(ii) cucurbit(8) uril clathrate chloride dodecahydrate

Space Group:	R-3	***Cell:***	***a***	38.995(3)	***b***	38.995(3)	***c***	13.698(2)
Space Group No.:	148	***(Å,°)***	α	90.00	β	90.00	γ	120.00

R-Factor (%):	14.45	***Temperature(K):***	120	***Density(g/cm³):***	1.545

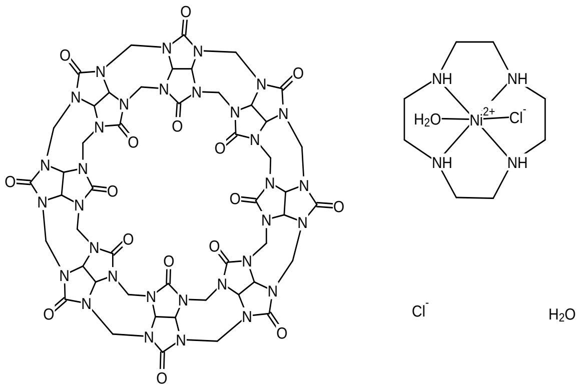

Supplement: Supplementary file 5 [file e-71-00693-sup5.pdf]
