# Supplementary material for: Crystal structures of (1,4,7,10-tetra­aza­cyclo­dodecane-κ4 N)bis­(tri­cyano­methanido-κN)nickel and (1,4,7,10-tetra­aza­cyclo­dodecane-κ4 N)(tri­cyano­methanido-κN)copper tri­cyano­methanide
Source: Acta Crystallogr E Crystallogr Commun. 2015 May 23;71(Pt 6):693–7. doi: 10.1107/S2056989015009524 (PMC4459349; doi:10.1107/S2056989015009524)

Search Overview

Search:	search4
Date/Time done:	Mon May 04 09:55:12 2015
Database(s):	CSD version 5.29 (November 2007)<br>CSD version 5.35 updates (Feb 2014)<br>CSD version 5.34 updates (Nov 2012)<br>CSD version 5.34 updates (Feb 2013)<br>CSD version 5.32 updates (Feb 2011)<br>CSD version 5.31 updates (Nov 2009)<br>CSD version 5.31 updates (Feb 2010)<br>CSD version 5.31 updates (May 2010)<br>CSD version 5.31 updates (Aug 2010)<br>CSD version 5.30 updates (Nov 2008)<br>CSD version 5.30 updates (Feb 2009)<br>CSD version 5.30 updates (May 2009)<br>CSD version 5.30 updates (Sep 2009)<br>CSD version 5.29 updates (Jan 2008)<br>CSD version 5.29 updates (Aug 2008)<br>CSD version 5.33 updates (Feb 2012)<br>CSD version 5.33 updates (May 2012)<br>CSD version 5.33 updates (Aug 2012)
Restriction Info:	No refcode restrictions applied
Filters:	None
Percentage Completed:	100%
Number of Hits:	11

Single query used. Search found structures that:

match
Query 1

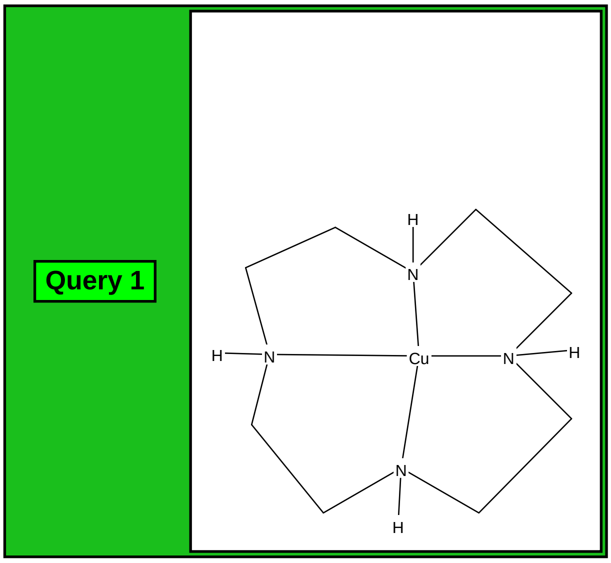

Search search4 (Mon May 04 09:55:12 2015): Hits 1-4

LISKON

Reference: Wai-Fun Yeung, Wing-Tak Wong, Jing-Lin Zuo, Tai-Chu Lau (2000) *J.Chem.Soc.,Dalton Trans.*,629

Formula: $C_{10}\,H_{20}\,Au_1\,Cu_1\,N_6\,^{1+}$,$C_2\,Au_1\,N_2\,^{1-}$

Compound Name: (μ_2-Cyano)-(1,4,7,10-tetra-azacyclododecane)-cyano-copper(ii)-gold(i) dicyano-gold(i)

Space Group: Pbcn ***Cell:*** ***a*** 7.267(1) ***b*** 31.143(2) ***c*** 16.758(2)
Space Group No.: 60 ***(Å,°)*** α 90.00 β 90.00 γ 90.00

R-Factor (%): 7.48 ***Temperature(K):*** 295 ***Density(g/cm^3):*** 2.57

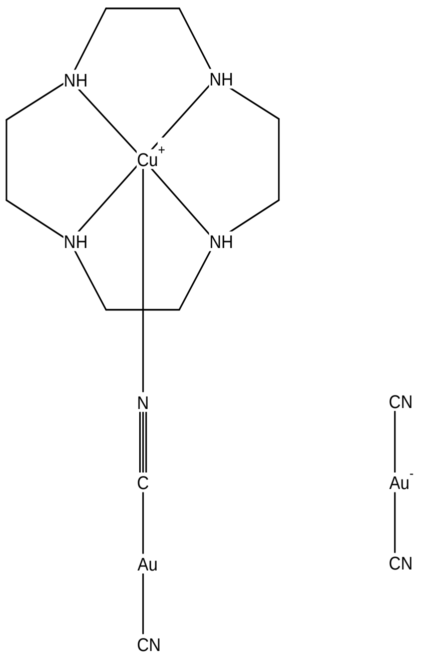

PAVYER

Reference: Yan-Wei Ren, Jun Li, Su-Min Zhao, Feng-Xing Zhang (2005) *Struct.Chem.*,**16,**439

Formula: $C_8\,H_{22}\,Cu_1\,N_4\,O_1\,^{2+}$,$C_8\,H_4\,O_4\,^{2-}$,3($H_2\,O_1$)

Compound Name: Aqua-(1,4,7,10-tetra-azacyclotetradecane-N,N',N'',N''')-copper(ii) terephthalate trihydrate

Space Group: P-1 ***Cell:*** ***a*** 7.815(3) ***b*** 10.137(4) ***c*** 14.460(5)
Space Group No.: 2 ***(Å,°)*** α 83.04(0) β 75.02(0) γ 73.87(0)

R-Factor (%): 4.68 ***Temperature(K):*** 273 ***Density(g/cm^3):*** 1.477

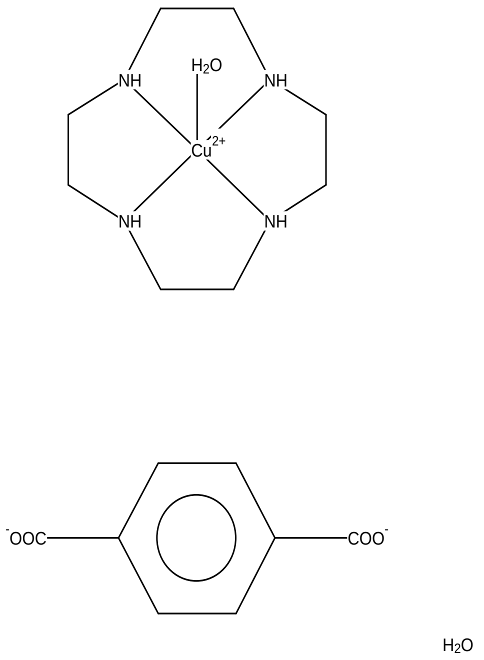

ROKLEI

Reference: Tian-Huey Lu, Jyh-Liong Lin, Wei-Jen Lan, Chung-Sun Chung (1997) *Acta Crystallogr.,Sect.C(Cr.Str.Comm.),* **53,**1598

Formula: 2($C_9\,H_{20}\,Cu_1\,N_5\,S_1\,^{1+}$),$C_4\,H_4\,Ca_1\,N_4\,O_2\,S_4\,^{2-}$,2($H_2\,O_1$)

Compound Name: bis((1,4,7,10-Tetra-azacyclododecane)-thiocyanato-copper(ii)) diaqua-tetraisothiocyanato-calcium dihydrate

Space Group: P21/c ***Cell:*** ***a*** 8.050(1) ***b*** 12.490(2) ***c*** 20.193(4)
Space Group No.: 14 ***(Å,°)*** α 90.00 β 95.97(1) γ 90.00

R-Factor (%): 2.6 ***Temperature(K):*** 295 ***Density(g/cm^3):*** 1.533

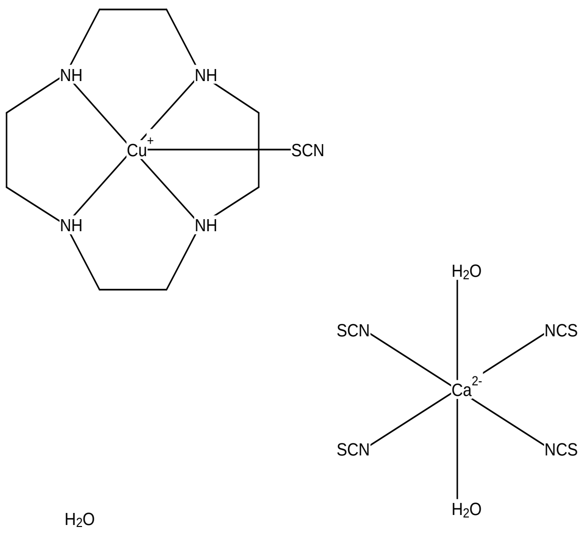

TZCDCU

Reference: R.Clay, P.Murray-Rust, J.Murray-Rust (1979) *Acta Crystallogr.,Sect.B*,**35,**1894

Formula: $C_8\,H_{20}\,Cu_1\,N_5\,O_3\,^{1+}$,$N_1\,O_3\,^{1-}$

Compound Name: Nitrato-(1,4,7,10-tetra-azacyclododecane)-copper(ii) nitrate

Space Group: P21/n ***Cell:*** ***a*** 12.000(10) ***b*** 13.760(10) ***c*** 8.860(20)
Space Group No.: 14 ***(Å,°)*** α 90.00 β 90.10(3) γ 90.00

R-Factor (%): 5.2 ***Temperature(K):*** 295 ***Density(g/cm^3):*** 1.634

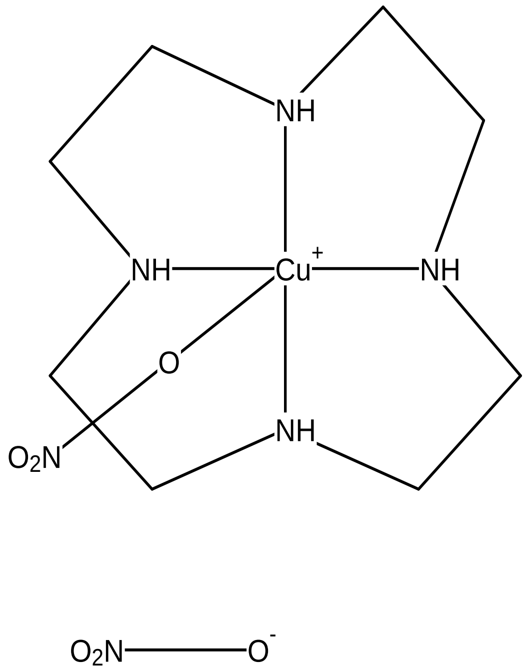

Search search4 (Mon May 04 09:55:12 2015): Hits 5-8

YAVQUH

Reference:	Soo-Young Kim, In-Sun Jung, Eunsung Lee, Jaheon Kim, S.Sakamoto, K.Yamaguchi, Kimoon Kim (2001) *Angew.Chem.,Int.Ed.Engl.*,**40,**2119
Formula:	$C_{48} H_{48} N_{32} O_{16}, C_8 H_{22} Cu_1 N_4 O_1{}^{2+}, 2(N_1 O_3{}^{1-}), 16(H_2 O_1)$
Compound Name:	Aqua-cucurbit(8)uril (1,4,7,10-tetra-azacyclododecane-copper(ii)) clathrate dinitrate hexadecahydrate

Space Group:	R-3	***Cell:***	***a***	29.858(0)	***b***	29.858(0)	***c***	24.876(0)
Space Group No.:	148	***(Å,°)***	α	90.00	β	90.00	γ	120.00
R-Factor (%):	10.44	***Temperature(K):***	188	***Density(g/cm³):***	1.553			

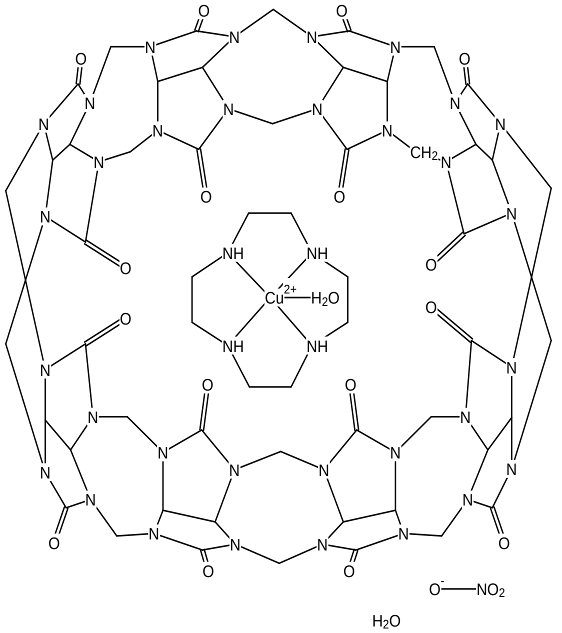

DUQXET

Reference:	D.Rohde, K.Merzweiler (2010) *Acta Crystallogr.,Sect.E(Structure Rep.Online)*,**66,**'m894'
Formula:	$C_8 H_{20} Cu_1 Mo_1 N_4 O_4, H_2 O_1$
Compound Name:	(μ_2-Oxo)-(1,4,7,10-tetra-azacyclododecane)-trioxido-copper(ii)-molybdenum monohydrate

Space Group:	P-1	***Cell:***	***a***	8.699(0)	***b***	8.978(0)	***c***	9.005(0)
Space Group No.:	2	***(Å,°)***	α	90.36(0)	β	91.95(0)	γ	100.74(0)
R-Factor (%):	2.39	***Temperature(K):***	200	***Density(g/cm³):***	1.99			

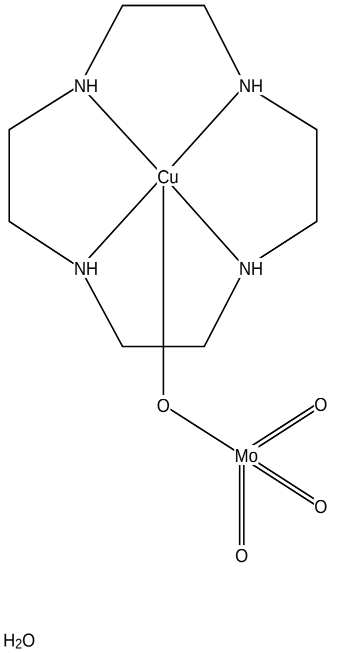

QUHHEH

Reference:	S.S.Massoud, E.Druel, M.Dufort, R.Lalancette, J.Kitchen, J.Grebowicz, R.Vicente, U.Mukhopadhyay, I.Bernal, F.A.Mautner (2009) *Polyhedron*,**28,**3849
Formula:	$C_{18} H_{28} Cu_1 N_6{}^{2+}, 2(Cl_1 O_4{}^{1-})$
Compound Name:	(4,4'-Bipyridine)-(1,4,7,10-tetraazacyclododecane)-copper(ii) diperchlorate

Space Group:	C2/c	***Cell:***	***a***	14.956(0)	***b***	13.608(0)	***c***	12.055(0)
Space Group No.:	15	***(Å,°)***	α	90.00	β	91.92(0)	γ	90.00
R-Factor (%):	3.23	***Temperature(K):***	100	***Density(g/cm³):***	1.601			

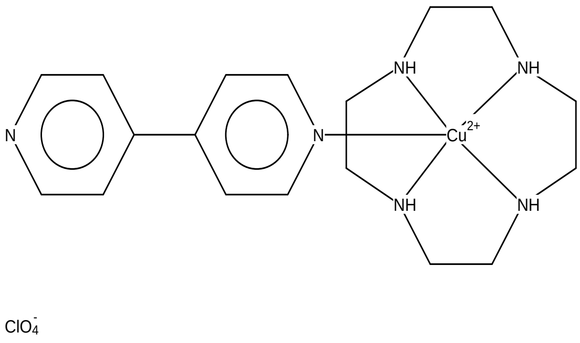

QUHHUX

Reference:	S.S.Massoud, E.Druel, M.Dufort, R.Lalancette, J.Kitchen, J.Grebowicz, R.Vicente, U.Mukhopadhyay, I.Bernal, F.A.Mautner (2009) *Polyhedron*,**28,**3849
Formula:	$C_{28} H_{52} Cu_2 N_{10}{}^{4+}, 4(Cl_1 O_4{}^{1-})$
Compound Name:	(μ_2-1,2-bis(pyridin-4-yl)ethane)-bis(1,4,7,10-tetraazacyclododecane)-di-copper tetraperchlorate

Space Group:	P-1	***Cell:***	***a***	9.328(0)	***b***	11.204(0)	***c***	12.069(0)
Space Group No.:	2	***(Å,°)***	α	63.55(0)	β	74.45(0)	γ	71.32(0)
R-Factor (%):	5.28	***Temperature(K):***	100	***Density(g/cm³):***	1.654			

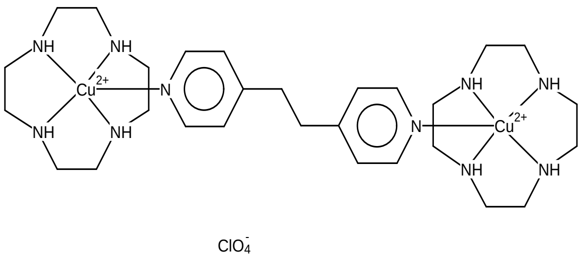

Search search4 (Mon May 04 09:55:12 2015): Hit 9

MIVQUE

Reference:	Jun-Fang Guo, Wai-Fun Yeung, Song Gao, Gene-Hsiang Lee, Shie-Ming Peng, M.H.-W.Lam, Tai-Chu Lau (2008) *Eur.J.Inorg.Chem.*,158
Formula:	(C_{12} H_{20} Cu_1 Mn_1 N_9)n
Compound Name:	catena-(bis(μ_2-Cyano)-(1,4,7,10-tetraazacyclododecane)-dicyano-nitrido-copper(ii)-manganese(v))

Space Group:	P212121	***Cell:***	***a*** 7.627(0)	***b*** 12.809(0)	***c*** 17.087(0)		
Space Group No.:	19	***(Å,°)***	α 90.00	β 90.00	γ 90.00		
R-Factor (%):	5.23	***Temperature(K):***	150	***Density(g/cm^3):***	1.627		

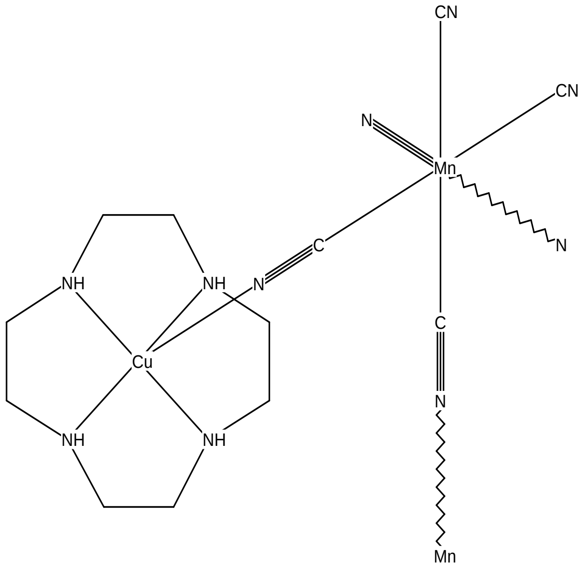

Supplement: Supplementary file 6 [file e-71-00693-sup6.pdf]
